# Host inflammatory dynamics reveal placental immune modulation by Group B *Streptococcus* during pregnancy

**DOI:** 10.15252/msb.202211021

**Published:** 2023-02-06

**Authors:** Felicia Kuperwaser, Gal Avital, Michelle J Vaz, Kristen N Noble, Allison N Dammann, Tara M Randis, David M Aronoff, Adam J Ratner, Itai Yanai

**Affiliations:** ^1^ Institute for Computational Medicine NYU Grossman School of Medicine New York NY USA; ^2^ Department of Pediatrics NYU Grossman School of Medicine New York NY USA; ^3^ Division of Neonatology, Department of Pediatrics Vanderbilt University Medical Center Nashville TN USA; ^4^ Renaissance School of Medicine at Stony Brook University Stony Brook NY USA; ^5^ Departments of Pediatrics and Molecular Medicine, Morsani School of Medicine University of South Florida FL Tampa USA; ^6^ Indiana University School of Medicine Indianapolis IN USA; ^7^ Department of Microbiology NYU Grossman School of Medicine New York NY USA; ^8^ Department of Biochemistry and Molecular Pharmacology NYU Grossman School of Medicine New York NY USA

**Keywords:** bacterial infection, group B *Streptococcus* (GBS), host–pathogen interactions, innate immunity, placenta, Immunology, Microbiology, Virology & Host Pathogen Interaction

## Abstract

Group B *Streptococcus* (GBS) is a pathobiont that can ascend to the placenta and cause adverse pregnancy outcomes, in part through production of the toxin β‐hemolysin/cytolysin (β‐h/c). Innate immune cells have been implicated in the response to GBS infection, but the impact of β‐h/c on their response is poorly defined. We show that GBS modulates innate immune cell states by subversion of host inflammation through β‐h/c, allowing worse outcomes. We used an ascending mouse model of GBS infection to measure placental cell state changes over time following infection with a β‐h/c‐deficient and isogenic wild type GBS strain. Transcriptomic analysis suggests that β‐h/c‐producing GBS elicit a worse phenotype through suppression of host inflammatory signaling in placental macrophages and neutrophils, and comparison of human placental macrophages infected with the same strains recapitulates these results. Our findings have implications for identification of new targets in GBS disease to support host defense against pathogenic challenge.

## Graphical abstract

Analyses of placental responses in an ascending infection model show that mice infected with a Group B *Streptococcus* (GBS) toxin knockout strain show a milder tissue‐level phenotype but their innate immune cells exhibit more inflammation than WT GBS‐infected counterparts.

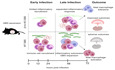

## Introduction

Infection during pregnancy is a leading cause of infant and maternal morbidity and mortality (Melin, 2011; Hall *et al*, 2017; Russell *et al*, 2017b; Shabayek & Spellerberg, 2018; Megli & Coyne, 2022). Group B *Streptococcus* (GBS) is a commensal gram positive bacterial species that asymptomatically colonizes about 25% of adults (Russell *et al*, 2017a; Shabayek & Spellerberg, 2018). During pregnancy, it can ascend the reproductive tract to cause chorioamnionitis, funisitis, preterm labor, and stillbirth (Whidbey *et al*, 2013; Hall *et al*, 2017). In cases of vertical transmission to the fetus, it can cause sepsis and death (Whidbey *et al*, 2013; Landwehr‐Kenzel & Henneke, 2014). In order to prevent complications due to GBS infection, pregnant women are screened in late pregnancy and intrapartum antibiotics are administered to colonized women, but because of limitations of this care, GBS infection is still a leading cause of morbidity and mortality in the United States in infants up to 3 months of age (Melin, 2011; Shabayek & Spellerberg, 2018). In GBS infection, the placenta is one of the early sites of host–pathogen interaction (Whidbey *et al*, 2013), and the placental response to this pathogen can be critically important in dictating infection outcomes.

The placenta is an immunologically unique organ in that it must protect the fetus from pathogens while maintaining maternal tolerance toward the semi‐allogeneic fetus (Whidbey *et al*, 2013; Mezouar *et al*, 2021). It balances these competing needs through tightly controlled inflammatory regulation. In addition to its barrier functions, the placenta is involved in numerous supportive processes for the fetus including metabolic function and respiratory exchange. Many immune cells present in the placenta are involved in these functions (Erlebacher, 2013; Vento‐Tormo *et al*, 2018; Mezouar *et al*, 2021).

Macrophages are plastic and heterogeneous cells, in which polarization state and function can differ depending on their environment and ontogeny (Locati *et al*, 2020). Macrophages in the placenta, which can be maternal or fetal in origin (Erlebacher, 2013; Yang *et al*, 2019), mediate different functions at different stages of pregnancy including phagocytosis of apoptotic cells, facilitation of invasion and remodeling and cytokine secretion, all of which are important to support a successful pregnancy (Erlebacher, 2013; Liu *et al*, 2017; Reyes & Golos, 2018; Ander *et al*, 2019; Yao *et al*, 2019; Mezouar *et al*, 2021). Throughout pregnancy, placental macrophages maintain a balance of pro‐ and anti‐inflammatory polarization states, and disruption of this balance has been associated with pathological outcomes of pregnancy including miscarriage, preeclampsia and preterm birth (Gonzalez *et al*, 2011; Schonkeren *et al*, 2011; Guenther *et al*, 2012; Erlebacher, 2013; Tsang *et al*, 2017; Vento‐Tormo *et al*, 2018; Pique‐Regi *et al*, 2019; Yang *et al*, 2019; Yao *et al*, 2019; Mezouar *et al*, 2021; Miller *et al*, 2022). Macrophages have also been implicated in the response to GBS infection in the placenta (Randis *et al*, 2014; Sutton *et al*, 2019), but their precise role in mediating these pathological outcomes is not fully defined.

The GBS toxin β‐hemolysin/cytolysin (β‐h/c) is a pore‐forming toxin with lytic activity whose expression by GBS is essential for the transition from commensal to pathogen and which is particularly important in mediating invasion into new physiological niches (Whidbey *et al*, 2013; Landwehr‐Kenzel & Henneke, 2014; Shabayek & Spellerberg, 2018). β‐h/c expression has been correlated with disease severity in different models of infection (Liu *et al*, 2004; Okumura & Nizet, 2014; Rosa‐Fraile *et al*, 2014), and while it has been shown to confer pathogenicity to GBS, the way that this toxin impacts GBS interactions with different cell types in a complex *in vivo* setting has not been fully elucidated.

Here, we defined the placental response to GBS infection by measuring phenotypic tissue level changes and transcriptomic cell type‐specific changes in a time course following infection. We focused on the role of β‐h/c in modulating the function of innate immune cell populations that mediate the placental response to GBS and how this critical interaction can lead to different outcomes. We found that while wild type (WT) GBS elicited a more severe phenotype than the β‐h/c knockout (KO) GBS, this response was paradoxically associated with a reduced inflammatory response in innate immune cells in the placenta. We propose that these findings reveal a GBS immune evasion strategy to cause severe infection by modulating placental innate immune responses.

## Results

### Ascending model of infection captures phenotypic differences in the response to GBS over time

**Figure 1:**
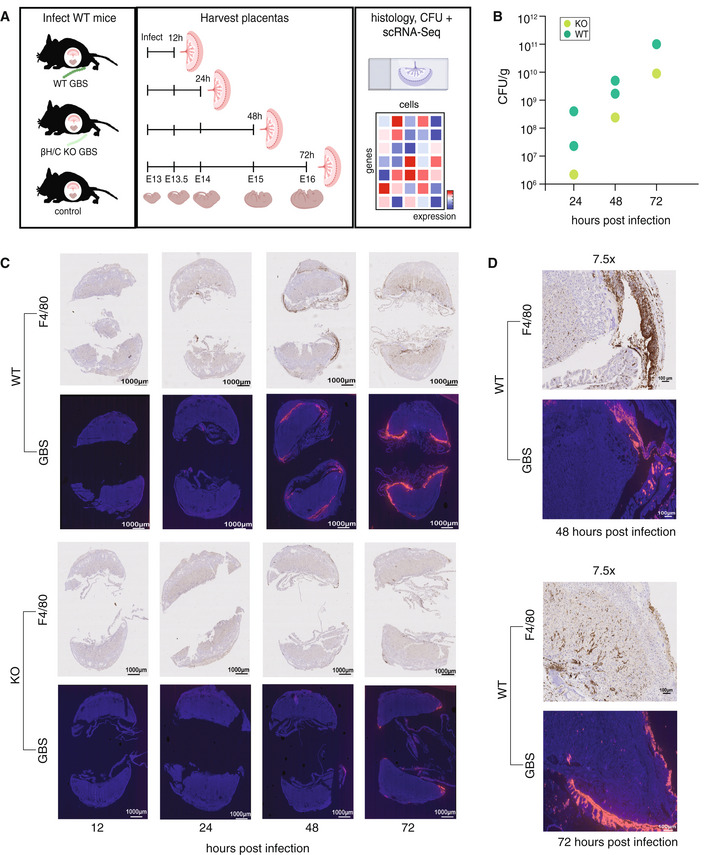
Ascending model of infection captures phenotypic response to GBS over time Schematic of the experimental approach. Pregnant mice were inoculated with β‐h/c KO (*n* = 4) or WT (*n* = 11) GBS or sham infected with PBS (*n* = 4). At four time points post infection, placentas were harvested and processed for tissue‐level phenotypic measurements and scRNA‐Seq (see Appendix Table S1).Bacterial load (CFU/g) of whole placentas cultured from GBS‐infected mice (see Materials and Methods). Data from sham‐infected mice is excluded because they were negative for GBS following culture. Data from mice infected for 12 h were also excluded because the individual placenta from the β‐h/c‐KO GBS‐infected mouse was not positive for GBS after culture, although other tissues from this mouse were.Immunofluorescent staining of GBS in fixed placental sections. Corresponding F4/80 histology (indicating macrophages) of sequential placental sections from the same samples.7.5X zoomed images of WT‐infected samples from 48 and 72 h post infection.

To investigate the placental response to GBS over time, we used an ascending mouse model of GBS infection (Randis *et al*, 2014) and measured the intrauterine response at different times after infection. In this model, pregnant C57BL/6 mice were intravaginally inoculated on pregnancy day E13 with WT or β‐h/c KO GBS or sham control. Placentas were harvested at four time points after inoculation between 12 and 72 h and were processed for phenotypic readouts including scRNA‐Seq (Fig 1A and Appendix Table S1).

Previous work using this model of ascending GBS infection has shown significantly greater placental inflammation and disruption of maternal–fetal barriers in WT GBS‐ rather than β‐h/c KO GBS‐infected mice, as well as the presence of intrauterine fetal demise and preterm birth only in WT GBS‐infected mice (Randis *et al*, 2014). We built on these results by first recapitulating these previously defined phenotypic differences in the placental response to these two GBS strains using tissue from the same mice processed for scRNA‐Seq (Appendix Table S1). To do so, we used two readouts: We measured bacterial burden in one placenta per mouse from each condition and found that at each time point, bacterial load, measured by CFU/g of placenta, was higher in WT‐infected than KO‐infected placentas, consistent with previous findings (Randis *et al*, 2014; Fig 1B, see Materials and Methods). We also found that the bacterial load increased within each condition at every time point relative to earlier samples, suggesting that both time post infection and presence of β‐h/c dictate the extent of bacterial burden in the placenta (Fig 1B).

We also measured GBS and macrophage presence in the tissue by immunofluorescent and histological staining. GBS staining revealed higher bacterial loads in the WT‐infected than the KO‐infected placentas at each time point, consistent with the culture results. Specifically, we observed infection along the fetal and maternal placental and decidual membranes with a corresponding increase in macrophage staining in the same heavily colonized tissue regions (Fig 1C and D, and Appendix Fig S1). These data confirm that β‐h/c KO GBS induces an attenuated phenotype relative to WT GBS and that macrophages play a role in mediating the response to GBS. These also suggest that the presentation of phenotype in both conditions increases at each time point following infection. Having recapitulated these tissue‐level phenotypic differences between conditions, we then asked which molecular programs underlie these phenotypic changes and how strain‐dependent differences in expression of these programs might lead to the observed tissue‐level differences.

### Global cell type analysis reveals immune lineage response at 48 h post infection

To define the dynamic molecular response to GBS in a cell type‐specific manner, we performed scRNA‐Seq on placentas from mice in each condition (Appendix Table S1). Since we observed the presentation of severe phenotype in mice beginning at 48 h post infection, including increased bacterial load and macrophage infiltration, we began analysis on all cells captured from samples 48 h post infection.

**Figure 2:**
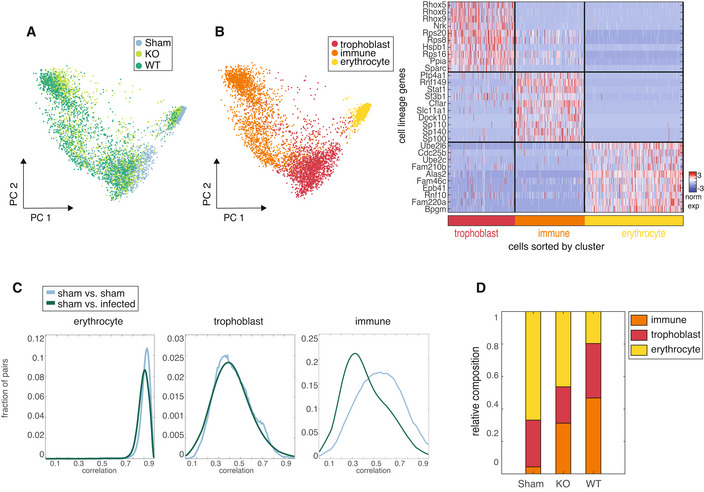
Global cell lineage analysis reveals an overall shift in immune lineage cells in response to infection PCA on all cells from 48 h after infection colored by treatment condition.PCA on all cells from 48 h after infection colored by clusters defined by hierarchical clusters (left). Z‐scored expression of differentially expressed and upregulated genes identified by Ranksum test (*P* < 0.0001) in each lineage cluster (right).Histograms of Pearson's correlation coefficients computed on variable genes on cell pairs between cells from sham‐ vs. GBS‐infected mice compared to cells from sham‐ vs. sham‐infected mice within each lineage.Bar plots of relative amount of cells from each condition per cell lineage cluster (immune cell absolute numbers: sham: 84/1,936 total cells, KO: 727/2,328 total cells, WT: 1,227/2,624 total cells).

Dimensionality reduction analysis using principal component analysis (PCA) on all cells from 48 h post infection revealed three main clusters. Each of these clusters included cells from different conditions, suggesting that cell type, rather than infection condition, was the main source of transcriptomic variance across these cells (Fig 2A and Appendix Fig S2A). We annotated these three groups as corresponding to the trophoblast, immune, and erythrocyte cell lineages, based on known cell type markers that were differentially expressed between clusters as well as independently curated marker genes (Fig 2B and Appendix Fig S2B). Sub‐clustering of immune and trophoblast lineages revealed the presence of multiple cell types, identified on the basis of uniquely expressed marker genes in each subcluster (Appendix Fig S2C–F).

To determine which cell lineage underwent the greatest transcriptomic changes due to infection, we compared cells within each lineage from sham‐ and GBS‐infected mice. For each lineage, we calculated the Pearson's correlation coefficient between every pair of cells from sham‐infected mice. We then calculated the correlation between each pair of cells from sham‐ and GBS‐infected mice and compared the distributions of correlations. We found that the erythrocyte and trophoblast lineages showed similar distributions of pairwise correlations between cells from sham‐ and GBS‐infected mice, but immune lineage cells from GBS‐infected mice were less correlated with equivalent cells from sham‐infected mice (Fig 2C). The relative composition of cell lineage types present in each condition shows that, as expected, infected mice have an influx of immune lineage cells (Fig 2D and Appendix Fig S2G). We note that immune cells from sham‐infected mice are not abundant at this time point, but given the large influx of immune cells in the infected mice, it is plausible that these cells are least correlated with placental immune cells present at steady state. On the basis of this shift in the immune cell population in infected mice, we focused our subsequent analysis on characterizing these responses.

We asked whether identified cell lineage subclusters also underwent transcriptional changes in response to infection even if those changes are not reflected on a whole‐lineage level. To answer this, we identified differentially expressed genes between cells from GBS‐ and sham‐infected mice within each subcluster and measured the functional enrichment of the infection‐specific genes within each subtype using Gene Ontology (GO; Appendix Fig S2H). Cells annotated to the Endodermal *Afp* High subcluster showed no significantly upregulated genes between conditions and were therefore excluded from functional enrichment analysis. The erythrocyte lineage did not include discrete subtypes, but analysis of differentially expressed genes between conditions revealed an enrichment in inflammatory expression in GBS‐infected erythrocyte lineage cells (Appendix Fig S2I). Immune lineage subtype changes will be discussed in subsequent analyses.

### Neutrophils from β‐h/c KO GBS‐infected mice show higher inflammatory response than those from WT GBS‐infected mice at the time of severe phenotype

**Figure 3:**
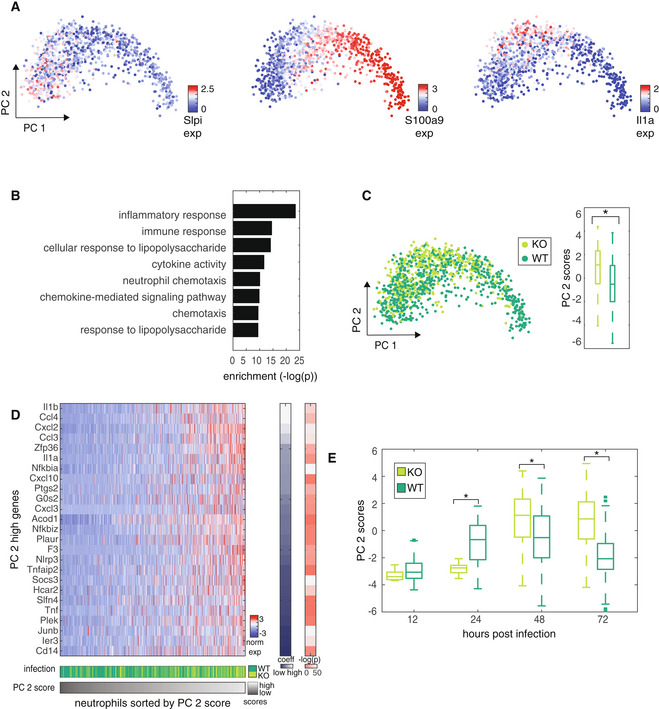
Neutrophil analysis reveals difference in response dynamics between strains PCA on neutrophils from 48 h post infection colored by expression of marker genes of neutrophil subpopulations (Han *et al*, 2018; left, middle) and a representative PC 2 high gene (right).GO enrichments of the 60 genes that contribute the most to high PC 2 scores.PCA on neutrophils from 48 h post infection colored by infection condition (left) and boxplot of PC 2 scores from cells in each condition. Central band of the boxplot shows the median, boxes represent the IQR, and whiskers represent the most extreme values not considered outliers. Significance was calculated by paired *t*‐test (right, *P < 10^−26^). The numbers of cells (biological replicates) included in the boxplots (KO, WT) are 418 and 700.Z‐scored expression of genes contributing to high PC 2 scores. Neutrophils from 48 h post infection are ordered by PC 2 score (bottom bar) and infection condition is indicated in middle bar. Coefficient of each gene is indicated in the bar on the right, and −log(*P*) of expression difference of that gene between KO‐ and WT‐infected cells determined by paired *t*‐test is indicated in the rightmost bar.Boxplots of neutrophil PC 2 scores from cells at each time point between conditions. Central band of the boxplot shows the median, boxes represent the IQR, and whiskers represent the most extreme values not considered outliers. Significance was calculated by paired *t*‐test (12 h: NS, 24 h: *P < 10^−05^, 48 h: *P < 10^−26^, 72 h: *P < 10^−84^). The numbers of cells (biological replicates) included in the boxplots (KO, WT) are 11, 70 (12H); 13, 270 (24H); 418, 700 (48H); 912, 265 (72H).

Since we found immune cells to be significantly modulated by infection, we next asked whether cell state changes differ depending on the GBS strain. We first focused on neutrophils and omitted cells from sham‐infected mice since they were relatively rare. PCA showed that PC 1 captured the distinction between two neutrophil subgroups present in the placenta (Han *et al*, 2018; Fig 3A). Consistent with the characterization of these as previously described distinct neutrophil subtypes, we found that *S100a9* and *Slpi* (together with other markers, Appendix Fig S3A) showed mutually exclusive expression. We did not find a significant difference in the distribution of PC 1 scores between cells from KO‐ and WT‐infected samples (Appendix Fig S3B), and cell cycle did not dictate the major source of variance captured by PCA (Appendix Fig S3C).

We next asked what the variance captured by PC 2 corresponds to and found that PC 2 is characterized by high expression of inflammatory genes, illustrated by expression of *Il1ɑ* in cells with high PC 2 scores (Fig 3A). GO analysis on genes that contribute to PC 2 showed an enrichment in inflammatory functions, confirming the characterization of these genes (Fig 3B). Given the tissue‐level differences between conditions (Fig 1), we expected greater inflammatory expression in the WT‐infected neutrophils. However, we observed that PC 2 scores, representing inflammatory gene expression, were higher in cells from KO‐infected than WT‐infected mice (Fig 3C and D, and Appendix Fig S3D and Appendix Table S2). Measurement of the differentially expressed genes between conditions further supported this observation (Appendix Fig S3E).

We considered the possibility that the apparent discrepancy observed between transcriptomic and tissue‐level data was not representative of the response across the entire dataset. To address this, we compared PC 2 scores between neutrophils from each condition at each time point (Fig 3E and Appendix Fig S3F, see Materials and Methods). We found that the inflammatory neutrophil dynamics captured by PC 2 scores are not consistent with phenotypic trends over time between conditions: In the early phase post infection (before 48 h), neutrophils from WT‐infected mice have higher inflammatory expression, indicated by PC 2 scores, than cells from matched KO‐infected mice, as we had originally expected (Fig 3E) based on the more severe phenotype in WT‐ than KO‐infected samples at each time point measured by histology and bacterial load (Fig 1). Next, when severe phenotype presents (48 h), there is an unexpected shift in relative inflammatory expression between conditions. Neutrophils from KO‐infected mice actually have higher inflammatory gene expression than those from WT‐infected mice despite having a milder tissue‐level phenotype. Finally, this difference is augmented by 72 h post infection when the phenotype is the most severe, particularly in the WT‐infected mice (Fig 3E, Appendix Fig S3G and H, and Fig 1 and Appendix Table S2). These results indicate that both strains induce an *Il‐1*‐family inflammatory response in neutrophils, but at the time of severe phenotype, the response is lower in the cells from WT‐infected than β‐h/c‐KO‐infected mice.

### KO GBS‐infected maternal, but not fetal, macrophages express greater inflammation than WT‐infected at the time of severe phenotype

**Figure 4:**
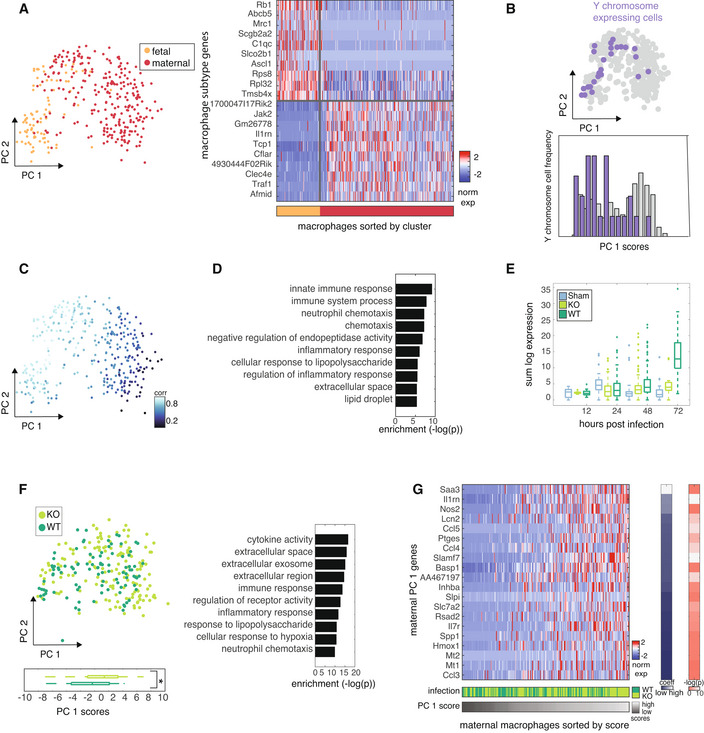
Maternal but not fetal macrophages recapitulate inflammatory response dynamics PCA on macrophages from 72 h post infection colored by hierarchical clustering (left). Z‐scored expression of differentially expressed and upregulated genes between clusters (right).PCA on macrophages colored by cells that express Y chromosome genes (top). Distribution of PC 1 scores between cells that do and do not express Y chromosome genes (bottom).PCA on macrophages from 72 h post infection colored by correlation with fetal expression profile.GO enrichment of the top 46 WT‐induced genes.Boxplots of PC 1 scores between maternal macrophages from KO‐ and WT‐infected mice. Central band of the boxplot shows the median, boxes represent the IQR, and whiskers represent the most extreme values not considered outliers. Significance was determined by paired *t*‐test (12 h: NS, 24 h: NS, 48 h: NS, 72 h: *P < 10^−04^). The numbers of cells (biological replicates) included in the boxplots (Sham, KO, WT) are 15, 9, 30 (12H); 24, 49, 105 (24H); 61, 84, 179 (48H); 39, 16, 31 (72H).PCA on maternal macrophages colored by condition with boxplot of PC 1 score distributions in each condition below. Central band of the boxplot shows the median, boxes represent the IQR, and whiskers represent the most extreme values not considered outliers. Significance was determined by paired *t*‐test (*P < 10^−03^). The numbers of cells (biological replicates) included in the boxplots (KO, WT) are 152, 199.Z‐scored expression of genes contributing to high PC 1 scores (right). Maternal macrophages from 72 h post infection are ordered by PC 1 score (bottom bar) and infection condition is indicated in middle bar. Coefficient of each gene is indicated in the bar on the right, and −log(*P*) of expression difference of that gene between KO‐ and WT‐infected cells determined by paired *t*‐test is indicated in the rightmost bar.

Given the observed neutrophil response dynamics between conditions, we asked if the dynamics in macrophages followed the same trend. Because the inflammatory difference in neutrophils was most pronounced at 72 h post infection (*P* < 10^−84^), which was also the time phenotype was most severe, we defined the peak macrophage response at that same time point. Hierarchical clustering defined two subgroups of cells which corresponded to PC 1‐high and ‐low cells respectively (Fig 4A). Differentially upregulated genes between these clusters suggested that they correspond to macrophages of maternal and fetal origin (Fig 4A). To validate the fetal origin of the PC 1 low cluster, we leveraged the fact that cells expressing Y‐chromosome genes cannot be of maternal origin and measured expression of Y‐chromosome genes in macrophages. For placentas from male fetuses, we used Y‐chromosome gene expression to infer fetal identity and confirmed that they are overrepresented in the PC 1 low cluster (Fig 4B and Appendix Fig S4A). To further validate that this cluster represents fetal macrophages from both male and female fetuses, we measured the correlation between each macrophage and the fetal macrophage expression profile (derived from the unambiguously annotated macrophages in males) and found higher correlations in the PC 1 low cluster (Fig 4C, see Materials and Methods). Cell cycle does not contribute to the major source of variance captured by PCA (Appendix Fig S4B).

Having distinguished this population of macrophages of fetal origin, we asked whether these were affected by GBS infection since they have an important role in mediating placental functions (Erlebacher, 2013; Mezouar *et al*, 2021). We asked which genes were differentially expressed and upregulated between fetal macrophages from sham‐infected mice or mice infected by β‐h/c KO or WT GBS at each time point post infection. We found that fetal macrophages from WT‐infected placentas at the 72 h time point are the only cells that exhibit inflammatory activation, based on differentially expressed genes between conditions and comparison of expression of fetal inflammatory genes, derived from differentially expressed genes, across time points (Fig 4D and E, and Appendix Fig S4C).

We next asked if there is an overall difference in the inflammatory response of maternal macrophages between conditions at 72 h post infection. Sham macrophages were removed from analysis because they were relatively rare within the maternal macrophage cluster. We performed PCA on remaining maternal macrophages and compared the PC 1 score distribution of the maternal macrophages from β‐h/c‐KO‐ and WT‐infected mice. As we observed in analysis of neutrophils from this dataset, we found that macrophages from β‐h/c‐KO‐infected mice had significantly higher PC 1 scores than those from WT‐infected mice (Fig 4F). GO enrichment analysis confirmed a functional inflammatory enrichment in the genes that contribute to high PC 1 scores, supporting the inflammatory expression in these cells (Fig 4G, and Appendix Fig S4D and Appendix Table S2). Again, measurement of differentially expressed genes between conditions confirmed these findings (Appendix Fig S4E and F).

To compare the dynamics between conditions across time following infection, we measured PC 1 scores across maternal macrophages defined independently at each time point (see Materials and Methods). Consistent with the trend observed in neutrophils, we found that WT‐infected macrophages exhibited greater inflammatory expression than β‐h/c KO‐infected until 48 h post infection. By 72 h post infection, however, the KO‐infected macrophages had higher inflammatory expression than WT‐infected (Appendix Fig S4G).

These results reveal common inflammatory dynamics in maternal macrophages and neutrophils. Cells from WT‐infected placentas show greater inflammatory expression relative to those from KO‐infected placentas in the first stage post infection, and then at the time when severe phenotype presents (48 h post infection), cells from KO‐infected mice show greater inflammatory expression than WT‐infected. Conversely, fetal macrophages do not show inflammatory expression until the point of severe phenotype, at which point inflammatory expression is induced only in WT‐infected fetal macrophages.

### Human placental macrophage responses to GBS recapitulate *in vivo* toxin‐dependent differences

**Figure 5:**
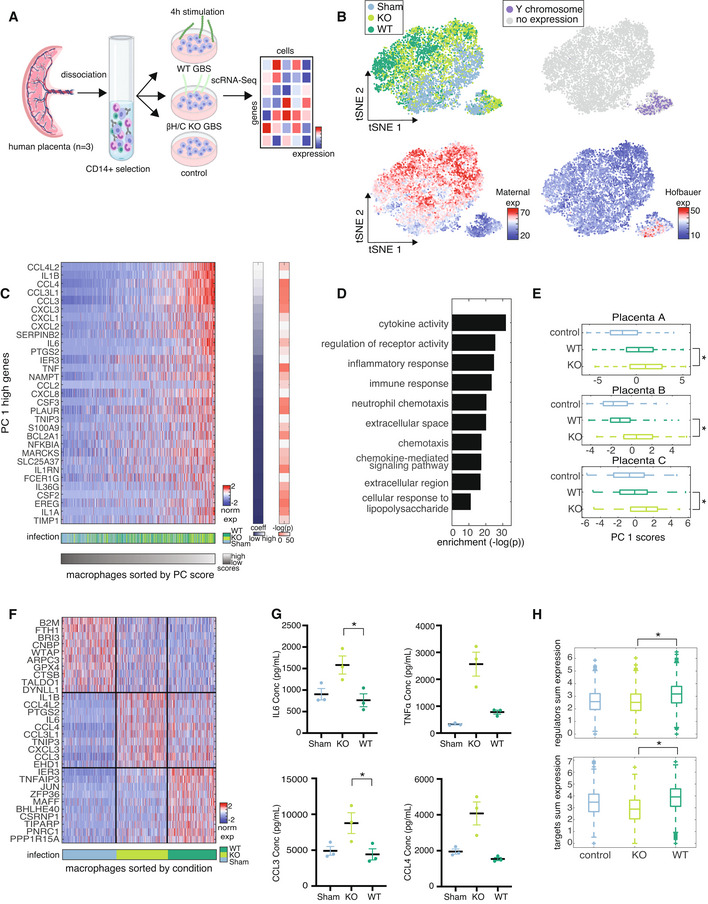
Human placental macrophage responses to GBS recapitulate *in vivo* toxin‐dependent differences Schematic of the experimental approach. Cells were isolated from human placentas (*n* = 3) and macrophages were selected by CD14 expression. Macrophages were cultured overnight and stimulated with GBS. After 1 h extracellular GBS was killed and cells were cultured for an additional 3 h. Following stimulation cells were collected and analyzed by scRNA‐Seq.tSNE of macrophages from placenta A colored by: treatment condition (top left), Y chromosome gene expression (top right), sum expression of marker genes for macrophages of maternal origin (bottom left), Hofbauer cells (bottom right).Z‐scored expression of PC 1 high genes with corresponding infection condition in placenta A. PC 1 score and condition are indicated in the bars below, and gene coefficient as well as −log(*P* value) of the expression between KO‐ and WT‐infected cells are indicated in the bars on the right.GO enrichment for the top PC 1 genes in maternal macrophages from placenta A.Boxplots of PC 1 scores between conditions on maternal macrophages from each condition per replicate. Central band of the boxplot shows the median, boxes represent the IQR, and whiskers represent the most extreme values not considered outliers. Significance was determined by paired *t*‐test between β‐h/c KO and WT GBS‐infected conditions (below, Rep A: *P < 10^−13^, Rep B: *P < 10^−50^, Rep C: *P < 10^−19^). The numbers of cells (biological replicates) included in the boxplots (control, WT, KO) are 1810, 1759, 1742 (Placenta A); 1765, 476, 334 (Placenta B); and 557, 552, 782 (Placenta C).Z‐scored expression of top differentially expressed and upregulated genes between maternal macrophages from placenta A in each infection condition by rank sum test (*P* < 0.0001).Luminex immunoassay of IL6, TNFɑ, MIP1ɑ and MIP1β levels on supernatants from human placental macrophage cultures. Each point is the average of two technical replicates, each the average of two plate replicates, with exceptions noted in the methods. Bars indicate mean + SEM between biological replicates in each condition. Paired *t*‐test was performed across KO‐ and WT‐infected conditions to determine significance (TNFɑ, *P < 0.07, CCL3, *P < 0.048).Sum expression of upstream regulators (top) and downstream targets (bottom) of *BHLHE40* in maternal macrophages across conditions. Central band of the boxplot shows the median, boxes represent the IQR, and whiskers represent the most extreme values not considered outliers. Significance was determined by paired *t*‐test between β‐h/c KO and WT GBS‐infected conditions (regulators: *P < 10^−74^, targets: *P < 10^−133^). The numbers of cells (biological replicates) included in the boxplots (control, WT, KO) are 1810, 1759, 1742.

The relative reduction in WT‐induced inflammation at the time of severe phenotype may either have resulted from direct suppression by β‐h/c or from another signal in this complex physiologic setting, such as a wound healing response to cells lysed by β‐h/c. In order to test whether β‐h/c can directly modulate responding immune cells, we stimulated human placental macrophages *ex vivo* with β‐h/c KO or WT GBS and measured their transcriptomic responses to infection. Cells were isolated from three individual full‐term human placentas from GBS‐negative women: one with a male fetus (Replicate A) and two with female fetuses (Replicates B and C, see Materials and Methods). Macrophages were selected from each placenta by density gradient centrifugation and expression of CD14, cultured overnight and stimulated with KO or WT GBS or PBS control. After 1 h, antibiotics were added to kill extracellular bacteria and cells were incubated for an additional 3 h, after which they were collected and processed for scRNA‐Seq (Fig 5A). Contaminating stromal cells were removed from analysis by sequential filtering based on the expression of marker genes to select for the macrophages (Appendix Fig S5A–C and Appendix Table S3, see Materials and Methods).

In each sample, visualization of macrophages with tSNE revealed two clusters, corresponding to placental macrophages of maternal and fetal origin (Hofbauer cells), based on expression of previously annotated marker genes (Thomas *et al*, 2021; Fig 5B and Appendix Fig S5D). As with mouse placental macrophages, we measured expression of Y chromosome genes in human placental macrophages from Replicate A, which had a male fetus. We found expression of Y chromosome genes in the cells that express Hofbauer marker genes, validating the fetal origin of these cells (Fig 5B). This gene set was used to identify macrophages of fetal origin in replicates B and C, which had female fetuses. We removed from analysis a number of cells (209 out of 5,520) that clustered with maternal macrophages and were positive for Y chromosome gene expression, but their presence did not affect the results of the analysis (Appendix Fig S5E). Because most of the responding macrophages captured in our *in vivo* data were maternal macrophages, we focused our analysis on the response of the equivalent human placental population.

To test for a difference in response between cells from KO‐ and WT‐infected mice as we observed *in vivo*, we performed PC score analysis on maternal macrophages. The genes that contribute to high PC 1 scores and the GO analysis of those genes revealed an inflammatory program in responding macrophages (Fig 5C and D, and Appendix Fig S5F–H and Appendix Table S2). Comparing between conditions, we found that PC 1 scores (a proxy for inflammatory expression) from β‐h/c KO‐infected macrophages were significantly higher than those from WT‐infected macrophages (paired *t*‐test, *P* < 10^−13^, Fig 5E). Analysis in placentas B and C also revealed significantly higher PC 1 scores in KO‐ than WT‐infected maternal macrophages (paired *t*‐test, Rep B: *P* < 10^−50^, Rep C: *P* < 10^−19^, Fig 5E).

The relative reduction in inflammatory response in the WT relative to the KO condition led to our hypothesis that the presence of β‐h/c suppresses this response in the maternal intrauterine compartment. To investigate this possibility, we queried for genes that are differentially expressed and upregulated in response to each infection condition. Consistent with the results of PC score analysis, KO‐infected macrophages were significantly enriched in *IL‐1*‐inflammatory response genes (Fig 5F). We validated higher expression of a subset of these genes with protein‐level comparison between conditions (see Materials and Methods). We found that IL6 and CCL3 were significantly higher in KO‐ than WT‐infected cells (*P* < 0.02 and *P* < 0.048 respectively), and TNFɑ, CCL4, CXCL1 and CCL2 trended higher in KO‐ than WT‐infected cells. Il1ɑ levels were elevated in WT‐ relative to KO‐infected (NS), which may reflect post translational processing of IL1ɑ (Rubartelli *et al*, 1993; Fig 5G and Appendix Fig S5I).

In contrast, the genes significantly enriched in response to WT GBS include several genes implicated in inflammatory regulation in other physiologic contexts, suggesting that after an initial inflammatory response upon recognition of GBS, β‐h/c could actively suppress this inflammation through induction of suppressive and regulatory genes (Fig 5F). Because *BHLHE40* is a transcription factor, we measured expression of its upstream regulators and downstream targets in these cells and found that those were also significantly upregulated in the WT condition, providing additional evidence for β‐h/c inducing inflammatory regulation, which, in turn, could allow insufficient bacterial control and lead to worse phenotypes (Fig 5H).

## Discussion

In this work, we defined the placental cell state dynamics in response to GBS with or without expression of the toxin β‐h/c. We first recapitulated the difference in tissue‐level phenotype between the response to a β‐h/c KO and isogenic WT GBS strain (Randis *et al*, 2014), using a mouse model of ascending infection. We found that WT GBS induced a more severe phenotype measured by histological analysis and bacterial burden than the β‐h/c KO (consistent with prior studies). We analyzed corresponding scRNA‐Seq data to understand the cell state changes that accompanied this phenotypic progression. PC score analysis revealed GBS induction of an *IL‐1* family inflammatory response in innate immune cell populations, but different response dynamics depending on the presence of β‐h/c. In the first stage after infection (up to 48 h), immune cells from WT‐infected mice expressed relatively higher inflammatory response genes than those from β‐h/c KO‐infected mice. In the later stage however, (48–72 h), we observed relatively less inflammatory expression in cells from the WT‐ compared to the β‐h/c KO‐infected mice, concurrent with the presentation of severe phenotype in WT‐infected mice. We found that fetal macrophages were only activated when infected with WT GBS at 72 h post infection. These data reveal a largely conserved response between conditions, but our systematic study design and use of scRNA‐Seq data as a primary readout reveals subtle shifts in response dynamics that can provide important insight into the tissue‐level responses between conditions. While the scRNA‐Seq differences are subtle, we validated the relatively lower expression of inflammatory response genes induced by WT‐compared to β‐h/c KO‐GBS in maternal macrophages and neutrophils in an *ex vivo* infection of human placental macrophages, where we found a robust and reproducible difference between conditions.

**Figure 6:**
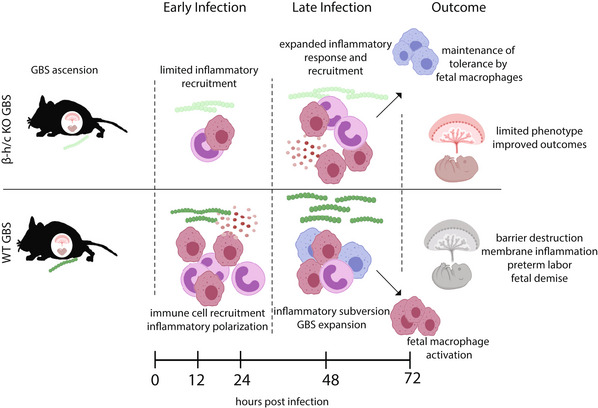
A model for GBS infection in the placenta leading to severe outcomes

These data led us to propose a model for GBS infection (Fig 6) in the placenta leading to severe outcomes. Upon infection with β‐h/c KO GBS, the inflammatory response, mediated primarily by maternal macrophages and neutrophils, increases with time post infection and is sufficient to limit bacterial growth, prevent activation of fetal macrophages and maintain membrane barrier function. Conversely, infection with WT GBS initially elicits a robust inflammatory response from innate cells, which may aid GBS invasion of placental membranes. However, at a certain point after infection, β‐h/c begins to exert a suppressive effect on host cells, which results in the relatively lower inflammatory expression when compared to cells from KO‐infected mice. This subversion leads to insufficient bacterial control, disruption of immunological and physical barriers and ultimately leads to more severe phenotypic outcomes.

β‐h/c can have different effects on host immune cells depending on concentration of bacteria and site of infection (Okumura & Nizet, 2014). Previous work has shown that β‐h/c induces expression of *IL‐1*‐family inflammatory cytokines through NLRP3 activation, leading to neutrophil recruitment and macrophage pyroptosis (Okumura & Nizet, 2014; Whidbey *et al*, 2015; Leclercq *et al*, 2016). Others have reported that β‐h/c is necessary for inflammasome activation (Okumura & Nizet, 2014), and while we have shown that β‐h/c‐KO GBS induced *IL‐1*‐family inflammatory gene expression, further work is required to see whether this expression is reflected in cytokine secretion. This activation could result from other pathogen sensing mechanisms, like pattern recognition receptor (PRR) recognition of pathogen‐associated molecular patterns (PAMPs) on GBS, and it could also change based on context differences like infection site or bacterial load.

Depending on environmental factors, inflammatory activation in the host can either help or hurt the survival of GBS (Landwehr‐Kenzel & Henneke, 2014). We have shown that over the course of infection, the WT GBS induced relatively more inflammation than β‐h/c‐KO GBS in the initial response, perhaps aiding its own invasion to and survival in this new niche. Our work introduces a new potential route of evasion for GBS through subversion of the very response that it elicits in the later stage of infection. The relative reduction in inflammatory response which is concurrent with expression of immunoregulatory genes in WT‐infected host cells, leads us to propose that β‐h/c subversion of the inflammatory response allows increased bacterial proliferation leading to barrier breach and adverse outcomes. An important caveat to consider in interpreting our *ex vivo* validation data is the way bacterial levels in culture impact inflammatory gene expression and protein levels, though we do not expect a significant difference in growth rates in this short‐term assay. It will be interesting to consider how factors such as time post infection, bacterial burden or other environmental factors impact inflammatory subversion by GBS.

One of the most important functions of β‐h/c in an infection setting is cytolysis of host cells and hemolysis for acquisition of heme, which promotes bacterial survival (Whidbey *et al*, 2013). The placenta is involved in maturation of primitive erythroid cells and is therefore a permissive site for GBS hemolysis and virulence (Van Handel *et al*, 2010). We addressed the possibility that the relative reduction in inflammatory response in the later time points that we observed could have resulted from a wound healing phenotype in response to the massive amount of cell lysis in the environment. We showed through our *ex vivo* analysis that even in the absence of any tissue context, the presence of β‐h/c elicits a weaker inflammatory response than in a β‐h/c KO isogenic control. We also considered the possibility that cell lysis *in vivo* contributes to this suppression. Undoubtedly, this function of β‐h/c contributes to barrier breach that allows colonization of GBS along the membranes. It may also explain the competing effects of β‐h/c *in vivo* unfolding at different times post infection as the environment is changing.

The balance of macrophage polarization states in the placenta is essential for successful pregnancy (Erlebacher, 2013; Mezouar *et al*, 2021). Previous work has implicated an inflammatory placental macrophage skew with different pathological outcomes of pregnancy (Erlebacher, 2013; Yao *et al*, 2019). We observed, in contrast, that severe phenotype was correlated with a reduction in placental macrophage inflammation in WT‐infected mice.

We consider a number of ways to explain this apparent contradiction: First, we distinguish between fetal and maternal macrophage inflammation in the placenta. While a reduction in maternal macrophage inflammation was correlated with worse outcomes, we also observed a concurrent activation of fetal macrophages, which, we suggest, could result from GBS overwhelming the maternal response. Given the colonization of GBS along fetal membranes starting at 12 h following infection (Fig 1C and D), it is unlikely that anatomical compartmentalization prevents activation of the fetal macrophages until 72 h post infection. Instead, it is more likely that the functional response of maternal immune cells eliminates the need for activation of fetal immune cells in the placenta, which in turn allows continued normal function of fetal immune cells. Increased tissue damage can also play a role, but if anything that might elicit a hypoxia‐dependent wound healing response, which can contribute to inflammatory regulation. This activation could prevent fetal macrophages from executing normal functions important for fetal support and, together with barrier disruption, interfere with success of the pregnancy. Aberrant inflammatory skew can induce premature labor by mimicking the inflammatory milieu that triggers labor under normal circumstances (Erlebacher, 2013; Yao *et al*, 2019). We suggest that inflammatory skew of fetal macrophages can lead to this phenotype. We do not see this difference in response in human placental fetal macrophages *ex vivo* because tissue context is critically important in maintaining a tissue‐resident macrophage phenotype.

Another possible explanation for this discrepancy is that the effect of macrophage inflammatory subversion or activation depends precisely on the stage of pregnancy (Vento‐Tormo *et al*, 2018; Goldstein *et al*, 2020). While the balance of macrophage polarization states is important throughout pregnancy, the baseline inflammatory state of the placenta changes with time (Mor & Cardenas, 2010). The dynamics we observed can change based on the pregnancy stage and may have implications for earlier testing and intervention in human pregnancy.

The spatial compartmentalization of the placenta is also very important for barrier function. Previous work has suggested that GBS ascends through the cervix and breaches placental layers in order to reach the amniotic fluid and fetus (Whidbey *et al*, 2013), suggesting that physical barrier function is important for fetal protection against GBS. Our time course data suggests that GBS is present along the fetal membrane as early as it has ascended to the placenta (Fig 1). Therefore, the robust immunological barrier function of the maternal innate immune response must be sufficient to limit activation of fetal macrophages even though GBS is in close proximity to these cells. Future work would elucidate the precise relationship between spatial compartmentalization in the placenta and its relationship to cell state changes in response to GBS. Spatial analysis would also enhance our understanding of the effect of infection on other functional processes in the placenta, including the roles of different trophoblast subtypes in placental function.

In considering the implications of our findings, it is important to note the limitations of this ascending model of GBS infection in pregnant mice. Mouse and human pregnancy differ in duration, placental structure and development, and nutrient exchange and immune mechanisms at the maternal‐fetal interface (Ander *et al*, 2019; Hemberger *et al*, 2020; Megli & Coyne, 2022). While an advantage of this model is that it has been shown to recapitulate many of the features of clinical disease, it is important to consider these differences in interpreting results, particularly as they relate to inflammatory activation in different immunologic compartments in the placenta. As discussed earlier, the baseline inflammatory activation in the placenta changes with each stage of human pregnancy (Mor & Cardenas, 2010; Vento‐Tormo *et al*, 2018; Goldstein *et al*, 2020). Our *in vivo* data capture the response to infection in the middle of mouse pregnancy, a time point that corresponds roughly to the second trimester in humans. While we are able to validate our crucial findings in macrophages from human term placentas, suggesting that the changes in response induced by the presence of β‐h/c are robust to species, time point in pregnancy and infection duration, it is important to consider that the effect of this interaction on the other cells present and on normal placental function may be different in an infected human, and depending on the point in pregnancy, the inflammatory milieu and the functional demands of the fetus at that stage. It is also important to consider the way our technical approach may skew the representation of certain cell populations, such as the syncytiotrophoblasts, which are too large to be captured by our single cell method, thus limiting our ability to interpret these results in the context of the whole tissue environment. Future work is required to map other cell types and their cell state changes in response to infection in order to compare results meaningfully between different contexts. Finally, additional efforts will also be required to refine our classification of macrophage subtypes in human term placentas. Our cell subtype identification method suggested the presence of macrophages of maternal origin in the placenta even though it had been separated from the maternal decidua (see Materials and Methods). This highlights the need to refine existing cell type definitions in light of new data, which may elucidate the behavior of different cell subtypes under different pathological conditions.

Our work has highlighted a potential route of GBS disease modulation based on WT GBS‐induced gene expression (Fig 5). Some of the upregulated genes in response to WT GBS infection in maternal macrophages have functions in inflammatory regulation, including *IER3*, *TNFAIP3*, and *BHLHE40* (Coornaert *et al*, 2009; Arlt & Schäfer, 2011; Parvatiyar & Harhaj, 2011; Cook *et al*, 2020). *BHLHE40* is a transcription factor involved in different cellular processes in a variety of tissues including circadian rhythm regulation and differentiation (Boudjelal *et al*, 1997; Honma *et al*, 2002; Shen *et al*, 2002; Yun *et al*, 2002). Recent work has implicated this gene in inflammatory regulation in different physiologic contexts (Jarjour *et al*, 2019; Cook *et al*, 2020), and we hypothesize that because of its regulatory role in inflammation, targeting *BHLHE40* could limit host inflammatory suppression leading ultimately to better phenotypic outcomes. Consistent with our *in vivo* findings, both the KO‐infected and WT‐infected conditions show significant inflammatory activation, which is reasonable given that the conserved innate response has evolved to respond to diverse stimuli and the presence or absence of a single toxin should not impact the overall response. Our *ex vivo* analysis, however, is designed in a way that can provide some insight into the impact of the presence of β‐h/c. It could be the case that these regulatory genes are induced by a stress response caused by increased greater cell lysis due to β‐h/c, in which case regulation would be an indirect consequence of β‐h/c, but which allows increased colonization and survival for WT GBS. This study lays the groundwork for future investigation into immune regulation better in this setting and will address precisely how this regulation impacts phenotype in an *in vivo* context. Ultimately, this has important implications for clinical intervention through modulation of this and other inflammatory regulators for improved bacterial control, while limiting the impact on normal host cell function during pregnancy.

## Materials and Methods

### Bacterial strains and growth conditions

GBS wild type (WT) strain CNCTC 10/84 (1169‐NT1; ATCC 49447, serotype V) and the isogenic, β‐h/c‐deficient, in‐frame *cylE*Δcat mutant (referred to as β‐h/c KO) were used. The WT CNCTC 10/84 strain is hyperhemolytic in comparison to other GBS strains. The *cylE* KO strain (β‐h/c KO) is nonhemolytic, lacks production of the granadaene pigment, and is in the CNCTC 10/84 genetic background. All bacteria were grown at 37°C in trypticase soy (TS) broth and plated on TS agar.

### Ascending GBS infection

All experimental procedures were reviewed and approved by the New York University Institutional Animal Care and Use Committee. Timed‐pregnant C57BL6/J mice were purchased from Jackson Laboratories (Bar Harbor, Maine) and given 3 days to acclimate to new surroundings prior to experimental procedures. Overnight GBS cultures were centrifuged and resuspended in a 1:1 mixture of PBS and sterile 10% gelatin. On pregnancy day 13 (E13), dams were anesthetized with isoflurane, and 10^7^ colony forming units (CFU) of WT GBS or cylE KO (50 μl total volume) was administered intravaginally using a sterile pipette as previously described (Randis *et al*, 2014). A sham‐infected group was similarly inoculated with a 1:1 mixture of PBS and sterile 10% gelatin. Upon recovery from anesthesia, animals were housed in separate cages and monitored daily for the remainder of the experimental procedures to document general wellness, and preterm delivery. Pregnant animals were euthanized in cohorts on E13.5, E14, E15, and E16 to measure longitudinal changes. Vaginal swabs were collected prior euthanasia using a sterile, calcium alginate‐tipped swab that was vigorously shaken into 300 μl of PBS. Serial dilutions were plated for determination of GBS CFUs on CHROMagar™ StrepB plates. A laparotomy was performed under sterile conditions for gross and histopathological inspection of placentas and fetuses. Any intrauterine fetal demise as noted on uterine inspection was compared between groups, and was observed in WT‐infected samples as previously described (Randis *et al*, 2014). Placental tissue (a single placenta from each litter, most proximal to cervix, left side) was homogenized and plated on chromogenic agar to assess for bacterial invasion. One whole placenta from each mouse (second most proximal to cervix, left side) was fixed in 4% PFA for histopathological analysis. Two placentas from each mouse (two most proximal to cervix, right side, when possible) were set aside for scRNA‐Seq processing.

### Microscopy and staining

Pregnant animals were anesthetized and euthanized at the set time points post inoculation and an entire fetal‐placental unit (second most proximal to cervix, left side) was removed, separated, and fixed in 4% paraformaldehyde. The NYU Langone's Experimental Pathology/Immunohistochemistry Core Laboratory embedded all fixed tissues in paraffin and then serially sectioned on to slides. Hematoxylin and eosin staining was performed as per standard protocols. For macrophage detection, dams with confirmed GBS colonization on chromogenic agar and sham controls were selected. 4 μm sections were collected on Plus slides (Fisher Scientific), stored at room temperature, and incubated for 1 h at 60°C immediately prior to staining. Unconjugated rabbit anti‐mouse F4/80, clone D2S9R, (Cell Signaling Technologies Cat# 70076 Lot# 1A11‐01) was used for chromogenic immunohistochemistry performed on a Ventana Medical Systems Discovery XT platform with online deparaffinization using Ventana reagents and detection kits. Samples were pretreated using Cell Conditioner 1 (Tris‐Borate‐EDTA pH 8.5) for 20 min. Endogenous peroxidase activity was blocked for all samples. F4/80 was diluted in antibody diluent (Cell Signaling Technologies, Catalog # 8112) 1:100 and incubated for 3 h 37°C. F4/80 was detected with goat anti‐rabbit horseradish peroxidase‐conjugated multimer incubated for 16 min. The complex was visualized with 3, 3 diaminobenzidene and enhanced with copper sulfate. Slides were washed in distilled water, counterstained with hematoxylin, dehydrated and mounted. Negative controls substituted antibody diluent for primary antibody. Slides were scanned at 40× magnification using the Leica SCN400 whole slide scanner. Immunofluorescent labeling of GBS was performed following deparaffinization and rehydration of sectioned tissue. Heat‐induced epitope retrieval was performed as per manufacturer's recommendations (Abcam). Nonspecific binding sites were blocked with 10% normal goat serum and 1% bovine serum albumin (Sigma). Rabbit anti‐GBS polyclonal antibody (Abcam ab53584, 1:200 dilution) was applied. Following serial washes with phosphate‐buffered saline (PBS) + 0.025% Triton X‐100, Alexa Fluor 647 goat anti‐rabbit immunoglobulin G (IgG; Invitrogen; 1:500 dilution) was added for 30 min in the dark with gentle shaking. Slides were counterstained using Hoechst 33342 (Invitrogen). Coverslips were mounted with Vectashield Hardset mounting medium (Vector Laboratories), and slides were stored at 4°C. Slides to which no primary antibody or no secondary antibody was added served as negative controls. Slides were scanned at 20× resolution using the Hamamatsu NanoZoomer 2.0HT scanner.

### Single cell and library preparation for scRNA‐Seq

Two placentas from each mouse were washed in PBS and bisected. A small piece was homogenized, serially diluted, and plated to confirm GBS colonization of that tissue. The remaining tissue was minced and digested at 37°C for 20′ in DMEM + CaCl_2_ (0.005%) + Collagenase type IV (20 mg/ml). Red blood cell lysis was performed in ACK Lysing Buffer for 5′. Remaining debris and red blood cells were removed using a lymphocyte separation gradient according to manufacturer instructions. We note that the cell isolation method and other technical factors may impact the array of cell types recovered and defined the observed cell types based on genes expressed in those cells. Cells were counted and viability was assessed by trypan blue on a hemocytometer. Two placentas from each mouse were hashed with Biolegend TotalSeq™–A antibodies using standard protocol. Cells were run on the 10× Genomics Chromium Controller with the Single Cell 3′ v3 system. cDNA amplification and library preparation of the mRNA was processed according to 10× Genomics Single Cell 3′ v3 manufacturer's instructions. For the hashtag oligos, 1ul of HTO PCR additive primer was added to the 10× cDNA amplification step, and the supernatant from the 0.6× cDNA cleanup was kept and processed according to the Cell Hashing protocol, with 14 PCR cycles and a 1.2× cleanup after the PCR.

### Sequencing

Paired‐end sequencing was performed on a Next‐Seq or Nova‐Seq, with read format R1‐28 i‐8 R2‐91 or R1‐26 i‐8 R2‐98, respectively. Hashtag libraries were sequenced on the same flow‐cells at 1/10 proportion of the mRNA.

### Single cell processing and filtering for *in vivo* data

Cell Ranger pipeline (V.3.0.0) was used for demultiplexing, mapping to the mouse genome (mm10) and counting unique molecular identifiers (UMIs) for individual cells. Cells were filtered out if they had less than 500 UMIs, and if more than 20 or 15% of their transcripts were ribosomal or mitochondrial genes, respectively. We normalized the expression to transcripts per median (TPM) and log transformed the expression. Hashed placentas were not deconvolved for analysis but the two adjacent placentas used for hashing were combined for analysis. For all cells from sham‐ and KO‐infected mice included in analysis, cells come from two placentas from a single mouse from each time point, and for cells from WT‐infected mice, they come from two or four placentas from either one or two mice depending on the colonization results summarized in Appendix Table S1.

### Lineage and cell type identification

Lineage clusters were generated through hierarchical clustering and identified primarily through expression of marker genes independently from the literature. Data from each time point was clustered independently, and while the three main lineages separated for most time points, at 48 h post infection, granulocytes were so inflammatory that they separated out separately for macrophages. In order to maintain consistent cell clustering across time points for comparison, four clusters were isolated from cells at 48 h post infection, and the neutrophil and macrophage clusters were combined to form the immune cell lineage. Cluster identity was confirmed based on expression of manually curated genes (Appendix Fig S2B) and differentially expressed genes identified by Ranksum test (*P* < 0.0001) between clusters (Fig 2B). Within the immune and trophoblast lineages, cells were sub clustered and identities were assigned based on expression of manually curated genes from the literature unique to each subcluster (Appendix Fig S2D and F). For samples from time points with varied cell composition, immune lineage cluster cells were subjected to additional rounds of clustering in order to isolate clusters of inferred neutrophils and macrophages on the basis of lineage marker genes referred to in Appendix Fig S2.

### Cell state analysis

Data from each time point were analyzed separately and major cell lineages, cell types and subtypes were identified separately in the data from each time point. Immune cell subpopulations were isolated for analysis by hierarchical clustering. For each immune cell population, subpopulations were isolated, as relevant, by additional clustering. PCA was then performed on log transformed data using informative genes which were defined as highly expressed and variable genes (fano‐factor and mean expression). The genes *Hbb‐bs*, *Hbb‐bt*, *Hba‐a1* and *Hba‐a2* were excluded from the Figure 4 macrophage analysis as we interpreted their over expression in these cells as reflecting non‐cellular expression from lysed erythrocytes. For each analysis, genes that contribute to the relevant PC (1 or 2 depending on the analysis) were identified as genes with the highest coefficients for that PC. Gene Ontology (GO) enrichment confirmed functional characterization of these sets of genes. PC 1 scores were compared between cells of different conditions by paired *t*‐test to determine whether condition explained the major source of variance in each subpopulation of cells. In order to compare PC scores across time points, equivalent cell populations were isolated from the data in each time point, and then scores from the data in each time point were calculated based on mu and coefficients defined in the reference PCA.

### Cell subtype enrichment analysis

Trophoblast lineage subtypes were defined by hierarchical clustering. Within each subtype differentially expressed genes were computed between cells from sham‐ and GBS‐infected mice. GO enrichment analysis was performed on genes expressed significantly more highly in GBS‐infected relative to sham‐infected cells and the −log(*P*) of all cell subtypes in the top enrichment categories from all subtypes are displayed. Endodermal *Afp* high cells were excluded from analysis due to lack of differentially expressed genes between cells from sham‐ and GBS‐infected mice.

### Cell cycle score analysis

Cell cycle score was measured by computing the log of the ratio between sum log expression of G1_S genes per cell divided by sum log expression of G2 genes per cell. G1S and G2 genes are delineated in Appendix Table S4.

### PC score analysis across time points

Neutrophil and macrophage subclusters were identified within immune lineage clusters for each time point independently. For each PC score analysis at different time points, PCA was performed on the reference time point data to determine mu. To measure equivalent PC scores at different time points, coefficients were calculated within each population based on the same set of informative genes and mu from the reference time point PCA.

### Fetal macrophage identification analysis

Macrophages from male fetuses were identified on the basis of sum log Y chromosome expression above a threshold (0 in mouse and 0.4 in human). For mouse data, differentially expressed genes were defined between male fetal macrophages and all other cells, and any significant genes were used for hierarchical clustering of all cells into two groups. The group with an overrepresentation of male fetal macrophages was defined as fetal. To measure fetal correlation as a confirmation of identity of non‐Y chromosome‐expressing macrophages assigned to the fetal cluster, expression of each cell was compared with the average expression of the same set of differentially expressed genes across all confirmed male fetal macrophages. This analysis was repeated independently on all macrophages included in analysis (from Sham‐ KO‐ and WT‐infected conditions) at each time point to identify a fetal expression profile unique to that time point. Because analysis at each time point included cells processed from either 6 or 8 placentas, each fetal analysis included some cells from a male fetus to provide a ground truth at that time point. For human data, clusters were defined by hierarchical clustering on informative genes, defined as highly expressed and variable genes (fano‐factor and mean expression) and the group with an overrepresentation of male fetal macrophages was defined as fetal.

### Human subjects

Human placental samples were provided through the Cooperative Human Tissue Network funded by the National Cancer Institute. All tissues were collected in accordance with Vanderbilt University Institutional Review Board (approval #131607, 181998) and the Declaration of Helsinki. Signed informed consent was obtained when pregnant person arrived for scheduled C‐section prior to delivery and sample collection. Placentas were obtained at the time of cesarean section from pregnant patients who were term, non‐laboring, healthy donors with no significant medical conditions between the ages of 18–40 years. Samples were deidentified prior to research use precluding the use of maternal or neonatal covariates.

### Placental macrophage isolation

The villous core of human placentas were isolated as previously described (Tetz *et al*, 2015). Placental cotyledon tissue was separated from maternal decidua basalis prior to digestion and washed in PBS to remove blood and any clots. The tissue is minced well and placed into 250 ml sterile bottles with sterile digestion solution containing 150 mg/ml deoxyribonuclease, 1 mg/ml collagenase, and 1 mg/ml hyaluronidase at 10 ml/g of tissue. Placenta was enzymatically digested at 37°C shaking at 180 rpm for 1 h. Cells were filtered from undigested tissue using a 280 mm metal sieve followed by 180 and 80 mm nylon screens. After centrifugation at 450 *g*, cells were resuspended in a cold 25% Percoll solution (Percoll: RPMI) and overlaid onto 50% Percoll with 2 ml of 1× PBS on the top of the density gradient. This was followed by a 40 min spin without a break. After subsequent red blood cell lysis, CD14^+^ macrophages were positively selected using the magnetic MAC® large cell separation column system according to the manufacturer's instructions. These cells were plated in a 12‐well dish at 2 × 10^6^ cells/well and rested overnight in RPMI with 10% FBS and 1% antibiotic‐antimycotic media at 37°C with 5% CO_2_ before experimentation.

### *Ex vivo* placental macrophage infection

The next morning, macrophages were washed with RPMI with 10% FBS without antibiotics. GBS mutant strain ΔcylE and parent strain 10/84 were added at an MOI of 0.5 for 1 h prior to adding 2× antibiotic‐antimycotic. After an additional 3 h of incubation, cells were then collected with gentle scraping in cold calcium‐free and magnesium‐free PBS. After centrifugation 300 RCF at 4°C for 5 min, cells were resuspended in cold resuspension medium (40% FBS in RPMI) to a concentration of 20 × 10^6^ cells/ml. An equal volume of 2× freezing medium (30% DMSO, 40% FBS in RPMI) was added to give a final concentration of 10 × 10^6^ cells/ml. A cryo freezing container was used to freeze the cells overnight at −80°C. Cells were shipped overnight on dry ice for further processing.

### Single cell and library preparation for scRNA‐Seq for human placental macrophages

Cells were thawed at 37°C and immediately diluted with RPMI +10% FBS. Cell suspension was spun down at 300 RCF for 5′ and resuspended in 200 μL RPMI +10% FBS. Debris was removed by filtering cells through a pre‐wet 40 μm Flowmi strainer. Cells were spun again at 300 RCF for 5′ and resuspended in PBS + 0.04% BSA. Cells were counted and viability was assessed by trypan blue on a hemocytometer. Cells were run on the 10X Genomics Chromium Controller with the Single Cell 3′ v3 system. cDNA amplification and library preparation of the mRNA was processed according to 10X Genomics Single Cell 3′ v3 manufacturer's instructions.

### Single cell processing and filtering for human placental macrophages

Cell Ranger pipeline (V.3.0.0) was used for demultiplexing, mapping to the human genome (GRCh38) and counting UMIs for individual cells. Cells were filtered out if they had less than 500 unique molecular identifiers (UMIs), and if more than 20 or 15% of their transcripts were ribosomal or mitochondrial genes, respectively. In each placenta, cells from the two infection conditions with more abundant yields were down‐sampled in order to achieve roughly equivalent cell numbers between conditions for analysis. We normalized the expression to transcripts per median (TPM) and log transformed the expression. Each placenta was analyzed separately.

### Identification of *ex vivo* macrophage subtypes

Cell type profiles were established based on previously characterized cell types in human placenta (Tetz *et al*, 2015; Thomas *et al*, 2021). Specifically, multiple previously identified maternal macrophage subtypes were combined as were multiple stromal cell types. For one replicate, filtered cells were visualized with tSNE and clustered with hierarchical clustering. Maternal, Hofbauer and stromal clusters were identified by sum expression of combined genes from each of these profiles. To establish robust gene profiles for identification of the three major subtypes across samples, significantly upregulated genes unique to each of the three clusters were identified. Filtered cells from subsequent replicates were visualized with tSNE and clustered with hierarchical clustering. For each, normalized sum expression of uniquely expressed Hofbauer, maternal and stromal marker genes were visualized and cluster identity was assigned. If any cluster showed expression of stromal marker genes, it was eliminated from analysis and another round of tSNE and clustering was performed. This was repeated for each replicate until the only clusters remaining showed expression of HB or maternal macrophage genes.

### Cytokines and chemokines measurement

Cell culture supernatants were screened to measure the levels of multiple analytes (TNF‐α, IL‐6, IL‐1α, GRO (CXCL1), MCP‐1 (CCL2), MIP‐1α (CCL3) and MIP‐1β (CCL4)) using a customized version of the MILLIPLEX MAP Human Cytokine/Chemokine Magnetic Bead Panel (#HCYTOMAG‐60 k, MilliporeSigma). Samples were thawed on ice, centrifuged at 10,000 × *g* for 10 min to remove particulates, and then added to premixed beads, according to the manufacturer's protocol. A Luminex® 200TM instrument was used to collect and measure the Median Fluorescent Intensity (MFI) of 50 beads per analyte per well, following kit's instructions. The assay contained recombinant protein standards (Percentage of recovery within 75–120% range) and quality control (QC) samples (high and low concentration) for each analyte. A 4‐ or 5‐parameter logistic function with 1/y2 weighting was fit using Belysa® v.1 analysis software (MilliporeSigma) to calculate each cytokine's concentration. The lower limit of quantitation was defined by the software for each analyte. Samples with % CV that > 25% were excluded from analysis.

## Disclosure and competing interests statement

The authors declare that they have no conflict of interest.

## Supporting information



Appendix

## Data Availability

The datasets and code produced in this study are available in the following databases: scRNA‐Seq data: Gene Expression Omnibus accession number GSE196825 (http://www.ncbi.nlm.nih.gov/geo/query/acc.cgi?acc=GSE196825). Code for analysis: Github https://github.com/yanailab/GBS_placenta.
