# Supplementary material for: Host inflammatory dynamics reveal placental immune modulation by Group B Streptococcus during pregnancy
Source: Mol Syst Biol. 2023 Feb 6;19(3):e11021. doi: 10.15252/msb.202211021 (PMC9996236; doi:10.15252/msb.202211021)
Supplement: Supplementary file 1 — Appendix [file MSB-19-e11021-s002.pdf]

Host inflammatory dynamics reveal placental immune modulation by GBS during pregnancy

TABLE OF CONTENTS

	Page
Appendix Figure S1	2
Appendix Figure S2	3
Appendix Figure S3	5
Appendix Figure S4	7
Appendix Figure S5	9
Appendix Table S1	11
Appendix Table S2	13
Appendix Table S3	19
Appendix Table S4	20

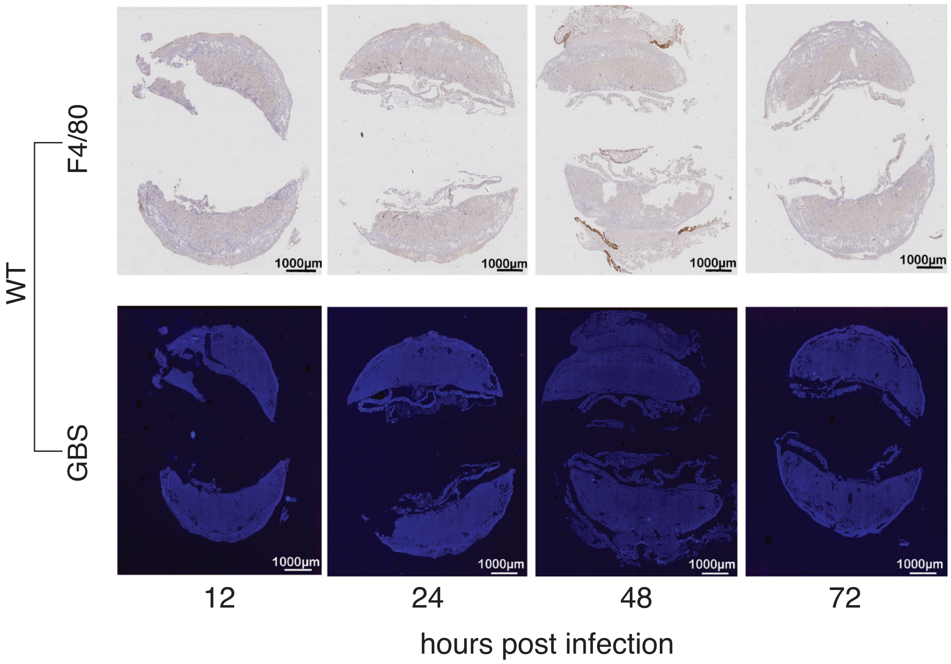

Appendix figure S1: Tissue-level phenotypic response in Sham-infected mice is not detected.

A Immunofluorescent staining of GBS in fixed placental sections from sham-infected mice. Corresponding F4/80 histology (indicating macrophages) of sequential placental sections from the same samples.

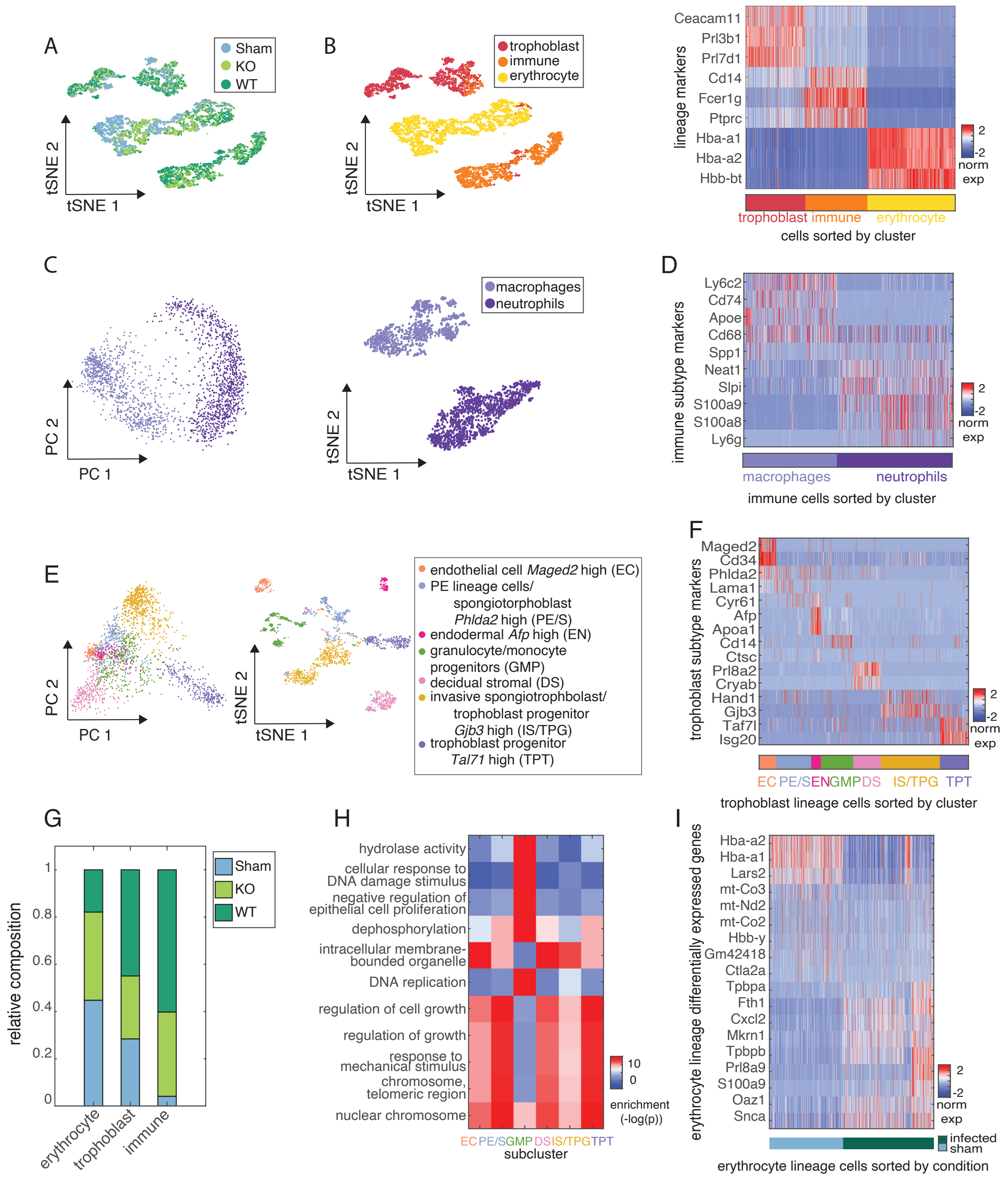

Appendix figure S2: Global cell lineage analysis reveals cell lineage-specific changes in response to infection.

A T-distributed Stochastic Neighbor Embedding (tSNE) on all cells from 48h post infection colored by condition.

B tSNE on all cells from 48h post infection colored by cluster identity (left). Z-scored expression of lineage marker genes independently curated on all cells from 48h post infection. Bar below indicates cluster identity (right).
C PCA (left) and tSNE (right) on immune lineage cells from 48h post infection colored by subcluster identity.
D Z-scored expression of marker genes unique to each subcluster in the immune lineage. Bar below indicates subcluster identity.
E PCA (top) and tSNE (bottom) on trophoblast lineage cells from 48h post infection colored by subcluster identity.
F Z-scored expression of marker genes unique to each subcluster in the trophoblast lineage. Bar below indicates subcluster identity.
G Bar plot of relative composition of cells from each condition per cell lineage cluster.
H GO enrichment of top differentially expressed genes identified by Ranksum test ($P< 0.0001$) in infected trophoblast subtypes excluding endodermal *Afp* high cells, which had no significantly upregulated genes in GBS-infected cells.
I Z-scored expression of differentially expressed genes identified by Ranksum test ($P< 0.0001$) genes between sham- and GBS-infected erythrocyte lineage cells. Bar below indicates infection condition.

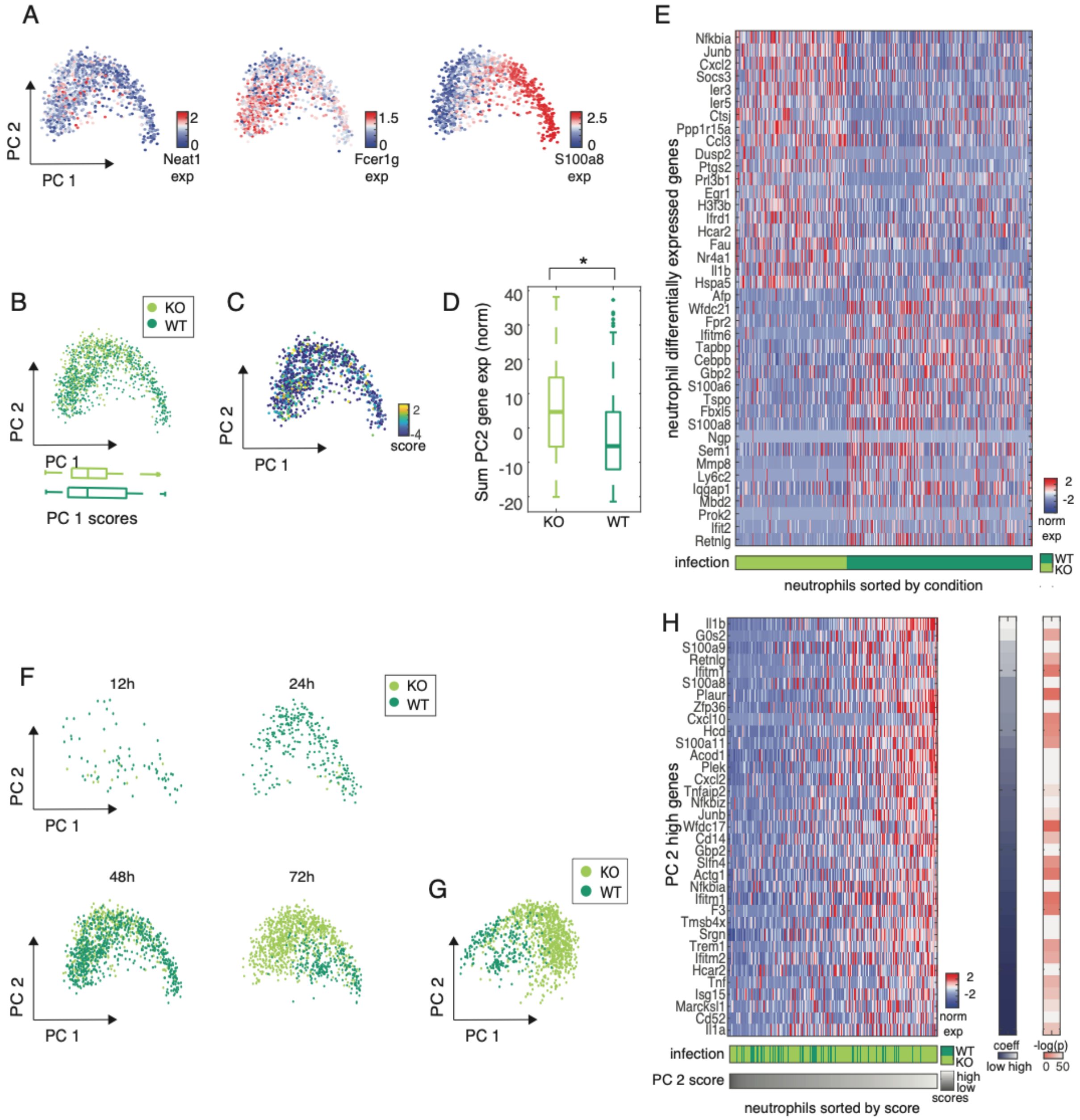

Appendix figure S3: Neutrophil cell state changes at different times following infection.

A Neutrophil PCA colored by expression of neutrophil subcluster marker genes (Han et al. 2018).
B PCA of neutrophils from mice 48h post infection and boxplot of PC 1 scores. Central band of the boxplot shows the median, boxes represent the IQR, and whiskers represent the most extreme values not considered outliers. (NS) The numbers of cells (biological replicates) included in the boxplots (KO, WT) are 418 and 700.
C PCA of neutrophils colored by cell cycle score (see methods).
D Boxplot of sum expression of PC2 contributing genes between conditions. Central band of the boxplot shows the median, boxes represent the IQR, and whiskers represent the most extreme

values not considered outliers. Significance was determined by paired t-test (*, $P<10^{-22}$). The numbers of cells (biological replicates) included in the boxplots (KO, WT) are 418 and 700.

E Z-scored expression of top differentially expressed genes identified by Ranksum test ($P<$ 0.0001) between KO- and WT-infected neutrophils at 48 hours post infection.

F Neutrophils from each time point plotted by scores calculated based on 72h neutrophil PCA (see methods).

G PCA on neutrophils from infected samples 72h post infection colored by infection condition.

H Neutrophils from 72h post infection are ordered by PC 2 score (bottom bar) and infection condition is indicated in middle bar. Coefficient of each gene is indicated in the bar on the right, and -log(p) of expression difference of that gene between KO- and WT-infected cells determined by paired t-test is indicated in the rightmost bar.

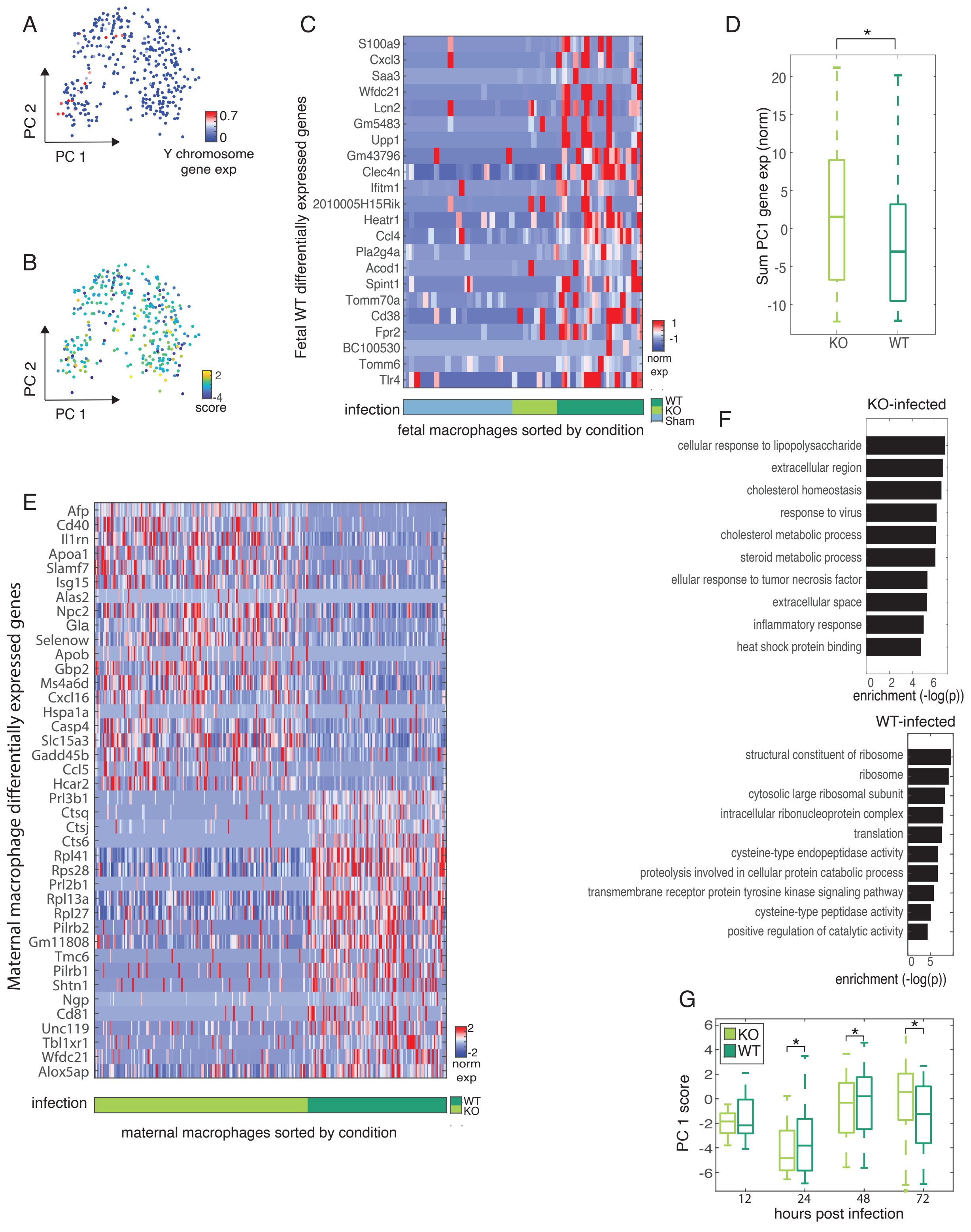
A
PC 2
PC 1
0.7
0
Y chromosome gene exp
B
PC 2
PC 1
2
-4
score
C
S100a9
Cxcl3
Saa3
Wfdc21
Lcn2
Gm5483
Upp1
Gm43796
Clec4n
Ifitm1
2010005H15Rik
Heatr1
Ccl4
Pla2g4a
Acod1
Spint1
Tomm70a
Cd38
Fpr2
BC100530
Tomm6
Tlr4
Fetal WT differentially expressed genes
1
-1
norm exp
infection
WT
KO
Sham
fetal macrophages sorted by condition
D
*
Sum PC1 gene exp (norm)
20
15
10
5
0
-5
-10
KO
WT
E
Afp
Cd40
Il1rn
Apoa1
Slamf7
Isg15
Alas2
Npc2
Gla
Selenow
Apob
Gbp2
Ms4a6d
Cxcl16
Hspa1a
Casp4
Slc15a3
Gadd45b
Ccl5
Hcar2
Prl3b1
Ctsq
Ctsj
Cts6
Rpl41
Rps28
Prl2b1
Rpl13a
Rpl27
Pilrb2
Gm11808
Tmc6
Pilrb1
Shtn1
Ngp
Cd81
Unc119
Tbl1xr1
Wfdc21
Alox5ap
Maternal macrophage differentially expressed genes
2
-2
norm exp
infection
WT
KO
maternal macrophages sorted by condition
F
KO-infected
cellular response to lipopolysaccharide
extracellular region
cholesterol homeostasis
response to virus
cholesterol metabolic process
steroid metabolic process
ellular response to tumor necrosis factor
extracellular space
inflammatory response
heat shock protein binding
0 2 4 6
enrichment (-log(p))
WT-infected
structural constituent of ribosome
ribosome
cytosolic large ribosomal subunit
intracellular ribonucleoprotein complex
translation
cysteine-type endopeptidase activity
proteolysis involved in cellular protein catabolic process
transmembrane receptor protein tyrosine kinase signaling pathway
cysteine-type peptidase activity
positive regulation of catalytic activity
0 5
enrichment (-log(p))
G
KO
WT
*
*
*
PC 1 score
6
4
2
0
-2
-4
-6
12
24
48
72
hours post infection

Appendix figure S4: Macrophage cell state changes at different times following infection.

A PCA on macrophages from 72h post infection colored by Y chromosome gene expression.

B PCA on macrophages from 72h post infection colored by cell cycle score (see methods).

C Z-scored expression of differentially expressed genes identified by Ranksum test (P< 0.0001) in WT-infected fetal macrophages from 72h post infection.

D Boxplot of mean normalized expression of PC1 contributing genes from Figure 4g between cells from each condition. Central band of the boxplot shows the median, boxes represent the IQR, and whiskers represent the most extreme values not considered outliers. Significance was determined by paired t-test (*, P<0.002). The numbers of cells (biological replicates) included in the boxplots (KO, WT) are 152 and 99.

E Z-scored expression of top differentially expressed genes identified by Ranksum test (P< 0.0001) between KO- and WT-infected maternal macrophages at 72h post infection.

F GO enrichment of top differentially genes identified by Ranksum test (P< 0.0001) from KO- and WT-infected samples.

G Boxplots of maternal fetal PC 1 scores at each time point post infection. Central band of the boxplot shows the median, boxes represent the IQR, and whiskers represent the most extreme values not considered outliers. Significance was determined by paired t-test (12h: NS, 24h:*, P<0.05, 48h:*, P<0.05, 72h:*, $P<10^{-3}$). The numbers of cells (biological replicates) included in the boxplots (KO, WT) are 56, 189 (12H); 43, 266 (24H); 171, 296 (48H); 152, 99 (72H).

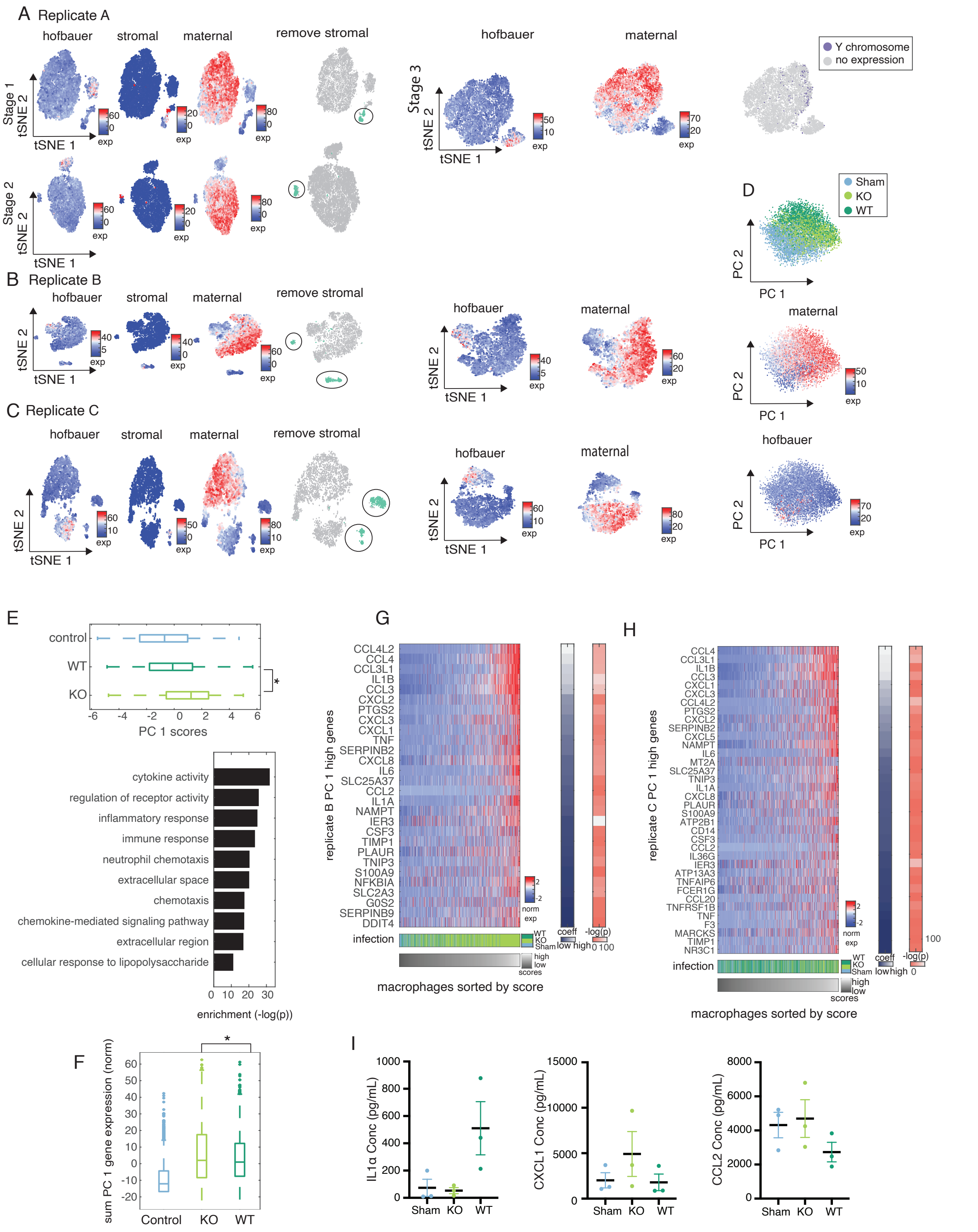
A Replicate A
hofbauer
stromal
maternal
remove stromal
Stage 1
Stage 2
Stage 3
tSNE 1
tSNE 2
exp
Y chromosome
no expression
B Replicate B
C Replicate C
D
Sham
KO
WT
PC 1
PC 2
E
control
WT
KO
PC 1 scores
cytokine activity
regulation of receptor activity
inflammatory response
immune response
neutrophil chemotaxis
extracellular space
chemotaxis
chemokine-mediated signaling pathway
extracellular region
cellular response to lipopolysaccharide
enrichment (-log(p))
G
replicate B PC 1 high genes
CCL4L2
CCL4
CCL3L1
IL1B
CCL3
CXCL2
PTGS2
CXCL3
CXCL1
TNF
SERPINB2
CXCL8
IL6
SLC25A37
CCL2
IL1A
NAMPT
IER3
CSF3
TIMP1
PLAUR
TNIP3
S100A9
NFKBIA
SLC2A3
G0S2
SERPINB9
DDIT4
norm exp
coeff
-log(p)
infection
scores
macrophages sorted by score
H
replicate C PC 1 high genes
CCL4
CCL3L1
IL1B
CCL3
CXCL1
CXCL3
CCL4L2
PTGS2
CXCL2
SERPINB2
CXCL5
NAMPT
IL6
MT2A
SLC25A37
TNIP3
IL1A
CXCL8
PLAUR
S100A9
ATP2B1
CD14
CSF3
CCL2
IL36G
IER3
ATP13A3
TNFAIP6
FCER1G
CCL20
TNFRSF1B
TNF
F3
MARCKS
TIMP1
NR3C1
infection
scores
macrophages sorted by score
F
sum PC 1 gene expression (norm)
Control
KO
WT
I
IL1α Conc (pg/mL)
CXCL1 Conc (pg/mL)
CCL2 Conc (pg/mL)
Sham
KO
WT

Appendix Figure S5: Analysis of human placental replicates.

A Filtering scheme for human *ex vivo* data of replicate A. Expression of previously identified marker genes (Thomas et al. 2021) for maternal macrophages, Hofbauer cells and stromal cells were measured in subpopulations defined by hierarchical clustering. Cells that clustered with maternal macrophages but that expressed Y chromosome genes were removed from analysis (see methods).

B Replicate B filtering scheme (see methods).

C Replicate C filtering scheme (see methods).

D PCA of Replicate A filtered macrophages corresponding to tSNE in Fig 5B colored by condition (top), sum expression of maternal macrophage marker genes (middle) and sum expression of Hofbauer cell marker genes (bottom).

E Boxplot of PC1 scores between conditions in maternal macrophages from placenta A with Y chromosome gene expressing maternal macrophages included (top). Central band of the boxplot shows the median, boxes represent the IQR, and whiskers represent the most extreme values not considered outliers. Significance was determined by paired t-test (*, $P<10^{-128}$). GO enrichment of PC 1 high genes (bottom). The numbers of cells (biological replicates) included in the boxplots (control, WT, KO) are 1814, 1904, 1802.

F Sum normalized expression of PC1 contributing genes between conditions. Central band of the boxplot shows the median, boxes represent the IQR, and whiskers represent the most extreme values not considered outliers. Significance was determined by paired t-test between KO- and WT- infected cells, (*$P<10^{-03<}$). The numbers of cells (biological replicates) included in the boxplots (control, WT, KO) are 1810, 1759, 1742.

G-H PC score analysis on placental replicates B & C. Z-scored expression of genes contributing to high PC 1 scores. Macrophages are ordered by PC 1 score (bottom bar) and infection condition is indicated in middle bar. Coefficient of each gene is indicated in the bar on the right, and -log(p) of expression difference of that gene between KO- and WT-infected cells determined by paired t-test is indicated in the rightmost bar.

I Luminex immunoassay of Il1α, CXCL1, and CCL2 levels on supernatants from human placental macrophage cultures. Each point is the average of two technical replicates, each the average of two plate replicates, with exceptions noted in the methods. Bars indicate mean + SEM between biological replicates in each condition.

mice infected E13	time	CFU/g	histology	Vaginal CFU/swab	Culture result of placentas for scRNA-seq	Included in scRNA-Seq analysis	Y chromosome gene transcript detected
Sham 1	12h	-	y	-	+	y	y
Sham 2	24h	-	y	-	+	y	y
Sham 3	48h	-	y	-	+	y	y
Sham 4	72h	-	y	-	+	y	y
KO 1	12h	-	y	$8x10^4$	+	y	y
KO 2	24h	$22x10^5$	y	$6x10^3$	-	y	y
KO 3	48h	$24x10^7$	y	$2x10^8$	+	y	y
KO 4	72h	$9x10^9$	y	$1x10^2$	+	y	y
WT 1	12h	$10x10^6$	y	$3x10^6$	+	y	n
WT 2	12h	$4x10^5$	n	$1.2x10^5$	+/-	y	n
WT 3	12h	-	n	$3x10^3$	-	n	N/A
WT 4	24h	$23x10^6$	y	$8x10^5$	+	y	y
WT 5	24h	$4x10^8$	n	$2x10^5$	+	y	n
WT 6	24h	-	n	-	-	n	N/A
WT 7	48h	$17x10^8$	y	$2x10^8$	+	y	y
WT 8	48h	$5x10^9$	n	$6x10^4$	+	y	n
WT 9	48h	-	n	$4x10^5$	-	n	N/A
WT 10	72h	$1x10^{11}$	y	$1x10^7$	+	y	y
WT 11	72h	-	n	$6x10^6$	-	n	N/A
WT 12	72h	N/A	N/A	N/A	N/A	N/A	N/A

Appendix Table S1: Treatment and allocation of mouse samples: 19 timed pregnant mice were treated on Day E13 of pregnancy, 4 in the sham group, 4 in the β-h/c KO group and the remaining mice in the WT GBS group (column 1). Mice were sacrificed at time post infection as indicated in column 2. From each mouse, two placentas were processed for scRNA-Seq (see Materials and Methods). CFU/g was determined from the left placenta most proximal to the cervix and CFU/g is indicated in column 3 (Fig 1B). The second most proximal (or third, if second most was used for scRNA-Seq) placenta from each mouse on the left side was fixed for histology and samples that were processed and included in analysis (Fig 1A) are indicated with a "y" in column 4. Vaginal swabs were performed for each mouse as an additional measure of systemic infection. CFU are normalized by volume of PBS and induced in column 5. From each mouse the two proximal placentas on the right side (or one right and one left if there were not two on the right side) were pooled and processed for scRNA-Seq. From each of those two placentas, a small piece of tissue was cultured for presence of GBS and results are indicated in column 5. If any tissue from a given mouse was culture positive, those samples were included in scRNA-Seq analysis (ie KO2) on the basis that the individual piece of tissue cultured from those placentas may not have shown a culture positive result, but that the mouse hadn't cleared the infection. Because CFU was not normalized by weight, culture results are recorded as positive or negative in column 6. One of the WT-infected mice sacrificed 12h post infection had one culture positive and one culture negative placenta for scRNA-Seq. Because placentas were pooled by hashing, but not deconvolved, the whole sample was included. Culture results may be negative for many technical reasons, but other measures indicate systemic infection. Column 7 indicates whether these samples were included in ("y") or excluded from ("n") scRNA-Seq analysis. Column 8 indicates whether any Y chromosome transcripts were detected in the scRNA-Seq data from that mouse indicating presence of at least one male fetus ("y" = detected and "n" = not detected).

Appendix Table S2:

Analysis	Gene	Coeff	P value
3D	*Il1b*	0.2055	1.86E-15
	Ccl4	0.2051	4.15E-11
	Cxcl2	0.1929	1.71E-20
	Ccl3	0.1776	3.61E-19
	Zfp36	0.1542	1.24E-08
	Il1a	0.1532	6.04E-05
	Nfkbia	0.1514	9.78E-38
	Cxcl10	0.1503	0.06870852248
	Ptgs2	0.1501	2.09E-09
	G0s2	0.1418	1.65E-05
	Cxcl3	0.1367	7.48E-05
	Acod1	0.1347	0.6853293765
	Nfkbiz	0.1344	0.009069889864
	Plaur	0.1305	0.00673936613
	F3	0.1281	6.86E-07
	Nlrp3	0.1239	6.20E-06
	Tnfaip2	0.1171	0.6050752177
	Socs3	0.1159	2.69E-26
	Hcar2	0.1149	2.47E-14
	Slfn4	0.1127	0.3313047688
	Tnf	0.1113	0.9494815239
	Plek	0.1087	0.06448744017
	Junb	0.1033	1.52E-25
	Ier3	0.1021	2.11E-18

	Cd14	0.0991	0.0006608022073
S3H	*Il1b*	0.2053	5.81E-23
	G0s2	0.1997	9.33E-11
	S100a9	0.1796	2.95E-34
	Retnlg	0.1758	3.47E-12
	Ifitm1	0.1745	0.0009110457307
	S100a8	0.1531	1.86E-60
	Plaur	0.1525	0.08182655534
	Zfp36	0.1518	6.98E-25
	Cxcl10	0.1489	1.09E-05
	Hdc	0.1484	1.03E-06
	S100a11	0.141	7.66E-09
	Acod1	0.132	5.94E-64
	Plek	0.1315	7.47E-24
	Cxcl2	0.1282	7.04E-30
	Tnfaip2	0.1239	8.05E-20
	Nfkbiz	0.1217	5.41E-23
	Junb	0.1216	1.75E-19
	Wfdc17	0.1189	0.5766993783
	Cd14	0.1167	2.63E-14
	Gbp2	0.1126	2.98E-25
	Slfn4	0.1116	5.04E-08
	Actg1	0.1102	0.0007809782324
	Nfkbia	0.1084	2.47E-42
	Ifitm3	0.1044	0.00933146941
	F3	0.1039	0.0001632197463

	Tmsb4x	0.1013	8.10E-34
	Srgn	0.0997	5.47E-29
	Trem1	0.0995	9.35E-10
	Ifitm2	0.0986	1.34E-11
	Hcar2	0.0965	2.16E-37
	Tnf	0.0955	7.62E-13
	Isg15	0.0953	3.53E-15
	Marcksl1	0.0951	1.05E-19
	Cd52	0.0948	5.41E-44
	Il1a	0.0943	1.61E-15
4G	*Saa3*	0.2058	0.6765
	Il1rn	0.1355	0
	Nos2	0.1344	0.6987
	Lcn2	0.1116	0.0025
	Ccl5	0.1086	0.0003
	Ptges	0.1076	0.0176
	Ccl4	0.1062	0.0012
	Slamf7	0.106	0
	Basp1	0.1044	0.5368
	AA467197	0.1042	0.0009
	Inhba	0.1007	0.2584
	Slpi	0.0913	0.5631
	Slc7a2	0.0913	0.0457
	Rsad2	0.0885	0.1464
	Il7r	0.0872	0.2069
	Spp1	0.0866	0.5089

	Hmox1	0.0858	0.4643
	Mt2	0.0838	0.5253
	Mt1	0.0838	0.8936
	Ccl3	0.083	0.7425
5C	*CCL4L2*	0.2863	0
	IL1B	0.2805	0
	CCL4	0.2609	0.3446
	CCL3L1	0.2487	0.0441
	CCL3	0.2181	0.0388
	CXCL3	0.1945	0.0014
	CXCL1	0.1861	0
	CXCL2	0.1827	0
	SERPINB2	0.1628	0
	IL6	0.1583	0
	PTGS2	0.1546	0
	IER3	0.124	0
	TNF	0.1168	0.6031
	NAMPT	0.1068	0
	CCL2	0.1052	0
	CXCL8	0.0998	0
	CSF3	0.0915	0.0042
	PLAUR	0.0854	0.0523
	TNIP3	0.0834	0
	S100A9	0.0819	0
	BCL2A1	0.0789	0.0002
	NFKBIA	0.0746	0

	MARCKS	0.0738	0
	SLC25A37	0.0734	0
	IL1RN	0.0727	0.0114
	FCER1G	0.068	0
	IL36G	0.0674	0
	CSF2	0.0659	0.0001
	EREG	0.063	0.2824
	IL1A	0.0623	0.0002
	TIMP1	0.0586	0
S5G	*CCL4L2*	0.302	0
	CCL4	0.2798	0
	CCL3L1	0.2596	0
	IL1B	0.2533	0
	CCL3	0.2341	0
	CXCL2	0.1796	0
	PTGS2	0.1725	0
	CXCL3	0.1563	0
	CXCL1	0.1515	0
	TNF	0.136	0
	SERPINB2	0.1356	0
	CXCL8	0.1167	0
	IL6	0.1166	0
	SLC25A37	0.1131	0
	CCL2	0.1068	0.0042
	IL1A	0.1044	0
	NAMPT	0.0966	0

	IER3	0.0827	0
	CSF3	0.0822	0
	TIMP1	0.0816	0.0074
	PLAUR	0.0794	0
	TNIP3	0.0746	0
	S100A9	0.0712	0.3428
	NFKBIA	0.071	0
	SLC2A3	0.069	0
	G0S2	0.0624	0.0013
	SERPINB9	0.0596	0.0010
	DDIT4	0.0573	0.2940
S5H	*CCL4*	0.2605	0
	CCL3L1	0.2532	0
	IL1B	0.2499	0.0017
	CCL3	0.2425	0
	CXCL1	0.2081	0.2163
	CXCL3	0.1927	0.0183
	CCL4L2	0.1837	0
	PTGS2	0.1669	0
	CXCL2	0.1548	0.0001
	SERPINB2	0.1455	0
	CXCL5	0.1336	0.1853
	NAMPT	0.1309	0
	IL6	0.1173	0
	MT2A	0.1019	0.0565
	SLC25A37	0.0986	0.1476

	TNIP3	0.0914	0
	IL1A	0.088	0
	CXCL8	0.0869	0.0007
	PLAUR	0.0864	0.9215
	S100A9	0.0849	0.3852
	ATP2B1	0.0832	0.0004
	CD14	0.0829	0.4124
	CSF3	0.0805	0.1766
	CCL2	0.0792	0.0002
	IL36G	0.0706	0.0083
	IER3	0.0684	0
	ATP13A3	0.0655	0.0001
	TNFAIP6	0.0645	0.3363
	FCER1G	0.0637	0
	CCL20	0.063	0.4087
	TNFRSF1B	0.0605	0
	TNF	0.0538	0.0004
	F3	0.0492	0.2081
	MARCKS	0.0484	0.0337
	TIMP1	0.048	0.1347
	NR3C1	0.0476	0.0012

Appendix Table S3:

Hofbauer genes: *SPP1, TNFRSF4, SGK1, EMP3, EEF1A1, TNFRSF18, ACTG1, PLD3, LPL, PLIN2, JUN, IQGAP2, GPNMB, GADD45B, P2RX7, HMGA1, ETS2, CCDC50, DAB2, CBLB, SQSTM1, MATK, RGCC, OXR1, SLC16A10, NEURL3, TNFRSF12A, FNIP2, IL1RN, SNX8, CD84, HS3ST2, RASGEF1B, EEF1B2, CD9, KLF6, EGR2, DDIT3, CAST, SH3BGRL3, FMNL2, HSP90AB1, ACTB, PDGFA, MYOF, EPS8, RTN4, ACADVL, CD36, MT-ND3, IRF1, IFRD1, ARHGAP10, PLEKHO1, CCND1, GBP2, ANKRD28, HAVCR2, CD109, RCAN1, ANXA5, CD40, NANS, ST3GAL6, ANXA4, CEBPB, PARP14, SRFBP1, SCIN, ABCE1, ITGB5, OGFRL1, DDX21, YBX1, RBMS1, TMEM273,NMB, PPARG, GNG12, RPL10A, LACTB, C6orf48, CXCL16, SLCO4A1, PLA2G16, MT-CYB, ANKRD12, PIM3, WSB1, SEMA6B, EMP1, JAK1, CTSD, TNS3, CD276, PRR5, HPGDS, RPL22L1, CD86, MGAT1*
Maternal genes: *FCER1G, IL1B, TNFAIP6, EREG, SLC7A11, BASP1,SLC25A37, TXN, MGST1, C15orf48, CYBA, SERPINB2, ITGB8, CD48, PILRA, CXCL1, CSTB, AQP9, C1orf122, ADAMDEC1, FTH1, THBS1, MARCKS, CXCL2, CXCL3, IL7R, ANPEP, SERF2, HLA-DRB1, SERPINA1, SRGN, CLEC4E, HLA-DQA1, TSC22D1, MET, CYP1B1, B2M, MYO1G, IL36G, MT-CO1, SLC39A8, PLAC8, MT1E, CXCL5, GM2A, CCL3, RPL36AL, MSC, MT1X, IFI6, PTGS2, MMP14, CSTA, TNIP3, MAP3K20, SEC61G, NCF2, HLA-A, CREG1, ADA, LYZ, S100A9, CAPG, DNAJC3, ATP13A3, HLA-DRA, SERPINB9, CTSH, CARD16, TALDO1, MTRNR2L12, SLC7A7, EHD1, OAZ1, PLD1, MT1F, OST4, BRI3, CXCL8, DYNLL1, PLAUR, S100A8, CYB561A3, IRAK3, MT1M, IL19, TIMP1, IL2RA, LYN, ARPC5, SLAMF7, PTGIR, ATP5F1E, IL10, IDO1, NAMPT, CYTIP, CES1, S100A11, S100A10*
Stromal genes: *CYR61, IL1RL1, SERPINE2, LMCD1, APOD, C7, CXCL14, SPARC, CTGF, IGFBP3, CALD1, RARRES2, EGFL6, GPC3, CRYAB, KRT18, LUM, DCN, DLK1, CDH11, COL1A1, TIMP3, FBLN1, COL6A1, COL6A2, SERPINE1, SERPINF1, COL3A1, SRPX, PTGDS, CNN3, FSTL1, WNT2, SOD3, AKAP12, AEBP1, HGF, DKK1, RSPO3, COL4A2, ADAM12, IGF2, TAGLN, COL4A1, FST, PLOD2, SELENOM, TFPI2, PCOLCE2, SLC22A3, CLMP, COL14A1, MGP, MFAP4, ERRFI1, LARP6, ID3, C1R, PCOLCE, CCDC80, TGFBI, COL1A2, EXT1, FBLN2, FRMD6, ABI3BP, CAVIN1, FHL2, IGFBP5, MSX1, OSMR, PLAC9, TBX3, FGF7, DKK3, MAP1B, VGLL3, UCK2, MYL9, EPAS1, CLU, COL5A1, EFEMP1, FHL1, RRAS2, SPTBN1, IGFBP2, COL15A1, MYC, MEST, MMP23B, MMP2, TEAD1, ENPP1, PRRX1, EGFR, SEMA3B, BDKRB1, DPT, IL13RA2*

Appendix Table S4:

G1_S genes: *Mcm6, Exo1, Dtl, Cdca7, Rad51, Wdr76, Pola1, Ccne2, Casp8ap2, Usp1, Nasp, Clspn, Rpa2, Tyms, Slbp, Ung, Rfc2, Mcm2, Rad51ap1, E2f8, Blm, Pold3, Rrm1, Prim1, Mcm5, Gins2, Tipin, Brip1, Cdc6, Gmnn, Rrm2, Ubr7, Dscc1, Atad2, Mcm4, Cdc45, Chaf1b, Uhrf1, Msh2, Pcna-ps2, Fen1, Hells*
G2 genes: *Hjurp, Nuf2, Lbr, Cenpf, Nek2, Tubb4b, Ckap5, Nusap1, Bub1, Ckap2l, Tpx2, Ube2c, Aurka, Ect2, Smc4, Cks1b, Anp32e, Psrc1, Cenpe, Kif2c, Cdc20, Cdca8, Cenpa, Tacc3, Cdca3, Ncapd2, Mki67, Cdk1, Gas2l3, Tmpo, Ckap2, Hmgb2, Ctcf, Dlgap5, Cdca2, Anln, Kif23, Ccnb2, Ttk, Hmmr, Aurkb, Top2a, Birc5, Cks2, G2e3, Rangap1, Gtse1, Cbx5, Ndc80, Cdc25c, Kif20b, Kif11*
