# Supplementary material for: Host inflammatory dynamics reveal placental immune modulation by Group B Streptococcus during pregnancy
Source: Mol Syst Biol. 2023 Feb 6;19(3):e11021. doi: 10.15252/msb.202211021 (PMC9996236; doi:10.15252/msb.202211021)
Supplement: Supplementary file 2 [file MSB-19-e11021-s001.pdf]

molecular systems biology

Host inflammatory dynamics reveal placental immunomodulation by GBS during pregnancy

Felicia Kuperwaser, Gal Avital, Michelle Vaz, Kristen Noble, Allison Dammann, Tara Randis, David Aronoff, Adam Ratner, and Itai Yanai
DOI: 10.15252/msb.202211021

Corresponding author(s): Itai Yanai (itai.yanai@nyulangone.org)

Review Timeline:

Submission Date:	23rd Mar 22
Editorial Decision:	19th Apr 22
Revision Received:	8th Nov 22
Editorial Decision:	16th Dec 22
Revision Received:	6th Jan 23
Accepted:	10th Jan 23

Editor: Maria Polychronidou

Transaction Report:

(Note: With the exception of the correction of typographical or spelling errors that could be a source of ambiguity, letters and reports are not edited. Depending on transfer agreements, referee reports obtained elsewhere may or may not be included in this compilation. Referee reports are anonymous unless the Referee chooses to sign their reports.)

1st Editorial Decision 19th Apr 2022

Thank you again for submitting your work to Molecular Systems Biology. We have now heard back from the three reviewers who agreed to evaluate your study. As you will see below, the reviewers think that the study seems interesting. However, they raise a series of concerns, which we would ask you to address in a revision.

The comments of the referees are rather clear and I think that there is no need to repeat them here. All issues raised by the referees would need to be satisfactorily addressed. Please let me know in case you would like to discuss in further detail any of the issues raised, I would be happy to schedule a call.

On a more editorial level, we would ask you to address the following points:

Reviewer #1:

Felicia Kuperwaser et al., show that GBS modulates innate immune cell states by subversion of host inflammation through β-h/c. The authors used an ascending mouse model of GBS infection to measure placental cell state changes over time following infection with a β-h/c-deficient and isogenic wildtype GBS strain. The authors tested the results in human placental macrophages. Transcriptomic analysis suggests that β-h/c-producing GBS elicit a worse phenotype through suppression of host inflammatory signaling in placental macrophages and neutrophils. The findings are important and valuable to the reproductive biology community and have implications for the identification of potential targets in GBS disease.

Major points:

1. The cell state differences between KO and WT are relatively small in most cases. Indeed, they are overlapping in most cases. Are the scRNA-seq analysis sensitive enough for such small variances?
2. Lack of strong functional validation assays.
3. Most of the figures are shown in PCs. Can the authors justify why they use PCs vs tSNEs, UMAP or diffusion map?
4. Why do they use tSNE for human single-cell data and PC for mouse data?
5. The authors need to explain how they annotate cell types and subtypes better. Which genes, gene signatures?
6. The authors need to define how Y-chromosome genes are calculated.
7. Can the authors explain cell type-specific transcriptional changes in each subtype of trophoblast, immune cells in more detail?

Minor points:
1. In Figure 1, texts in scale bars are too small.
2. Figure 2d, several dots are missing in the cell type labels
3. Overall, figures need to be better scaled and organized.

Reviewer #2:

In this study, the authors sough to investigate the placental cellular mechanisms implicated in ascending GBS infection during pregnancy, and the role of β-hemolysin/cytolysin (β-h/c) in such a process. The authors performed time-course sampling of placentas from mice infected with wild type (WT) or β-h/c-deficient (KO) GBS at 12 - 72 h post-infection for single-cell RNA-sequencing (scRNA-seq) as well as histological analyses. Moreover, placental macrophages from women undergoing term cesarean section were utilized for ex vivo assays followed by scRNA-seq. First, the authors demonstrated that the placental GBS burden is reduced in KO-infected dams; nevertheless, infection with either strain resulted in increase co-localization of GBS and macrophage marker expression in the placenta. Bioinformatics analysis of scRNA-seq data implicated placental immune cells as the primary population affected by GBS infection, with neutrophils and macrophages being the primary cell types involved. Notably, the authors observed that both neutrophils and macrophages from KO-infected mice have reduced inflammatory gene expression in early infection phases (prior to 48 h) compared to those cells from WT-infected mice; however, in late infection, cells from KO-infected mice show greater expression of inflammatory genes. Such macrophage responses primarily involved maternal cells in both infection conditions; yet, WT-infected mice also showed a fetal macrophage response in late infection (72 h). As validation, the authors exposed human placental macrophages to WT or KO GBS ex vivo and found that KO-infected macrophages displayed a greater inflammatory expression profile compared to the WT-infected cells. The authors propose a model whereby β-h/c contributes to poor perinatal outcomes due to GBS infection by subverting the inflammatory responses of placental neutrophils and macrophages, thereby preventing appropriate immune activation and pathogen clearance.

Overall, this well-designed and straightforward study addresses important mechanisms of ascending microbial infection during pregnancy. Specific comments are as follows:

1. Results: What are the pregnancy outcomes of GBS infection using the WT and KO strains? Do these mice undergo preterm birth, and are there differences between strains in terms of preterm birth rate, neonatal mortality, etc.? Given that the peak inflammatory response seems to occur at 72 h (approximate to 16.5 days post coitum), the occurrence of preterm birth shortly after this point would be consistent.
2. Results: Could the authors expand the discussion of their findings related to fetal macrophage activation with WT infection? Is the lack of such a response in the KO infection model due to the preservation of anatomical barriers, or is it driven by the changes in gene expression/cellular function? Differences in the fetal inflammatory response and fetal bacterial load between strains would also be interesting, yet this may be outside the scope of the current study.
3. Results: The observation in Figure 5 that WT GBS drives a different set of genes is interesting and would benefit from additional analysis and/or discussion. What sort of pathways/functions are this gene set implicated in? Such analysis could help complement the final discussion paragraph in which the authors mention specific genes such as BHLHE40.
4. Discussion: The first paragraphs of the discussion are a little bit results-heavy, perhaps the key findings can be summarized in one short paragraph without too much repetition of results or methods.
5. Introduction: The authors should consider including several additional studies showing the involvement of macrophages at the maternal-fetal interface in pregnancy maintenance as well as adverse pregnancy outcomes.

Reviewer #3:

Summary:
This study conceptually advances our understanding on how vertical transmission of Group B Streptococcus (GBS) modulate immune cells function and response in the mouse and human placenta. GBS is commonly found in the vagina and can pass from pregnant mother to her fetus during labour. GBS can also ascend from the vagina to upper reproductive tract during pregnancy and can cause severe consequences. This study utilized the murine vaginal colonization model to study ascending infection phenotype over time. Specifically, the authors suggested, in part, that beta-hemolysin/cytolysin as the major GBS virulence factor responsible for switch in inflammatory signalling in placental macrophages and neutrophils. The authors take advantage of single-cell genomics profiling on infected mouse placentas and human placental macrophages. However, there are several points and validation that must be adequately addressed before the paper can be accepted.

Major comments:
1. There are many details missing in the methods section regarding the analysis of scRNA-seq data. In order to make this study reproducible and clearer for the reader, the authors should include: how the clusters were obtained, method used to get the marker genes/differentially expressed, number of cells analysed, QC thresholds used for the ex vivo macrophage analysis, among any other detail that the authors consider it's important for future reproducibility.
2. There are inconsistencies in the values plotted in Figure 1b, which do not match those in table 1. In addition, it is not possible to make a reliable comparison in Figure 1b using n=1 to see if the CFU/gram of placenta is decreased in KO-infected placentas. Even if the presence of GBS seems to be reduced in Fig 1c-d. Bacteria burden in CFU/g counts does not match counts in Table 1 where CFU/g is indicated in column 3. For example, at time-point 72 hr, in Table 1 KO4 shows 9x10^9CFU/g and WT4 shows 1x10^9 CFU/g. Whereas the scatter plot in Figure 1b at time-point 72 hr shows that WT 10^11 CFU/g range. The discrepancy is at 2-log fold change and the conclusion will need to be re-addressed if this is true.
3. The authors suggest there is a gradient of erythrocyte differentiation revealed by the PC1 scores in Figure S2c. A more detailed analysis is needed to claim this, as it could only be the stress elicited by the infection, and not a differentiation process.
4. I would suggest to refine the scRNA-seq analysis. The major cell types are very constrained in the projection to be able to see all the differences between and within them mentioned by the authors. For example, the fact that trophoblast and immune cells are overlapping suggest that using PCA as their dimensionality reduction method is barely enough to differentiate between quite different cell types. Therefore, using this projection to look into sub-type differences is quite bold (e.g. Figure 2d where granulocyte/monocyte progenitors are clustered with trophoblasts; the impossibility to recognise the 7 sub-types reported by the authors in Figure 2d; or the presence of only two immune cell types in Figure 2c). I would suggest trying a different dimensionality reduction algorithm (e.g. t-SNE or UMAP) to test if they can obtain a better resolution.
5. With Figure 2e-f the authors claim that "This confirms that innate immune cells undergo transcriptional changes in response to infection". Even though, the high proportion of immune cells in the analysed tissue is directly linked to the infection (i.e. infection = more immune cell recruitment), the imbalance in the dataset (<5% of cells coming from Sham controls) makes difficult to take this comparison as a "confirmation" of significant transcriptional changes in innate immune cells. I have no doubt this might be the case, but the authors cannot assure this with the evidence provided. If the authors want to demonstrate this conclusion, I would suggest them to sequence more innate immune cells from uninfected controls. As the presence of these cells in the healthy tissue seems to be very low, I can suggest do an enrichment of immune cells using FACS or MACS. The authors can include this new data into the current dataset and then perform more reliable comparisons.
6. The authors found that many genes contributing to PC2 are related to an IL-1-family inflammatory response, Figure 3d. Then, the authors mention -"We were therefore surprised to observe that, based on PC 2 scores, the KO-infected cells exhibited higher levels of inflammatory gene expression (Figure 3c)"-. The authors must remember that a PC is the linear combination of many variables (i.e. the expression of different genes). Even if there is a correlation between inflammation and the PC2, the authors cannot interpreted PC2 solely as inflammation nor use the PC values to measure differences in inflammation as done in the quoted text, in Figure 3c and Figure 3e. The GO This is also done in Figure 4e, where they label the Y-axis "Inflammatory gene expression", when they are actually plotting PC1 scores. This is highly misleading, and also conceptually wrong, so it must be corrected. Other figures with this approach are Figure 4f, S4e and 5d. As one of the main results of the authors is that β-h/c KO-infected GBS induce higher IL-1 family inflammatory response, meaning that WT GBS might have an anti-inflammatory effect, the authors should change this approach of doing "PC score analysis". The authors can measure the inflammatory/anti-inflammatory response directly instead of using ambiguous PC scores to try to support their conclusions.
7. The authors claimed in-frame cylEΔcat mutant (referred to as β-h/c KO) were used. It is unclear how this mutant is constructed (i.e through allelic exchange?). In bacterial transformation strategy using allelic exchange mutagenesis, the KO phenotype has to be confirmed through complementation analysis where a wild-type copy will need to be re-introduced into β-h/c KO. This to ensure that phenotype observed in Figure 1 (and subsequent assay) is due to cylEΔcat specific gene disruption, and not through polar effects on downstream genes or operons in the β-h/c KO strain. Polar effect is especially common for bacterial ORFs, if not validated.
8. Figure 1c, 1d: Provide overlapping/colocalization images on GBS, F4/80, and a marker of fetal or placental membranes to justify claim in line 112 page 4 "We observed infection along the fetal and maternal placental membranes with corresponding increase in macrophage staining in the same heavily-colonized tissue regions". How do you exclude that GBS is not colonizing the murine decidua, and specifically the placental membrane?
9. Page 8 line 181: "β-h/c KO GBS-infected neutrophils show higher inflammatory response..." This statement is misleading as the authors did not show that GBS directly infects neutrophils in the mouse placenta. The following paragraph also suggested the term 'infected cells', which needs to validated if indeed neutrophils is GBS-infected.
10. Page10 line 236: 'KO GBS-infected maternal, but not fetal macrophages....' What is the CFU/bacterial burden difference

between KO and WT GBS in ex vivo placental macrophage infection assay? Can the author confirm the differences in CFU observed in mice in the infected placental macrophages?
11. It is unclear what is rationale of performing the ex vivo human placental macrophage infection assay for only 3 hours (1 time-point) yet the dynamics of immune cell response of early versus late (12hr vs 72hr) infection in the GBS mouse model Figure 3e and Figure 4e. Along this line, the abstract claims "measure placental cell states changes over time......recapitulated our results in placental macrophages." This is misleading as no time-point experiment was done for the ex vivo human placental macrophage infection assay.
12. Figure 4g: Cytokine activity in maternal macrophage is significantly modulated between KO and WT GBS stimulation, based on transcriptional profiling. Can the author validate this from the culture supernatant using simple ELISA or Western blot assay?
13. Page14 line 336: To my knowledge, mouse Hofbauer (HB) macrophages have never been profiled in detail and have never been compared to those from human placentas. It is too generalized to disregard the role of human HBs from Figure 5 which may a role in GBS infection despite not detecting any differences in mouse fetal macrophages. Unless authors perform correlation analysis between mouse fetal macrophages' and human HBs expression profile.

Minor comments:
1. It is very difficult to see the different populations if the authors use many tones of the same color within a plot (e.g., Figures 2b, 2d, S2b). I would suggest the authors to use a more distinguishable color palette for these plots.
2. Scale bars in Figure 1c-d are too small to be understandable. Authors should make them visible to the reader.
3. There are not details about what kit was used for cell hashing, nor which HTO oligos. This is a relevant detail for data analysis.
4. The plots for "Hofbauer cells" and "Y chromosome" in Figure 5b do not match their position written in the figure caption.
5. The authors must specify which genes were used to plot the sum expression for Hofbauer cells and maternal macrophages in Figure 5b, S5a, S5c and S5d. The methodology to do this sum should be explained in the methods section, as it's not clear if the author are just summing the expression of all the genes used as markers.
6. The higher expression of those genes contributing to PC1 in clusters with high PC1 score is expected, Figure 5e. What is the message from this plot?
7. The authors should mention/show which are the genes that would support their hypothesis that β-h/c actively suppress inflammation through the induction of suppressive and regulatory genes. Figure 5f.
14. General points on single-cell analysis:
• Please describe in methods section how on data processing, filtering and integration pipeline given that the general interest for MSB readers are in the field of system biology.
• How are the hashtag reads being incorporated into the data?
• Is cell cycle a major source of variation in the PCA?
• How are the samples/cell states integrated for analysis?
• Perform downstream differential gene expression analysis (with statistical test) to confirm PCA variation at gene level between KO and WT GBS induced infection
• FigureS5 is not clear what previously identified markers were used for filtering scheme. Please plot all identified markers on the umap.
• Figure 5b What statistical test were used to PC1 scores?
• It is not clear if all three human placenta macrophages were integrated for figure5C, figure 5e and figure 5f, whereas figure5b only show placenta A?
• UMAP from FigureS5 should be properly integrated by donors.
15. Discussion section: There is no discussion on the limitation on using ascending intravaginal GBS infection model in mouse. First, the vagina microenvironment (pH, microbiome, antimicrobial peptides) and mucosal immunity in mouse is very different from the human. Second, the mouse and human placenta is different in terms of developmental stages, and this will likely have an impact of immune cell properties and responses to infection. It is important to inform the general readers who may not have reproductive immunology background.

Response to reviewers

Reviewer #1:

Summary: "Felicia Kuperwaser et al., show that GBS modulates innate immune cell states by subversion of host inflammation through β-h/c. The authors used an ascending mouse model of GBS infection to measure placental cell state changes over time following infection with a β-h/c-deficient and isogenic wildtype GBS strain. The authors tested the results in human placental macrophages. Transcriptomic analysis suggests that β-h/c-producing GBS elicit a worse phenotype through suppression of host inflammatory signaling in placental macrophages and neutrophils. The findings are important and valuable to the reproductive biology community and have implications for the identification of potential targets in GBS disease."

Response:

We would like to thank the reviewer for the time and effort that they have dedicated to reviewing our work. We believe that our revised manuscript has greatly improved thanks to their constructive comments, which broadly fall into three categories: 1. Request for additional validations, 2. questions regarding our approach, and 3. Clarity and presentation. In response, we have done additional experiments (Concern 2), tested for the robustness of the analyses (Concern 1 & 3), clarified the choice of our data visualization (Concern 4), clarified our cell type and subtype annotation method (Concern 5 & 6), introduced additional analysis on cell type-specific changes (Concern 7), and improved presentation of data (Concern 8, 9 & 10). We believe that we have addressed all of Reviewer 1's concerns and we thank them again for their efforts.

Concern 1: "The cell state differences between KO and WT are relatively small in most cases. Indeed, they are overlapping in most cases. Are the scRNA-seq analysis sensitive enough for such small variances?"

Response: We thank the reviewer for raising this important concern, which prompted us to modify the manuscript text to better explain our statistical framework.

While we have taken extensive steps towards analyzing the data in a way that distinguishes true signal from noise, the possibility of false positives always remains in scRNA-Seq analysis. Therefore, we have validated our core claim in an orthogonal system at both the transcriptomic and protein levels (Figure 5 and Appendix figure S5). The reviewer is absolutely correct that the difference captured in the *in vivo* data is subtle, given that the response program between conditions is largely conserved, reflecting the innate immune system's ability to respond efficiently to diverse bacterial stimuli. It is for precisely this reason that we would not have been able to observe this difference without both the use of scRNA-Seq data, which allows us to capture these subtle differences

within appropriately matched cell subtypes, and the use of time course data which strengthens our comparative framework.

In this investigation, we set out to measure the cell-specific transcriptional differences that underlie the dramatic tissue-level differences between these conditions. We originally expected that host inflammatory damage, mediated by the responding immune cells, was responsible for the tissue-level phenotypic and outcome differences observed. Therefore we expected a higher inflammatory response in the WT-infected mice than the KO-infected. It is only because of the scRNA-Seq data that we were able to observe precisely the opposite of what we hypothesized, as this signal may have been overshadowed otherwise, and while the *in vivo* differences were relatively subtle, the fact that we were able to validate them in another system provided important evidence that the observed differences are biologically meaningful. This is also why we measured statistical significance of these differences and validated at the protein level.

Action: In response to this concern, we have updated the relevant text from the Discussion to acknowledge the reviewer's point and clarify how this approach was integral to revealing these results:

> "In this work, we defined the placental cell state dynamics in response to GBS with or without expression of the toxin β-h/c. We first recapitulated the difference in tissue-level phenotype between the response to a β-h/c KO and isogenic WT GBS strain (Randis et al. 2014), using a mouse model of ascending infection. We found that WT GBS induced a more severe phenotype measured by histological analysis and bacterial burden than the β-h/c KO (consistent with prior studies). We analyzed corresponding scRNA-Seq data to understand the cell state changes that accompanied this phenotypic progression. PC score analysis revealed GBS induction of an IL1 family inflammatory response in innate immune cell populations, but different response dynamics depending on the presence of β-h/c. In the first stage after infection (up to 48h), immune cells from WT-infected mice expressed relatively higher inflammatory response genes than those from β-h/c KO-infected mice. In the later stage however, (48-72h), we observed relatively less inflammatory expression in cells from the WT- compared to the β-h/c KO-infected mice, concurrent with the presentation of severe phenotype in WT-infected mice. We found that fetal macrophages were only activated when infected with WT GBS at 72h post infection. These data reveal a largely conserved response between conditions, but our systematic study design and use of scRNA-Seq data as a primary readout reveals subtle shifts in response dynamics that can provide important insight into the tissue-level responses between conditions. While the scRNA-Seq differences are subtle, we validated the relatively lower expression of inflammatory response genes induced by WT- compared to β-h/c KO-GBS in maternal macrophages and neutrophils in an *ex vivo* infection of human placental macrophages, where we found a robust and reproducible difference between conditions."

Concern 2: “Lack of strong functional validation assays.”

Response and Action: We thank the reviewer for this comment. In response to it, we sought to validate expression level differences of a set of representative genes that are significantly upregulated in KO-infected over WT-infected cells from both the human placental macrophage data as well as the *in vivo* mouse data. To do so, we measured protein levels of this subset of genes from cell culture supernatants from human placental macrophages infected with GBS. These experiments were very important in validating the observations made from our transcriptomic system and lay the groundwork for future studies which will address the mechanism of host inflammatory subversion by GBS.

Figure 5G and S5I:

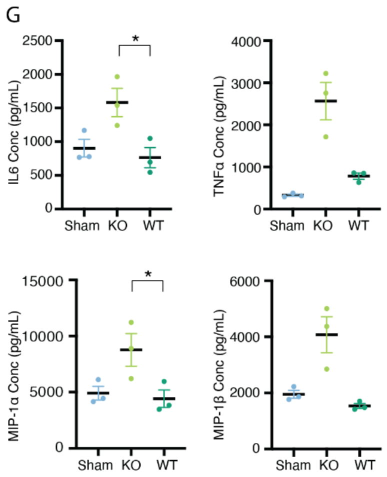

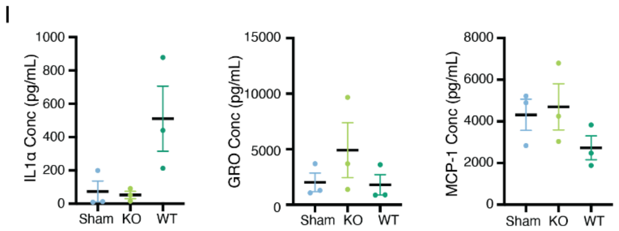

Concern 3: **“**Most of the figures are shown in PCs. Can the authors justify why they use PCs vs tSNEs, UMAP or diffusion map?”

Response: We thank the reviewer for raising this question, which gives us the opportunity to clarify our approach and improve the manuscript. We deliberately chose PCA as a vehicle for defining cell state changes within a subpopulation because it allows us to detect the largest signal or greatest source of variance within a population of cells and then compare PC scores as a proxy. This approach works for our data because we find that, as expected, in the *in vivo* response we see in innate immune populations, the presence of toxin does not elicit a wholly new response program, but rather we capture subtle and dynamic changes in each population across time points. PC score analysis also allows us to compare responses across multiple immune cell subtypes, whose gene profiles are not identical, but which are functionally related. Rather than simply comparing sets of genes that may not exactly match across cell subtypes, we are able to leverage PC ranking as a way to define the major signal within that group of cells, and then measure how it compares between conditions and across time points.

For defining cell types and cell subtypes, we defined groups by clustering and looking at expression of marker genes, and simply chose the visualization method that best illustrates what we see. Because the *in vivo* data includes some developmentally related cell types (both the major cell lineages and trophoblast subtypes), we found that PCA captured the relationship between these subtypes that helped us with annotations. However, in response to this comment, we have also updated the manuscript to include both PCA and tSNE anywhere we define cell types or cell subtypes.

Action: In response to this comment, we have added either the accompanying PCA or tSNE everywhere where we define multiple cell types or subtypes within a group of cells.

S2A, S2C, S2E, S5D:

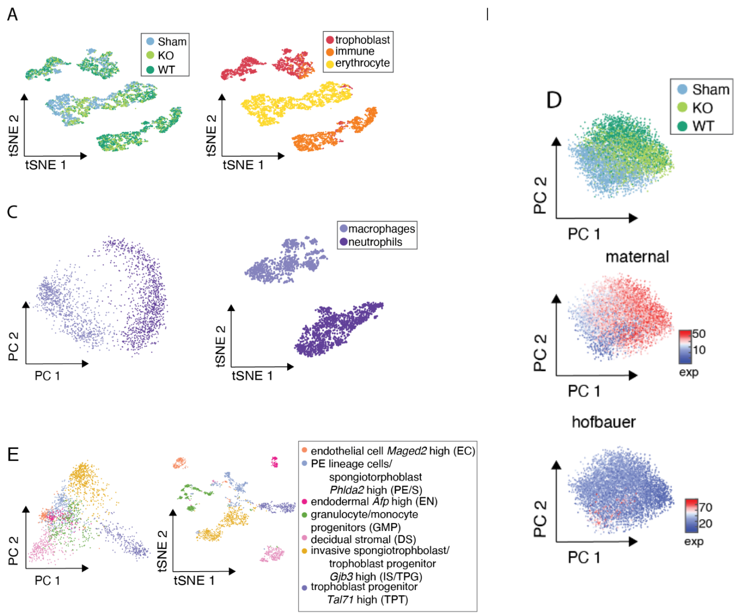

Concern 4: **"**Why do they use tSNE for human single-cell data and PC for mouse data?"

Response and Action: We thank the reviewer for raising this point, and we believe we have addressed much of our rationale for it in the above comment. We would like to highlight that we have updated Appendix Figures S2 and S5 in order to address this and improve clarity in the manuscript. In response to the above concern, we focused on our justification for use of PCA in defining and comparing cell states. It is important to further state that in this revision we have deployed both PCA and tSNE together with an independent clustering method to define and visualize cell types or subtypes. For the human macrophage subtypes, we found that PCA shows very little definition between subtypes, but there is biological justification for separating these two groups, and clustering separates these subtypes as well. The similarity between these groups in transcriptomic response could be an artifact of culturing the cells overnight, or a potentially important finding, but is out of the scope of the current manuscript.

Concern 5: **"**The authors need to explain how they annotate cell types and subtypes better. Which genes, gene signatures?"

Response: We thank the reviewer for pointing this out, and we agree that it is important to add details to the manuscript in order to make this aspect clearer. For lineage gene expression, we added a heatmap showing expression of independently curated genes that are highly expressed in each lineage (S2B) in addition to the differentially expressed genes between lineages shown in figure 2b. For subclusters within lineages, we manually annotated the identity of clusters based on identified markers. To illustrate our curated genes, we included heatmaps in figures S2D and S2F. For human placental subtypes, we updated the explanation of how we identified subtypes in the Methods section and included a table listing the genes we used to identify subtypes across samples in order to make our work as reproducible as possible for readers.

Action: We have added lineage marker heatmaps to Figure S2 (S2B, S2D, S2F):

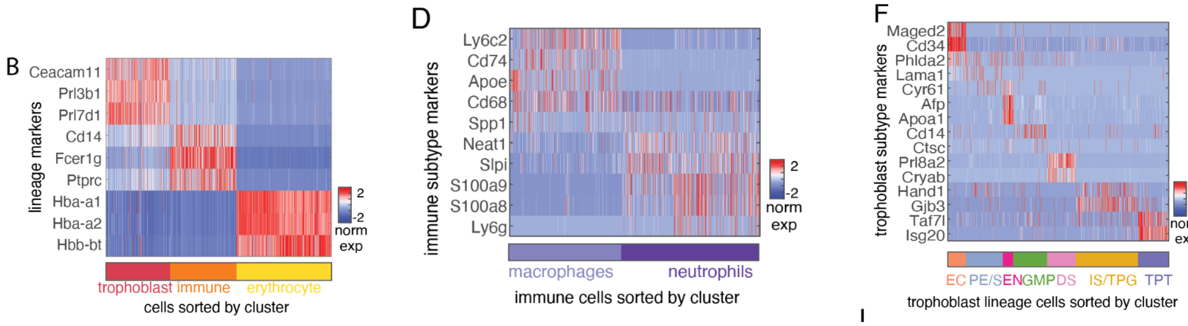

We have also updated the methods section which describes identification of human placental macrophage subtypes as well as Appendix Table S4, which includes the list of genes we used for subtype identification:

> **"Identification of *ex vivo* macrophage subtypes:** Cell type profiles were established based on previously characterized cell types in human placenta (Tetz, Aronoff, and Loch-Caruso 2015; Thomas et al. 2021). Specifically, multiple previously identified maternal macrophage subtypes were combined as were multiple stromal cell types. For one replicate, filtered cells were visualized with tSNE and clustered with hierarchical clustering. Maternal, Hofbauer and stromal clusters were identified by sum expression of combined genes from each of these profiles. To establish robust gene profiles for identification of the three major subtypes across samples, significantly upregulated genes unique to each of the three clusters were identified. Filtered cells from subsequent replicates were visualized with tSNE and clustered with hierarchical clustering. For each, normalized sum expression of uniquely expressed Hofbauer, maternal and stromal marker genes were visualized and cluster identity was assigned. If any cluster showed expression of stromal marker genes, it was eliminated from analysis and another round of tSNE and clustering was performed. This was repeated for each

replicate until the only clusters remaining showed expression of HB or maternal macrophage genes."

Appendix Table S4:

Hofbauer genes: *SPP1, TNFRSF4, SGK1, EMP3, EEF1A1, TNFRSF18, ACTG1, PLD3, LPL, PLIN2, JUN, IQGAP2, GPNMB, GADD45B, P2RX7, HMGA1, ETS2, CCDC50, DAB2, CBLB, SQSTM1, MATK, RGCC, OXR1, SLC16A10, NEURL3, TNFRSF12A, FNIP2, IL1RN, SNX8, CD84, HS3ST2, RASGEF1B, EEF1B2, CD9, KLF6, EGR2, DDIT3, CAST, SH3BGRL3, FMNL2, HSP90AB1, ACTB, PDGFA, MYOF, EPS8, RTN4, ACADVL, CD36, MT-ND3, IRF1, IFRD1, ARHGAP10, PLEKHO1, CCND1, GBP2, ANKRD28, HAVCR2, CD109, RCAN1, ANXA5, CD40, NANS, ST3GAL6, ANXA4, CEBPB, PARP14, SRFBP1, SCIN, ABCE1, ITGB5, OGFRL1, DDX21, YBX1, RBMS1, TMEM273,NMB, PPARG, GNG12, RPL10A, LACTB, C6orf48, CXCL16, SLCO4A1, PLA2G16, MT-CYB, ANKRD12, PIM3, WSB1, SEMA6B, EMP1, JAK1, CTSD, TNS3, CD276, PRR5, HPGDS, RPL22L1, CD86, MGAT1*
Maternal genes: *FCER1G, IL1B, TNFAIP6, EREG, SLC7A11, BASP1,SLC25A37, TXN, MGST1, C15orf48, CYBA, SERPINB2, ITGB8, CD48, PILRA, CXCL1, CSTB, AQP9, C1orf122, ADAMDEC1, FTH1, THBS1, MARCKS, CXCL2, CXCL3, IL7R, ANPEP, SERF2, HLA-DRB1, SERPINA1, SRGN, CLEC4E, HLA-DQA1, TSC22D1, MET, CYP1B1, B2M, MYO1G, IL36G, MT-CO1, SLC39A8, PLAC8, MT1E, CXCL5, GM2A, CCL3, RPL36AL, MSC, MT1X, IFI6, PTGS2, MMP14, CSTA, TNIP3, MAP3K20, SEC61G, NCF2, HLA-A, CREG1, ADA, LYZ, S100A9, CAPG, DNAJC3, ATP13A3, HLA-DRA, SERPINB9, CTSH, CARD16, TALDO1, MTRNR2L12, SLC7A7, EHD1, OAZ1, PLD1, MT1F, OST4, BRI3, CXCL8, DYNLL1, PLAUR, S100A8, CYB561A3, IRAK3, MT1M, IL19, TIMP1, IL2RA, LYN, ARPC5, SLAMF7, PTGIR, ATP5F1E, IL10, IDO1, NAMPT, CYTIP, CES1, S100A11, S100A10*
Stromal genes: *CYR61, IL1RL1, SERPINE2, LMCD1, APOD, C7, CXCL14, SPARC, CTGF, IGFBP3, CALD1, RARRES2, EGFL6, GPC3, CRYAB, KRT18, LUM, DCN, DLK1, CDH11, COL1A1, TIMP3, FBLN1, COL6A1, COL6A2, SERPINE1, SERPINF1, COL3A1, SRPX, PTGDS, CNN3, FSTL1, WNT2, SOD3, AKAP12, AEBP1, HGF, DKK1, RSPO3, COL4A2, ADAM12, IGF2, TAGLN, COL4A1, FST, PLOD2, SELENOM, TFPI2, PCOLCE2, SLC22A3, CLMP, COL14A1, MGP, MFAP4, ERRFI1, LARP6, ID3, C1R, PCOLCE, CCDC80, TGFBI, COL1A2, EXT1, FBLN2, FRMD6, ABI3BP, CAVIN1, FHL2, IGFBP5, MSX1, OSMR, PLAC9, TBX3, FGF7, DKK3, MAP1B, VGLL3, UCK2, MYL9, EPAS1, CLU, COL5A1, EFEMP1, FHL1, RRAS2, SPTBN1, IGFBP2, COL15A1, MYC, MEST, MMP23B, MMP2, TEAD1, ENPP1, PRRX1, EGFR, SEMA3B, BDKRB1, DPT, IL13RA2*

Concern 6: "The authors need to define how Y-chromosome genes are calculated."

Response and action: We thank the reviewer for highlighting this important detail and took the opportunity to clarify our approach. We revised the relevant part of the methods section:

"Macrophages from male fetuses were identified on the basis of sum log Y chromosome expression above a threshold (0 in mouse and 0.4 in human). Differentially expressed genes were defined between male fetal macrophages and all other cells, and any significant genes were used for hierarchical clustering of all

cells into two groups. The group with an overrepresentation of male fetal macrophages was defined as fetal. To measure fetal correlation as a confirmation of identity of non-Y chromosome-expressing macrophages assigned to the fetal cluster, expression of each cell was compared with the average expression of the same set of differentially expressed genes across all confirmed male fetal macrophages."

Concern 7: "Can the authors explain cell type-specific transcriptional changes in each subtype of trophoblast, immune cells in more detail?"

Response: We thank the reviewer for this comment, which has prompted us to perform and include additional analyses on other lineages. The immune cell lineage captured the two main subtypes analyzed in the rest of the paper. We have added analysis on the transcriptional changes induced in other cell types in response to infection, although deeper analysis into these changes and the impact of those cell type-specific changes is outside of the scope of this manuscript.

Action: We have made the following modifications to the manuscript in response to this comment: We have added cell-type specific analysis on trophoblast lineage subtypes between sham- and GBS-infected cells in Appendix figure S2 and updated corresponding text. The erythrocyte lineage cells constitute one group of cells (see PCA below), but we have added analysis between conditions on all erythrocyte lineage cells.

Appendix Figure S2H-I:

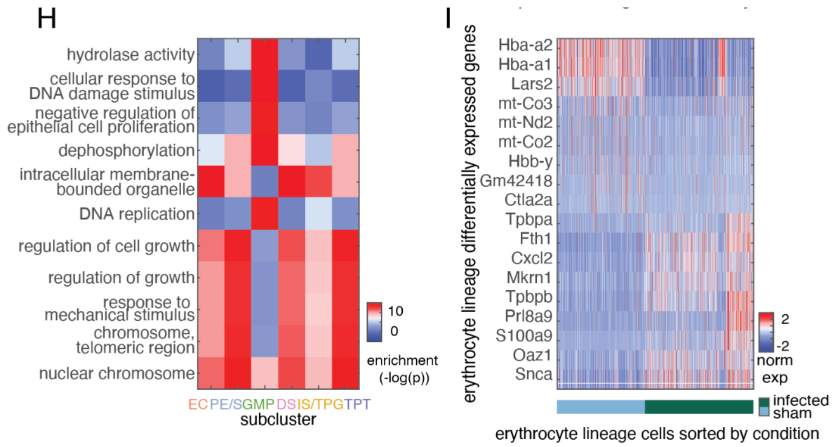

"We asked whether identified cell lineage subclusters also underwent transcriptional changes in response to infection even if those changes aren't reflected on a whole-lineage level. To answer this we identified differentially expressed genes between cells from GBS- and Sham-infected mice within each subcluster and measured the functional enrichment of the infection-specific genes within each subtype using Gene Ontology (GO) (Appendix Fig S2H). Cells annotated to the Endodermal *Afp* High subcluster showed no significantly upregulated genes between conditions and were

therefore excluded from functional enrichment analysis. The erythrocyte lineage did not include discrete subtypes, but analysis of differentially expressed genes between conditions revealed an enrichment in inflammatory expression in GBS-infected erythrocyte lineage cells (Appendix Fig S2I). Immune lineage subtype changes will be discussed in subsequent analyses."

Concern 8: "In Figure 1, texts in scale bars are too small."

Response and action: We thank the reviewer for pointing this out, so that we can improve clarity for the reader. We have increased the size of the scale bar text in Figure 1 and Appendix Figure S1 to improve visibility:

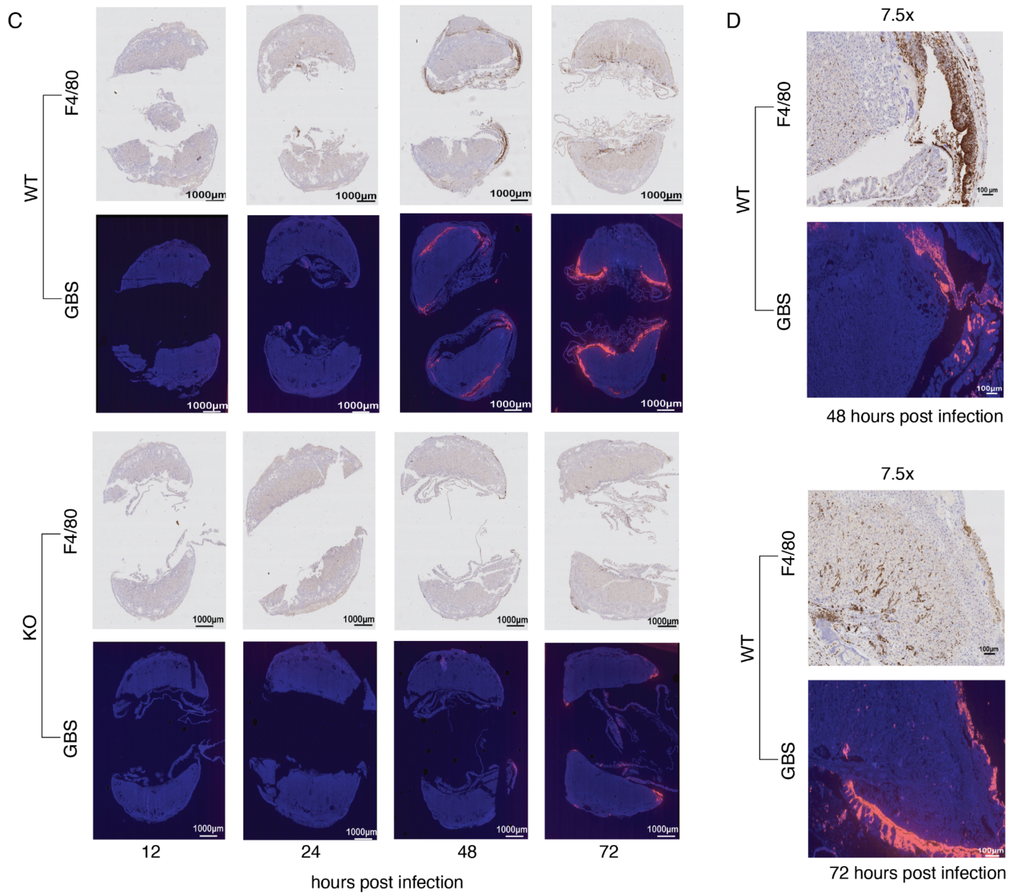

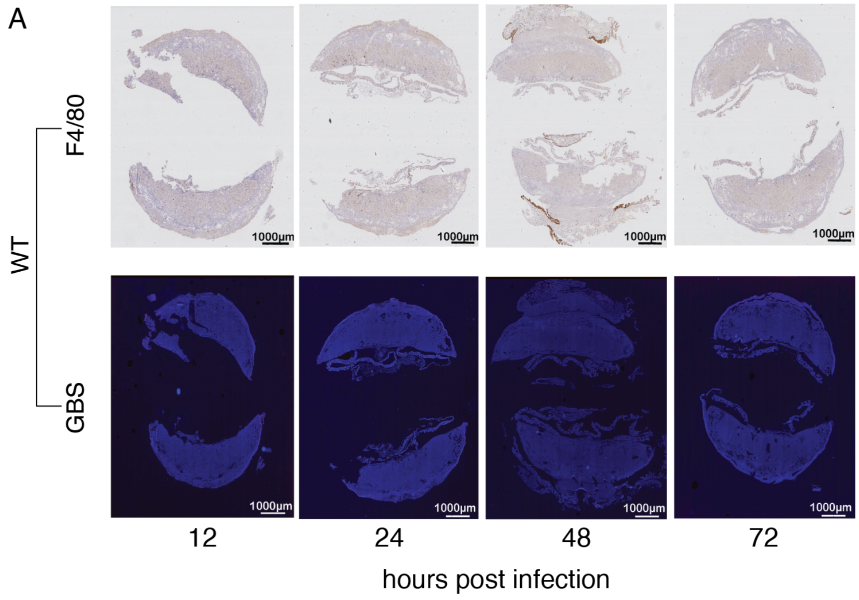

Concern 9: "Figure 2d, several dots are missing in the cell type labels"

Response: We thank the reviewer for highlighting this apparent error so we can clarify it. Dots were not missing, but there are two clusters that each represent two different, but related, subtypes. Because clustering did not distinguish them, we annotated them as such. In order to clarify and prevent misunderstanding, we have indented the second line of the names of each of those and changed an "&" to a "/" so that it matches the key under the corresponding heatmap.

Action: We changed the format of the legend in Appendix figure S2E so that the clusters are more clear:

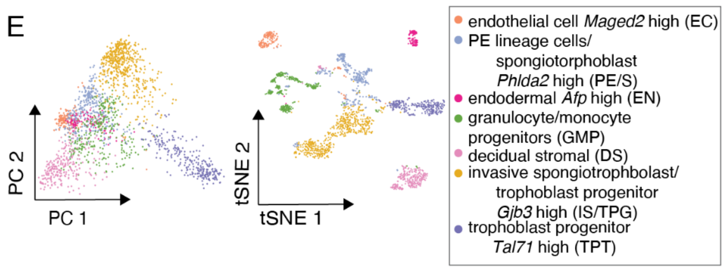

Concern 10: "Overall, figures need to be better scaled and organized."

Response and Action: We thank the reviewer for this comment, and acknowledge that our figures benefited from organization. We have attempted to do so for the revision after

incorporating all of the changes to the figures. Please see all updated figures in the manuscript.

Reviewer #2:

Summary: "In this study, the authors sought to investigate the placental cellular mechanisms implicated in ascending GBS infection during pregnancy, and the role of β-hemolysin/cytolysin (β-h/c) in such a process. The authors performed time-course sampling of placentas from mice infected with wild type (WT) or β-h/c-deficient (KO) GBS at 12 - 72 h post-infection for single-cell RNA-sequencing (scRNA-seq) as well as histological analyses. Moreover, placental macrophages from women undergoing term cesarean section were utilized for ex vivo assays followed by scRNA-seq. First, the authors demonstrated that the placental GBS burden is reduced in KO-infected dams; nevertheless, infection with either strain resulted in increase co-localization of GBS and macrophage marker expression in the placenta. Bioinformatics analysis of scRNA-seq data implicated placental immune cells as the primary population affected by GBS infection, with neutrophils and macrophages being the primary cell types involved. Notably, the authors observed that both neutrophils and macrophages from KO-infected mice have reduced inflammatory gene expression in early infection phases (prior to 48 h) compared to those cells from WT-infected mice; however, in late infection, cells from KO-infected mice show greater expression of inflammatory genes. Such macrophage responses primarily involved maternal cells in both infection conditions; yet, WT-infected mice also showed a fetal macrophage response in late infection (72 h). As validation, the authors exposed human placental macrophages to WT or KO GBS ex vivo and found that KO-infected macrophages displayed a greater inflammatory expression profile compared to the WT-infected cells. The authors propose a model whereby β-h/c contributes to poor perinatal outcomes due to GBS infection by subverting the inflammatory responses of placental neutrophils and macrophages, thereby preventing appropriate immune activation and pathogen clearance."

Response: We are grateful for the time and effort spent by the reviewer on our manuscript. Their concerns and comments regarding clarity, thoroughness of discussion and contextualizing our findings appropriately led us to improve our presentation of the results. Specifically, these comments have helped us to revise our manuscript in terms of better addressing phenotypic differences between treated groups (Concern 1), expanding implications in discussion (Concern 2), expanding the analysis and discussion of uniquely of the unique program induced by WT GBS (Concern 3), improving the presentation of results in the discussion section (Concern 4), and better contextualizing our study in the introduction section (Concern 5). Overall, we believe we have addressed the concerns of the reviewer.

Concern 1: "Overall, this well-designed and straightforward study addresses important mechanisms of ascending microbial infection during pregnancy. Specific comments are as follows: What are the pregnancy outcomes of GBS infection using the WT and KO

strains? Do these mice undergo preterm birth, and are there differences between strains in terms of preterm birth rate, neonatal mortality, etc.? Given that the peak inflammatory response seems to occur at 72 h (approximate to 16.5 days post coitum), the occurrence of preterm birth shortly after this point would be consistent."

Response: The reviewer is correct in pointing out that in our design of the experiment we do not quantitatively report on systemic outcomes of the infected mice. Instead we designed this study to build on the very well-characterized phenotypic difference between these strains previously defined by members of our team (Randis et al, 2014). In that work, Randis et al found that intrauterine fetal demise and preterm birth were observed in more than half of the cohort infected with WT GBS, where none was observed in the cohort infected with the same β-h/c knockout strain we used, suggesting that it's most likely a direct result of the presence of the toxin. They also observed significantly more placental inflammation and disruption of maternal-fetal barriers measured by histological examination of tissues as well as fetal infection in mice colonized with the WT GBS strain. The design of this study does not allow robust, quantitative comparison of pregnancy outcomes because it was primarily designed as a follow up study on the established model, to collect single cell data from these samples in order to understand the transcriptomic changes that accompany the observed tissue-level phenotype.

Action: We have better described these findings in the relevant parts of the text to emphasize the results on which we based our study design:

> "Previous work has already shown that using this same model of ascending infection, intrauterine fetal demise and preterm birth were observed only in the cohort infected with WT GBS and there was significantly more placental inflammation and disruption of maternal-fetal barriers measured by histological analysis of infected tissue than in mice infected with β-h/c KO GBS (Randis et al., 2014). We built on these results by first recapitulating previously-defined phenotypic differences in the placental response to these two GBS strains using tissue from the same mice processed for scRNA-Seq (Table 1). To do so, we used two readouts: We measured bacterial burden in one placenta per mouse from each condition and found that at each time point, bacterial load, measured by CFU/gram of placenta, was higher in WT-infected than KO-infected placentas, consistent with previous findings (Randis et al., 2014) (Fig 1B, see methods). We also found that the bacterial load increased within each condition at every time point relative to earlier samples, suggesting that both time post infection and presence of β-h/c dictate the extent of bacterial burden in the placenta (Fig 1B)."

Concern 2: "Could the authors expand the discussion of their findings related to fetal macrophage activation with WT infection? Is the lack of such a response in the KO infection model due to the preservation of anatomical barriers, or is it driven by the changes in gene expression/cellular function? Differences in the fetal inflammatory response and fetal bacterial load between strains would also be interesting, yet this may be outside the scope of the current study."

Response: We thank the reviewers for this comment, which has prompted us to expand on this topic in the Discussion. This work has led to many new questions regarding the differential responses of fetal and maternal macrophages, which we are actually following up on in future work. However, we were not able to recover sufficient fetal macrophages to perform significant additional analysis.

Action: We have expanded on this topic in the relevant section in the Discussion:

> We consider a number of ways to explain this apparent contradiction: First, we distinguish between fetal and maternal macrophage inflammation in the placenta. While a reduction in maternal macrophage inflammation was correlated with worse outcomes, we also observed a concurrent activation of fetal macrophages, which, we suggest, could result from GBS overwhelming the maternal response. Given the colonization of GBS along fetal membranes starting at 12 hours following infection (Figure 1c-d), it is unlikely that anatomical compartmentalization prevents activation of the fetal macrophages until 72h post infection. Instead, it is more likely that the functional response of maternal immune cells eliminates the need for activation of fetal immune cells in the placenta, which in turn preserves their own functional abilities. Of course increased tissue damage can play a part, but if anything that might elicit a hypoxia-dependent wound healing response, which contributes to suppression. This activation could prevent fetal macrophages from executing normal functions important for fetal support and, together with barrier disruption, interfere with success of the pregnancy. Also, aberrant inflammatory skew can induce premature labor by mimicking the inflammatory milieu that triggers labor under normal circumstances (Erlebacher, 2013; Yao et al., 2019). We suggest that inflammatory skew of fetal macrophages can lead to this phenotype. We do not see this difference in response in human placental fetal macrophages *ex vivo* because tissue context is critically important in maintaining a tissue resident macrophage phenotype.

Concern 3: "The observation in Figure 5 that WT GBS drives a different set of genes is interesting and would benefit from additional analysis and/or discussion. What sort of pathways/functions are this gene set implicated in? Such analysis could help complement the final discussion paragraph in which the authors mention specific genes such as BHLHE40."

Response and action: We thank the reviewer for raising this point. In order to address this we have performed an additional analysis in Figure 5 looking at the effect of an important upregulated transcription factor, *BHLHE40,* and we have also expanded the discussion section about the role of inflammatory regulation in GBS infection.

H

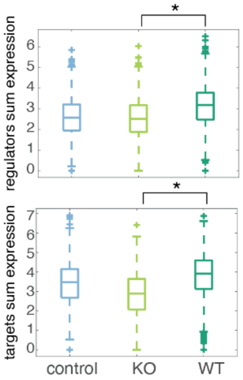

“Our work has highlighted a potential route of GBS disease modulation based on WT GBS-induced gene expression (Figure 5). Some of the upregulated genes in response to WT GBS infection in maternal macrophages have functions in inflammatory regulation, including *IER3, TNFAIP3,* and *BHLHE40 (Arlt and Schäfer, 2011; Cook et al., 2020; Coornaert et al., 2009; Parvatiyar and Harhaj, 2011)*. *BHLHE40* is a transcription factor involved in different cellular processes in a variety of tissues including circadian rhythm regulation and differentiation (Boudjelal et al., 1997; Honma et al., 2002; Shen et al., 2002; Yun et al., 2002). Recent work has implicated this gene in inflammatory regulation in different physiologic contexts (Cook et al., 2020; Jarjour et al., 2019), and we hypothesize that because of its regulatory role in inflammation, targeting *BHLHE40* could limit host inflammatory suppression leading ultimately to better phenotypic outcomes. Consistent with our *in vivo* findings, both the KO-infected and WT-infected conditions show significant inflammatory activation, which is reasonable given that the conserved innate response has evolved to respond to diverse stimuli and the presence or absence of a single toxin should not impact the overall response. The *ex vivo* analysis, however, is designed in a way that can provide some insight into the impact of the presence of β-h/c. It could be the case that these regulatory genes are induced by a stress response caused by increased greater cell lysis due to β-h/c, in which case regulation would be an indirect consequence of β-h/c, but which allows increased colonization and survival for WT GBS. This study lays the groundwork for future investigation into immune regulation better in this setting, and will address precisely how this regulation impacts phenotype in an *in vivo* context. Ultimately, this has important implications for clinical intervention through modulation of this and other inflammatory regulators for improved bacterial control, while limiting the impact on normal host cell function during pregnancy.”

Concern 4: “The first paragraphs of the discussion are a little bit results-heavy, perhaps the key findings can be summarized in one short paragraph without too much repetition of results or methods.”

Response: We thank the reviewer for this suggestion that has improved the clarity of our discussion section, and we are happy to modify the text accordingly.

Action: In this revision we have compressed the results summary in the first two paragraphs of the discussion section into one with fewer details as follows:

> "In this work, we defined the placental cell state dynamics in response to GBS with or without expression of the toxin β-h/c. We first recapitulated the difference in tissue-level phenotype between the response to a β-h/c KO and isogenic WT GBS strain (Randis et al., 2014), using a mouse model of ascending infection. We found that WT GBS induced a more severe phenotype measured by histological analysis and bacterial burden than the β-h/c KO (consistent with prior studies). We analyzed corresponding scRNA-Seq data to understand the cell state changes that accompanied this phenotypic progression. PC score analysis revealed GBS induction of an IL-1 family inflammatory response in innate immune cell populations, but different response dynamics depending on the presence of β-h/c. In the first stage after infection (up to 48h), immune cells from WT-infected mice expressed relatively higher inflammatory response genes than those from β-h/c KO-infected mice. In the later stage however, (48-72h), we observed relatively less inflammatory expression in cells from the WT- compared to the β-h/c KO-infected mice, concurrent with the presentation of severe phenotype in WT-infected mice. We found that fetal macrophages were only activated when infected with WT GBS at 72h post infection. We validated the relatively lower expression of inflammatory response genes induced by WT- compared to β-h/c KO-GBS in maternal macrophages and neutrophils in an *ex vivo* infection of human placental macrophages."

Concern 5: "Introduction: The authors should consider including several additional studies showing the involvement of macrophages at the maternal-fetal interface in pregnancy maintenance as well as adverse pregnancy outcomes."

Response: We thank the reviewer for this suggestion because there is much important literature on the topic, which is relevant to our work, and we absolutely agree that these should be included more of these studies.

Action: We have updated the introduction paragraph which deals with the role of macrophages in healthy and pathological pregnancy and cited several more studies:

> "Macrophages are plastic and heterogeneous cells, in which polarization state and function can differ depending on their environment and ontogeny (Locati et al., 2020). Macrophages in the placenta, which can be maternal or fetal in origin (Erlebacher, 2013; Yang et al., 2019), mediate different functions at different stages of pregnancy including phagocytosis of apoptotic cells, facilitation of invasion and remodeling and cytokine secretion, all of which are important to support a successful pregnancy (Ander et al., 2019; Erlebacher, 2013; Liu et al., 2017; Mezouar et al., 2021; Reyes and Golos, 2018; Yao et al., 2019). Throughout pregnancy, placental macrophages maintain a balance of pro- and anti-inflammatory polarization states, and disruption of this balance has been associated with pathological outcomes of pregnancy including miscarriage,

> preeclampsia and preterm birth (Erlebacher, 2013; Gonzalez et al., 2011; Guenther et al., 2012; Mezouar et al., 2021; Schonkeren et al., 2011; Yang et al., 2019; Yao et al., 2019). Macrophages have also been implicated in the response to GBS infection in the placenta (Randis et al., 2014; Sutton et al., 2019), but their precise role in mediating these pathological outcomes and maintaining a healthy pregnancy is not known."

Reviewer #3:

Summary: "This study conceptually advances our understanding on how vertical transmission of Group B Streptococcus (GBS) modulate immune cells function and response in the mouse and human placenta. GBS is commonly found in the vagina and can pass from pregnant mother to her fetus during labour. GBS can also ascend from the vagina to upper reproductive tract during pregnancy and can cause severe consequences. This study utilized the murine vaginal colonization model to study ascending infection phenotype over time. Specifically, the authors suggested, in part, that beta-hemolysin/cytolysin as the major GBS virulence factor responsible for switch in inflammatory signalling in placental macrophages and neutrophils. The authors take advantage of single-cell genomics profiling on infected mouse placentas and human placental macrophages. However, there are several points and validation that must be adequately addressed before the paper can be accepted."

Response: The reviewer clearly dedicated considerable time and effort to evaluate our work and we appreciate this very much. In this revision, we believe that we have addressed all of the reviewer's highly constructive comments. The manuscript has been greatly improved as a consequence. Broadly, the concerns of the reviewer fall into the following classes, which we address and detail below in the enumerated concerns:

Clarity of data presentation, language and technical clarification: add details to methods and supplementary material for reproducibility (1, 18, 26), improve data visualization and figure layout (4, 14, 19), improve language for clarity (9), address errors in manuscript (2, 17), and add technical details to methods (16, 21, 22, 24, 28, 29).

Improvement in analytic approach, validations and controls: refine and validate single cell analysis (6, 23, 25), make conclusions more robust (5, 27), validate experimentally and technical conclusions (7, 10, 12).

Improvement in conclusions and expansion of interpretation: reconsideration of conclusions (3, 8), additional discussion (20, 30), clarification of caveats (11, 13).

Concern 1: “There are many details missing in the methods section regarding the analysis of scRNA-seq data. In order to make this study reproducible and clearer for the reader, the authors should include: how the clusters were obtained, method used to get the marker genes/differentially expressed, number of cells analysed, QC thresholds used for the ex vivo macrophage analysis, among any other detail that the authors consider it's important for future reproducibility.”

Response and action: We thank the reviewer for raising this very important issue. We have added the section below entitled “Lineage and cell type identification,” which addresses the way clusters as well as marker gene and differentially expressed genes were obtained. The number of cells in each analysis as well as other metrics have been added to figure legends. Finally, we added a section on *ex vivo* data processing and filtering. Originally we had not included a separate section since the *in vivo* and *ex vivo* thresholds were the same, but for the sake of clarity we have a dedicated section for the *ex vivo* data.

> **“Lineage and cell type identification:**
>
> Lineage clusters were generated through hierarchical clustering and identified primarily through expression of marker genes independently from the literature. Data from each time point was clustered independently, and while the three main lineages separated for most time points, at 48h post infection, granulocytes were so inflammatory that they separated out separately for macrophages. In order to maintain consistent cell clustering across time points for comparison, four clusters were isolated from cells at 48h post infection, and the neutrophil and macrophage clusters were combined to form the immune cell lineage. Cluster identity was confirmed based on expression of manually curated genes (Figure s2b) and differentially expressed genes identified by Ranksum test ($p < 0.0001$) between clusters (Figure 2b). Within the immune and trophoblast lineages, cells were sub clustered and identities were assigned based on expression of manually curated genes from the literature unique to each subcluster (Figures s2c,e).”
>
> **“Single cell processing and filtering for human placental macrophages:**
>
> Cell Ranger pipeline (V.3.0.0) was used for demultiplexing, mapping to the human genome (GRCh38) and counting umis for individual cells. Cells were filtered out if they had less than 500 unique molecular identifiers (UMIs), and if more than 20% or 15% of their transcripts were ribosomal or mitochondrial genes, respectively. We normalized the expression to transcripts per median (TPM) and log transformed the expression.Each placenta was analyzed separately.”

Concern 2: “There are inconsistencies in the values plotted in Figure 1b, which do not match those in table 1. In addition, it is not possible to make a reliable comparison in Figure 1b using n=1 to see if the CFU/gram of placenta is decreased in KO-infected placentas. Even if the presence of GBS seems to be reduced in Fig 1c-d. Bacteria

burden in CFU/g counts does not match counts in Table 1 where CFU/g is indicated in column 3. For example, at time-point 72 hr, in Table 1 KO4 shows 9x10^9CFU/g and WT4 shows 1x10^9 CFU/g. Whereas the scatter plot in Figure 1b at time-point 72 hr shows that WT 10^11 CFU/g range. The discrepancy is at 2-log fold change and the conclusion will need to be re-addressed if this is true."

Response: We are grateful that the reviewer caught this error. The value in the table for 72h was copied wrong, and we've updated it to the correct value, which is reflected in 1B. We have an image of the bacterial plate from which we calculated the CFU and we also have the recorded weight of the placenta from which we calculated CFU/g, which we are happy to provide if requested. It is true that n=1 placenta for quantitative CFU/g analysis is not enough to conclude that there are differences in bacterial burden, but the differences in this model elicited by these two strains have already been robustly quantified and published by members of our group (Randis et al, 2014). This study follows up on that model by measuring transcriptomic changes that accompany tissue-level differences, and we showed tissue-level phenotypic differences in order to contextualize our transcriptomic data in what has already been shown.

Action: We have updated the table to reflect the correct values.

Appendix Table S1

mice infected E13	t	CFU/g	histology	Vaginal CFU/swab	Culture result of placentas for scRNA-seq	included in scRNA-Seq analysis
Sham 1	12h	-	y	-	+	y
Sham 2	24h	-	y	-	+	y
Sham 3	48h	-	y	-	+	y
Sham 4	72h	-	y	-	+	y
KO 1	12h	-	y	$8x10^4$	+	y
KO 2	24h	$22x10^5$	y	$6x10^3$	-	y
KO 3	48h	$24x10^7$	y	$2x10^8$	+	y
KO 4	72h	$9x10^9$	y	$1x10^2$	+	y
WT 1	12h	$10x10^6$	y	$3x10^6$	+	y
WT 2	12h	$4x10^5$	n	$1.2x10^5$	+/-	y
WT 3	12h	-	n	$3x10^3$	-	n

WT 4	24h	$23x10^6$	y	$8x10^5$	+	y
WT 5	24h	$4x10^8$	n	$2x10^5$	+	y
WT 6	24h	-	n	-	-	n
WT 7	48h	$17x10^8$	y	$2x10^8$	+	y
WT 8	48h	$5x10^9$	n	$6x10^4$	+	y
WT 9	48h	-	n	$4x10^5$	-	n
WT 10	72h	$1x10^{11}$	y	$1x10^7$	+	y
WT 11	72h	-	n	$6x10^6$	-	n
WT 12	72h	N/A	N/A	N/A	N/A	N/A

Concern 3: "The authors suggest there is a gradient of erythrocyte differentiation revealed by the PC1 scores in Figure S2c. A more detailed analysis is needed to claim this, as it could only be the stress elicited by the infection, and not a differentiation process."

Response: We thank the reviewer for this comment, which has prompted us to reexamine our conclusion and modify our claim. We do see heterogeneous expression of a number of erythrocyte lineage markers, including *Alas2,* but it is entirely possible that infection induced a stress response in this population and that PCA reflects that response. It is also possible that infection causes a shift in the representation of erythrocyte lineage cells at different stages because loss of erythrocytes in infection induces erythropoiesis.

Action: In response to this comment, we have changed the analysis on the erythrocyte lineage as well as the claims we make in the text:

Appendix figure S2I:

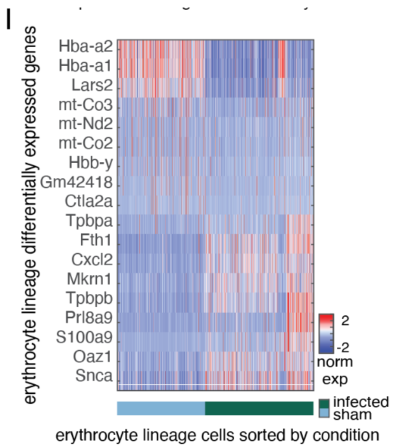

> "The erythrocyte lineage did not include discrete subtypes, but analysis of differentially expressed genes between conditions revealed an enrichment in inflammatory expression in GBS-infected erythrocyte lineage cells (Appendix Fig S2I)."

Concern 4: "I would suggest to refine the scRNA-seq analysis. The major cell types are very constrained in the projection to be able to see all the differences between and within them mentioned by the authors. For example, the fact that trophoblast and immune cells are overlapping suggest that using PCA as their dimensionality reduction method is barely enough to differentiate between quite different cell types. Therefore, using this projection to look into sub-type differences is quite bold (e.g. Figure 2d where granulocyte/monocyte progenitors are clustered with trophoblasts; the impossibility to recognise the 7 sub-types reported by the authors in Figure 2d; or the presence of only two immune cell types in Figure 2c). I would suggest trying a different dimensionality reduction algorithm (e.g. t-SNE or UMAP) to test if they can obtain a better resolution."

Response: We thank the reviewer for this comment, which raises an issue we have grappled with in analyzing these data. In order to address this and improve transparency and clarity, we have included both PCA and tSNE between supplemental and main figures anywhere where we identify cell types. In terms of the relationship between immune cells and trophoblast lineage cells, we found that the fetal immune cells are positioned between immune cells of maternal origin and other fetal cell types, and therefore there is a less discrete break between these lineage clusters. In general, we found that PCA recovers differentiation and lineage relationships well, for the main cell lineages, as well as within trophoblast subtypes, and for that reason we chose to visualize them with PCA. We also note that all cells were independently clustered and annotated manually, and that PCA and tSNE act only as the visualization method for these clusters. Having said that, we appreciate this comment and were very happy to include tSNE for cell type or subtype identification.

Action: We have added tSNE visualization with the same clustering analysis to relevant appendix figures:

S2A, S2C, S2E, S5D:

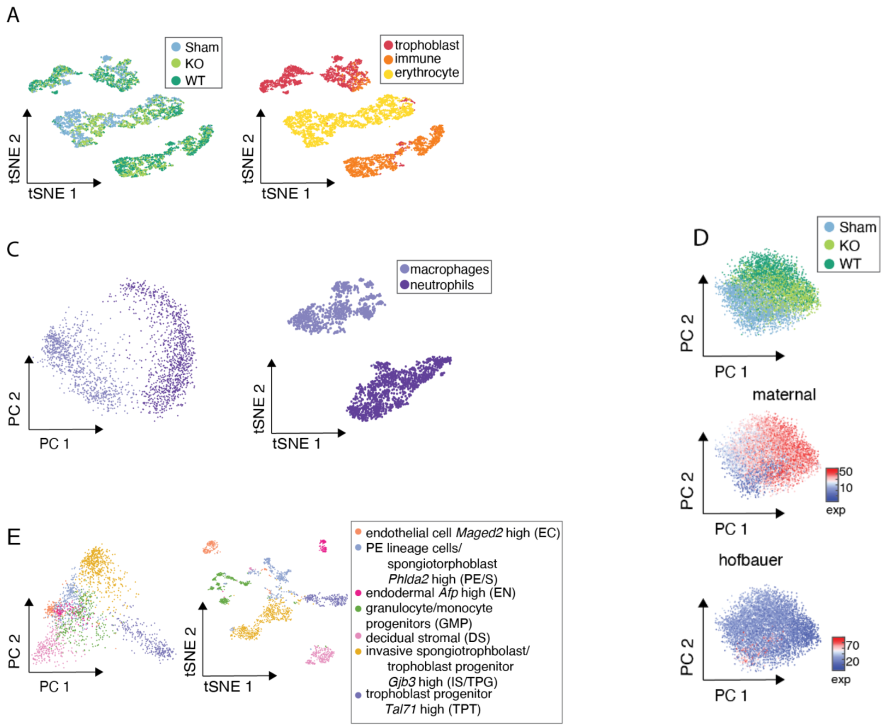

Concern 5: "With Figure 2e-f the authors claim that "This confirms that innate immune cells undergo transcriptional changes in response to infection". Even though, the high proportion of immune cells in the analysed tissue is directly linked to the infection (i.e. infection = more immune cell recruitment), the imbalance in the dataset (<5% of cells coming from Sham controls) makes difficult to take this comparison as a "confirmation" of significant transcriptional changes in innate immune cells. I have no doubt this might be the case, but the authors cannot assure this with the evidence provided. If the authors want to demonstrate this conclusion, I would suggest them to sequence more innate immune cells from uninfected controls. As the presence of these cells in the healthy tissue seems to be very low, I can suggest to do an enrichment of immune cells using FACS or MACS. The authors can include this new data into the current dataset and then perform more reliable comparisons."

Response and action: We thank the reviewer for raising this important concern. We attempted to generate more data during this revision process by repeating the experiment. When we added just the cells from sham-infected mice to our existing data, the batch effect between the experiments was so great that correlations between different sham-infected cells were much lower than in the existing analysis.

We decided not to enrich by MACS or FACS because this would only increase the batch effect between experiments. In order to bolster our chances of adding useful data to the manuscript, we also infected mice when we repeated this experiment, in order to enable a new comparison to validate this analysis, but as described in the methods, not every infected mouse is culture positive, and those mice are excluded from analysis. Unfortunately the mouse infected for 48 hours was not culture positive, so we were not able to perform the same analysis on that data to add to the manuscript.

At the same time, we want to highlight that in addition to the correlation between cells from treated and untreated mice, we actually try to highlight exactly the point that the reviewer is raising: In addition to the shift in correlations within cell lineages between sham- and GBS-infected mice, we also attempted to show a shift in the composition of cells per condition in order to show that there is a large influx of immune cells, perhaps as expected, in the infected mice, and we dedicate the bulk of the paper to analysis of those cells. Originally we had illustrated this as the composition of cells from mice in different conditions per cell subtype lineage, but for clarity and in response to this reviewer comment, we have moved that analysis to the accompanying appendix figure (S2G) and changed the analysis we present in the main figure to the composition of lineage per condition (see below). In response to this important point, we have also added the caveat the reviewer raises to the main text (see below):

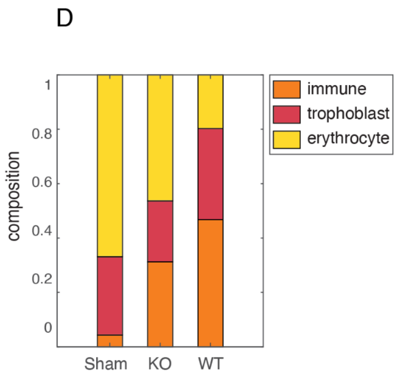

Figure 2D:

> "To determine which cell lineage underwent the greatest transcriptomic changes due to infection, we compared cells within each lineage from sham- and GBS-infected mice. For each lineage, we calculated the Pearson's correlation coefficient between every pair of cells from sham-infected mice. We then calculated the correlation between each pair of cells from sham- and GBS-infected mice and compared the distributions of correlations. We found that the erythrocyte and trophoblast lineages showed similar distributions of pairwise correlations between cells from sham- and GBS-infected mice, but immune lineage cells from GBS-infected mice were less correlated with equivalent cells from sham-infected

mice (Fig 2C). The relative composition of cell lineage types present in each condition shows that, as expected, infected mice have an influx of immune lineage cells (Fig 2D, Appendix figure S2G). We note that immune cells from sham-infected mice are not abundant at this time point, but given the large influx of immune cells in the infected mice, it is plausible that these cells are least correlated with placental immune cells present at steady state. On the basis of this shift in the immune cell population in infected mice, we focused our subsequent analysis on characterizing these responses."

Concern 6: “The authors found that many genes contributing to PC2 are related to an IL-1-family inflammatory response, Figure 3d. Then, the authors mention "We were therefore surprised to observe that, based on PC 2 scores, the KO-infected cells exhibited higher levels of inflammatory gene expression (Figure 3c)". The authors must remember that a PC is the linear combination of many variables (i.e. the expression of different genes). Even if there is a correlation between inflammation and the PC2, the authors cannot interpret PC2 solely as inflammation nor use the PC values to measure differences in inflammation as done in the quoted text, in Figure 3c and Figure 3e. The GO This is also done in Figure 4e, where they label the Y-axis "Inflammatory gene expression", when they are actually plotting PC1 scores. This is highly misleading, and also conceptually wrong, so it must be corrected. Other figures with this approach are Figure 4f, S4e and 5d. As one of the main results of the authors is that β-h/c KO-infected GBS induce higher IL-1 family inflammatory response, meaning that WT GBS might have an anti-inflammatory effect, the authors should change this approach of doing "PC score analysis". The authors can measure the inflammatory/anti-inflammatory response directly instead of using ambiguous PC scores to try to support their conclusions.”

Response: We thank the reviewer for raising this point, which addresses our central approach to analysis in this manuscript, and provides the opportunity for us to clarify and refine this approach. First, we’d like to address the criticism of the use of PC score comparison throughout the paper. We compare subtle transcriptomic shifts in different cell populations across conditions and time points. Working with these data extensively, we have learned that baseline inflammatory changes in the placenta with each day of pregnancy make it difficult to distinguish inflammatory activation from baseline temporal changes. Also, while these innate immune cell populations are coordinated in their responses, the precise genes expressed in each differ somewhat, so for comparative purposes, we find that it is much more robust to compare by common enriched functions and define the set of genes from within each individual cell type and subtype as relevant. Finally, the absence of β-h/c does not induce an entirely different response in immune cells, which is expected given that the many other toxins and bacterial residues are recognized by innate immune cells and the response we observe is conserved across many different antigenic stimuli. Therefore, we found that the best way to compare systematically and to capture subtle shifts in

the dynamics was by asking first what is the greatest source of variance in the data within each subpopulation we examine, and then how does that compare across conditions and time points. The reviewer is absolutely right that a PC is the linear combination of many variables and that is precisely why we use this approach: with these particular data we found it too limiting for most subpopulations just to take a curated set of genes and measure its expression across conditions for all of the reasons enumerated here. Instead, we define the response based on what a PC in each of these populations captures and then characterize that by the genes it includes and the functional enrichment of that set of genes.

Having explained the rationale for our approach, we would also like to say that the reviewer is correct and this analytic framework we developed and tailored to these data requires robust validation. Therefore, in every instance of PC score analysis, we have added the following analyses: we measure the difference in sum expression between condition of PC contributing genes and test for significance, we add a statistical test for every individual PC contributing gene in the PC score heat maps themselves and a supplemental table with these values, we also test for differentially expressed genes between conditions to validate higher inflammatory expression in the relevant condition in a more direct way.

Specific details of these changes are delineated here:

We have modified the text explaining Figure 3 analysis, the first time we present the PC score analysis.

> "We next asked what the variance captured by PC 2 corresponds to and found that PC 2 is characterized by high expression of inflammatory genes, illustrated by expression of *Il1α* in cells with high PC 2 scores (Fig 3A). GO analysis on genes that contribute to PC 2 showed an enrichment in inflammatory functions, confirming the characterization of these genes (Fig 3B). Given the tissue-level differences between conditions (Figure 1), we expected greater inflammatory expression in the WT-infected neutrophils. However, we observed that PC 2 scores, representing inflammatory gene expression, were higher in cells from KO-infected than WT-infected mice (Fig 3C-D, Appendix Fig S3D, Appendix Table S2), Measurement of the differentially expressed genes between conditions further supported this observation (Appendix Fig S3E)."

We added a bar with each gene's coefficient and the corresponding -log(p value) of that gene's expression between conditions to every instance of PC score analysis (Fig 2D, 4G, 5C, S3H, S5G-H) and recorded these values in Appendix Table S2:

D

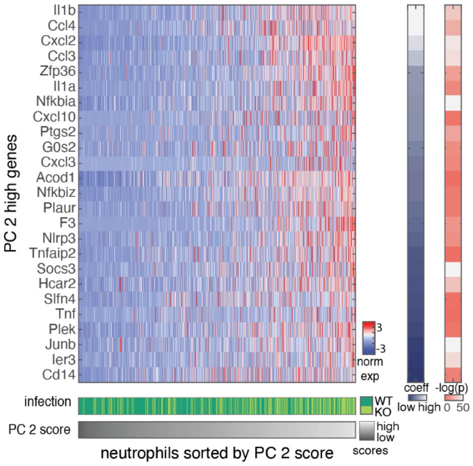

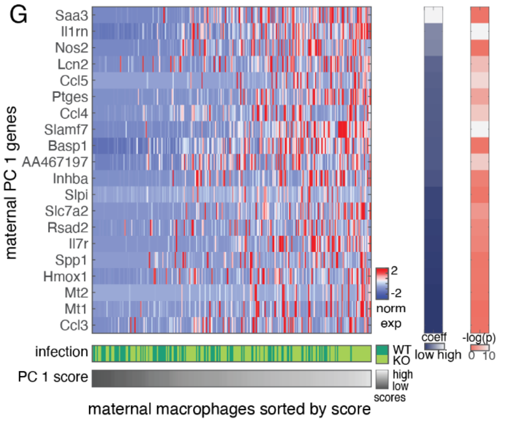

C

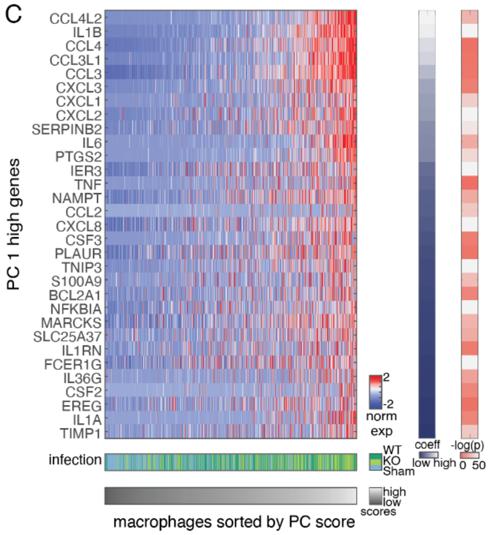

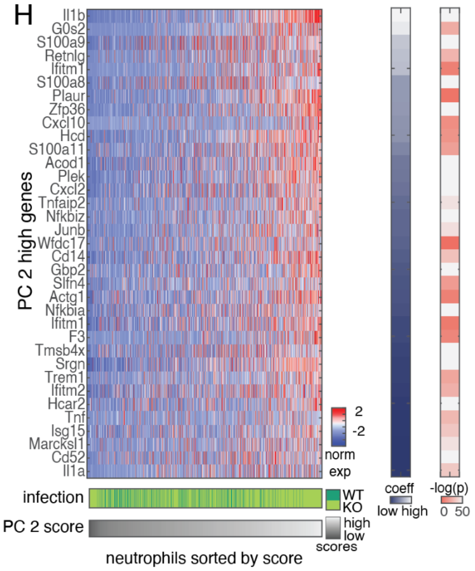

G

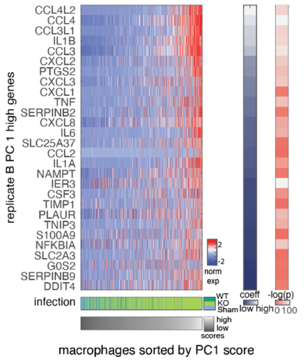

H

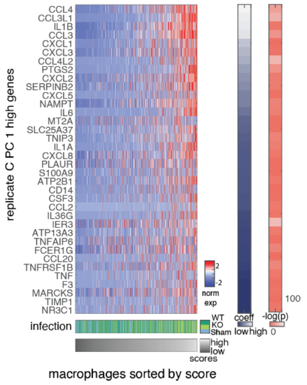

We have confirmed sum expression of PC gene differences with statistical test (Fig S3D, S4D, S5F):

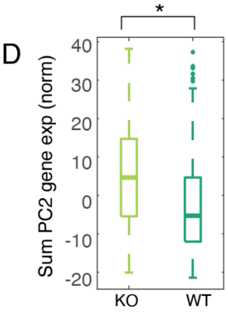

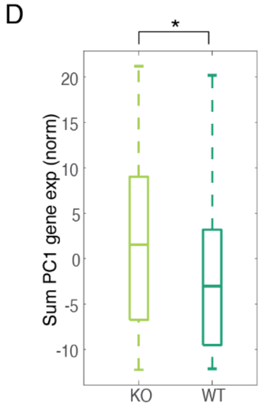

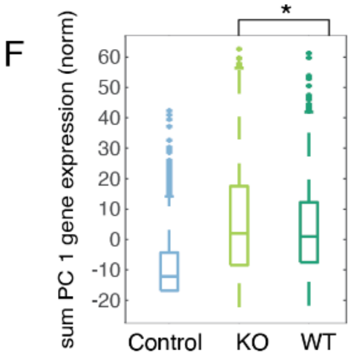

We report differentially expressed genes between conditions for every instance of PC score analysis in the main figures. (In Appendix figure S5 the additional PC score analyses validate the placenta A PC score analysis.) This includes panels S3E and S4E because human maternal macrophage PC score analysis is already accompanied by differential expression analysis in the main figure:

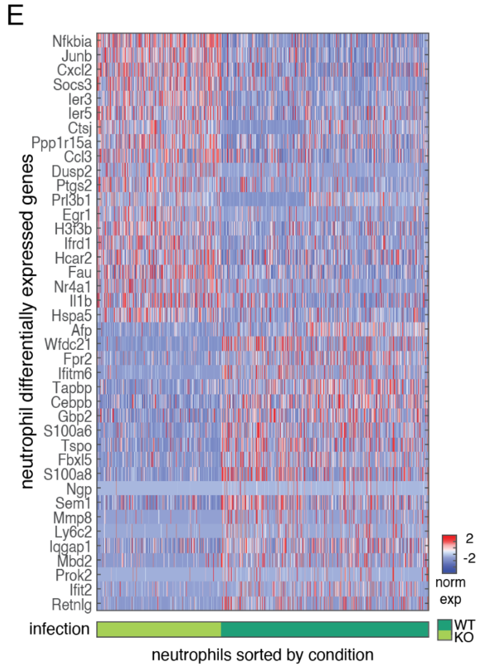

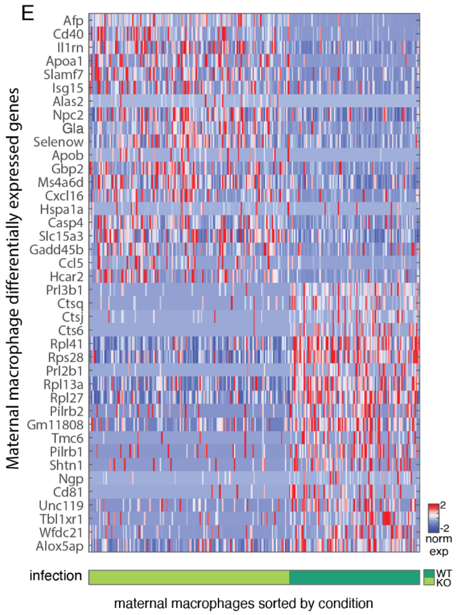

Figure 4E: Because analysis on the limited number of fetal cells was more limited, we instead measured inflammation here just by expression of differentially expressed and upregulated genes in the WT 72h condition, which was a set of genes enriched in inflammatory expression. Therefore, it actually is correct that this is different from the PC score analysis. We have modified the accompanying text to make this clear:

> "Having distinguished this population of macrophages of fetal origin, we asked whether these were affected by GBS infection since they have an important role in mediating

placental functions (Erlebacher, 2013; Mezouar et al., 2021). We queried for differences in fetal macrophage modulation by asking which genes were differentially expressed and upregulated between cells from sham-infected mice or mice infected by β-h/c KO or WT GBS at each time point post infection. We found that fetal macrophages from WT-infected placentas at the 72h time point are the only that exhibit inflammatory activation, based on differentially expressed genes between conditions and comparison of expression of fetal inflammatory genes, derived from differentially expressed genes, across time points (Figure 4d-e, Figure S4b)."

Concern 7: "The authors claimed in-frame cylEΔcat mutant (referred to as β-h/c KO) were used. It is unclear how this mutant is constructed (i.e through allelic exchange?). In bacterial transformation strategy using allelic exchange mutagenesis, the KO phenotype has to be confirmed through complementation analysis where a wild-type copy will need to be re-introduced into β-h/c KO. This to ensure that phenotype observed in Figure 1 (and subsequent assay) is due to cylEΔcat specific gene disruption, and not through polar effects on downstream genes or operons in the β-h/c KO strain. Polar effect is especially common for bacterial ORFs, if not validated."

Response and action: The mutant was constructed from an unmarked in-frame deletion which makes it very unlikely that there are polar effects. Also it is in the middle of the operon that produces Bh/c, and therefore not in the middle of a group of regulatory genes (PMID: 11136446). The mutant has been used and established in other animal models of GBS infection (PMID: 24474814). As the plasmids are not stable *in vivo* we will not be able to do a complementation experiment.

Concern 8: "Figure 1c, 1d: Provide overlapping/colocalization images on GBS, F4/80, and a marker of fetal or placental membranes to justify claim in line 112 page 4 "We observed infection along the fetal and maternal placental membranes with corresponding increase in macrophage staining in the same heavily-colonized tissue regions". How do you exclude that GBS is not colonizing the murine decidua, and specifically the placental membrane?"

Response and Action: We thank the reviewer for clarifying this point. We certainly do not exclude the possibility of decidual colonization, especially given the location of positive GBS staining in WT-infected mice at 48h post infection. In general, we do not exclude the possibility of colonization elsewhere in the tissue, but rather use panels 1c-d to highlight the way that colonization increases over time and the way that macrophages infiltrate the same tissue regions that are increasingly positive for GBS compared to sham (figure s1). We have updated the text to reflect this:

"We also measured GBS and macrophage presence in the tissue by immunofluorescent and histological staining. GBS staining revealed higher bacterial loads in the WT-infected than the KO-infected placentas at each time

point, consistent with the culture results. Specifically we observed infection along the fetal and maternal placental and decidual membranes with a corresponding increase in macrophage staining in the same heavily-colonized tissue regions (Fig 1C-D, Appendix Fig S1A). These data confirm that β-h/c KO GBS induces an attenuated phenotype relative to WT GBS, and that macrophages play a role in mediating the response to GBS. These also suggest that phenotype in both conditions increases at each time point following infection. Having recapitulated these tissue-level phenotypic differences between conditions, we then asked which molecular programs underlie these phenotypic changes and how strain-dependent differences in expression of these programs might lead to the observed tissue-level differences."

Concern 9: "Page 8 line 181: "β-h/c KO GBS-infected neutrophils show higher inflammatory response..." This statement is misleading as the authors did not show that GBS directly infects neutrophils in the mouse placenta. The following paragraph also suggested the term 'infected cells', which needs to be validated if indeed neutrophils is GBS-infected."

Response: This is an important clarification in language, and we are grateful to the reviewer for highlighting it.

Action: We modified the language to clarify this point in the following eight instances in the manuscript including one two pointed out by the reviewer:

> "To determine which cell lineage underwent the greatest transcriptomic changes due to infection, we compared cells within each lineage from sham- and GBS-infected mice. For each lineage, we calculated the Pearson's correlation coefficient between every pair of cells from sham-infected mice. We then calculated the correlation between each pair of cells from sham- and GBS-infected mice and compared the distributions of correlations. We found that the erythrocyte and trophoblast lineages showed similar distributions of pairwise correlations between cells from sham- and GBS-infected mice, but immune lineage cells from GBS-infected mice were less correlated with equivalent cells from sham-infected mice (Fig 2C). The relative composition of cell lineage types present in each condition shows that, as expected, infected mice have an influx of immune lineage cells (Fig 2D, Appendix figure S2G). We note that immune cells from sham-infected mice are not abundant at this time point, but given the large influx of immune cells in the infected mice, it is plausible that these cells are least correlated with placental immune cells present at steady state. On the basis of this shift in the immune cell population in infected mice, we focused our subsequent analysis on characterizing these responses."

> "Figure 2E legend: Histogram of Pearson's correlation coefficients computed on variable genes on cell pairs between cells from sham- vs. GBS-infected mice compared to cells from sham- vs. sham-infected mice within each lineage."

“Neutrophils from β-h/c KO GBS-infected mice show higher inflammatory response than WT GBS-infected at the time of severe phenotype”

“We next asked what the variance captured by PC 2 corresponds to and found that PC 2 is characterized by high expression of inflammatory genes, illustrated by expression of *Il1a* in cells with high PC 2 scores (Fig 3A). GO analysis on genes that contribute to PC 2 showed an enrichment in inflammatory functions, confirming the characterization of these genes (Fig 3B). Given the tissue-level differences between conditions (Figure 1), we expected greater inflammatory expression in the WT-infected neutrophils. However, we observed that PC 2 scores, representing inflammatory gene expression, were higher in cells from KO-infected than WT-infected mice (Fig 3C-D, Appendix Fig S3D, Appendix Table S2), Measurement of the differentially expressed genes between conditions further supported this observation (Appendix Fig S3E).”

“We considered the possibility that the apparent discrepancy observed between transcriptomic and tissue-level data was not representative of the response across the entire dataset. To address this, we compared PC 2 scores between neutrophils from each condition at each time point (Fig 3E, Appendix Fig S3F, see methods). We found that the inflammatory neutrophil dynamics captured by PC 2 scores are not consistent with phenotypic trends over time between conditions: In the early phase post infection (before 48h), neutrophils from WT-infected mice have higher inflammatory expression, indicated by PC 2 scores, than cells from matched KO-infected mice, as we had originally expected (Fig 3E) based on the more severe phenotype in WT- than KO-infected samples at each time point measured by histology and bacterial load (Figure 1). Next, when severe phenotype presents (48h), there is an unexpected shift in relative inflammatory expression between conditions. Neutrophils from KO-infected mice actually have higher inflammatory gene expression than those from WT-infected mice despite having a milder tissue-level phenotype. Finally, this difference is augmented by 72h post infection when the phenotype is the most severe, particularly in the WT-infected mice (Fig 3E, Appendix Fig S3G-H, Fig 1, Appendix Table S2). These results indicate that both strains induce an *Il-1*-family inflammatory response in neutrophils, but at the time of severe phenotype, the response is lower in the cells from WT-infected than β-h/c-KO-infected mice.”

“These results reveal common inflammatory dynamics in maternal macrophages and neutrophils. Cells from WT-infected placentas show greater inflammatory expression relative to those from KO-infected placentas in the first stage post infection, and then at the time when severe phenotype presents (48h post infection), cells from KO-infected mice show greater inflammatory expression than WT-infected. Conversely, fetal macrophages do not show inflammatory expression until the point of severe phenotype, at which point inflammatory expression is induced only in WT-infected fetal macrophages.”

“To test for a difference in response between cells from KO- and WT-infected mice as we observed *in vivo,* we performed PC score analysis on maternal macrophages. The genes that contribute to high PC 1 scores and the GO analysis of those genes revealed an inflammatory program in responding macrophages (Fig 5C-D, Appendix Fig S5F-H, Appendix Table S2).Comparing between conditions, we found that PC 1 scores (a proxy for inflammatory expression) from β-h/c KO-infected macrophages were significantly higher than those from WT-infected macrophages (paired t-test, $P<10^{-13}$, Fig 5E). Analysis in placentas B and C also revealed significantly higher PC 1 scores in KO- than WT-infected maternal macrophages (paired t-test, Rep B: $P<10^{-50}$, Rep C: $P<10^{-19}$, Fig 5E).”

“In this work, we defined the placental cell state dynamics in response to GBS with or without expression of the toxin β-h/c. We first recapitulated the difference in tissue-level phenotype between the response to a β-h/c KO and isogenic WT GBS strain (Randis et al. 2014), using a mouse model of ascending infection. We found that WT GBS induced a more severe phenotype measured by histological analysis and bacterial burden than the β-h/c KO (consistent with prior studies). We analyzed corresponding scRNA-Seq data to understand the cell state changes that accompanied this phenotypic progression. PC score analysis revealed GBS induction of an IL1 family inflammatory response in innate immune cell populations, but different response dynamics depending on the presence of β-h/c. In the first stage after infection (up to 48h), immune cells from WT-infected mice expressed relatively higher inflammatory response genes than those from β-h/c KO-infected mice. In the later stage however, (48-72h), we observed relatively less inflammatory expression in cells from the WT- compared to the β-h/c KO-infected mice, concurrent with the presentation of severe phenotype in WT-infected mice. We found that fetal macrophages were only activated when infected with WT GBS at 72h post infection. These data reveal a largely conserved response between conditions, but our systematic study design and use of scRNA-Seq data as a primary readout reveals subtle shifts in response dynamics that can provide important insight into the tissue-level responses between conditions. While the scRNA-Seq differences are subtle, we validated the relatively lower expression of inflammatory response genes induced by WT- compared to β-h/c KO-GBS in maternal macrophages and neutrophils in an *ex vivo* infection of human placental macrophages, where we found a robust and reproducible difference between conditions.”

Concern 10: “Page10 line 236: 'KO GBS-infected maternal, but not fetal macrophages....' What is the CFU/bacterial burden difference between KO and WT GBS in ex vivo placental macrophage infection assay? Can the author confirm the differences in CFU observed in mice in the infected placental macrophages?”

Response and action: We thank the reviewer for raising this issue. We do not have the data to confirm the difference in bacterial burden in infected cells, but we do not expect a significant difference given that this is a short term *in vitro* assay. The *in vivo* differences we observed are the result of ascension and colonization *in vivo* over the

course of 72h, and this would not be the correct experimental setup to confirm those differences. We have addressed this important concern in our interpretation of these results:

> "Depending on environmental factors, inflammatory activation in the host can either help or hurt the survival of GBS (Landwehr-Kenzel and Henneke 2014). We have shown that over the course of infection, the WT GBS induced relatively more inflammation than β-h/c-KO GBS in the initial response, perhaps aiding its own invasion to and survival in this new niche. Our work introduces a new potential route of evasion for GBS through subversion of the very response that it elicits in the later stage of infection. The relative reduction in inflammatory response which is concurrent with expression of immunoregulatory genes in WT-infected host cells, leads us to propose that β-h/c subversion of the inflammatory response allows increased bacterial proliferation leading to barrier breach and adverse outcomes. An important caveat to consider in interpreting our *ex vivo* validation data is the way bacterial levels in culture impact inflammatory gene expression and protein levels, though we do not expect a significant difference in growth rates in this short-term assay. It will be interesting to consider how factors such as time post infection, bacterial burden or other environmental factors impact inflammatory subversion by GBS."

Concern 11: "It is unclear what is rationale of performing the ex vivo human placental macrophage infection assay for only 3 hours (1 time-point) yet the dynamics of immune cell response of early versus late (12hr vs 72hr) infection in the GBS mouse model Figure 3e and Figure 4e. Along this line, the abstract claims "measure placental cell states changes over time......recapitulated our results in placental macrophages." This is misleading as no time-point experiment was done for the ex vivo human placental macrophage infection assay."

Response: We thank the reviewer for highlighting this point, which is very important to clarify. The reviewer is correct that the *ex vivo* data does not match the time course data collected from infected mice. The time course data allowed us to capture the cell state changes over time in response to different strains. Ultimately the most important finding was not about how the responses change over time. Rather, measuring those changes over time highlights the relative reduction in inflammatory response in immune cells from the WT GBS-infected mice relative to the KO GBS-infected mice. In order to address whether that difference, and the implied suppression of inflammation in the WT GBS-infected samples could result only from the interaction between GBS and placental macrophages, absent any other tissue factors or cell types, we analyzed the response of *ex vivo* placental macrophages to these same strains to specifically to answer whether it was even possible that the KO GBS strain elicited a more robust response than the WT GBS strain in otherwise identical conditions, and it did answer that question. To compare the way the response develops in each condition over time would not really be comparable to the *in vivo*

data, which reflects intravaginal infection and ascension and replication in the placenta. Further, the specific conditions (MOI and duration) that we used for the *ex vivo* infection were the result of optimization experiments that determined what are the appropriate stimulation conditions that will capture the widest range of responses in cells before death. Therefore, these conditions are not meant to replicate *in vivo* infection, but rather to validate the difference in response of these cells to a particular toxin. This is a very important point for us to clarify in the manuscript, and we appreciate that the reviewer has raised it.

Action: We have updated the abstract text so that it reflects exactly the purpose that the *ex vivo* human placental macrophage analysis serves without misleading the reader:

> "Group B *Streptococcus* (GBS) is a pathobiont that can ascend to the placenta and cause adverse pregnancy outcomes, in part through production of the toxin β-hemolysin/cytolysin (β-h/c). Innate immune cells have been implicated in the response to GBS infection, but the impact of β-h/c on their response is poorly defined. We show that GBS modulates innate immune cell states by subversion of host inflammation through β-h/c, allowing worse outcomes. We used an ascending mouse model of GBS infection to measure placental cell state changes over time following infection with a β-h/c-deficient and isogenic wild type GBS strain. Transcriptomic analysis suggests that β-h/c-producing GBS elicit a worse phenotype through suppression of host inflammatory signaling in placental macrophages and neutrophils, and comparison of human placental macrophages infected with the same strains recapitulates these results. Our findings have implications for identification of new targets in GBS disease to support host defense against pathogenic challenge."

Concern 12: "Figure 4g: Cytokine activity in maternal macrophage is significantly modulated between KO and WT GBS stimulation, based on transcriptional profiling. Can the author validate this from the culture supernatant using simple ELISA or Western blot assay?"

Response and action: We thank the reviewer for this suggestion. We have attempted to validate the gene level differences with a few of the significantly differentially expressed genes on culture supernatants, per the suggestion, and we have incorporated the data into Figure 5 and Appendix figure S5 and updated the accompanying text. We found that protein levels of IL6 and MIP1α were also significantly higher at the protein level in the KO- compared to WT-infected cell culture supernatants, and several other cytokines trended higher in the same condition, but that IL1α was more highly upregulated in the WT-infected culture compared to KO-infected, which we suggest may have to do with the rate of translation of IL1α compared to expression of the transcript.

Fig 5g & Appendix Fig. S5i:

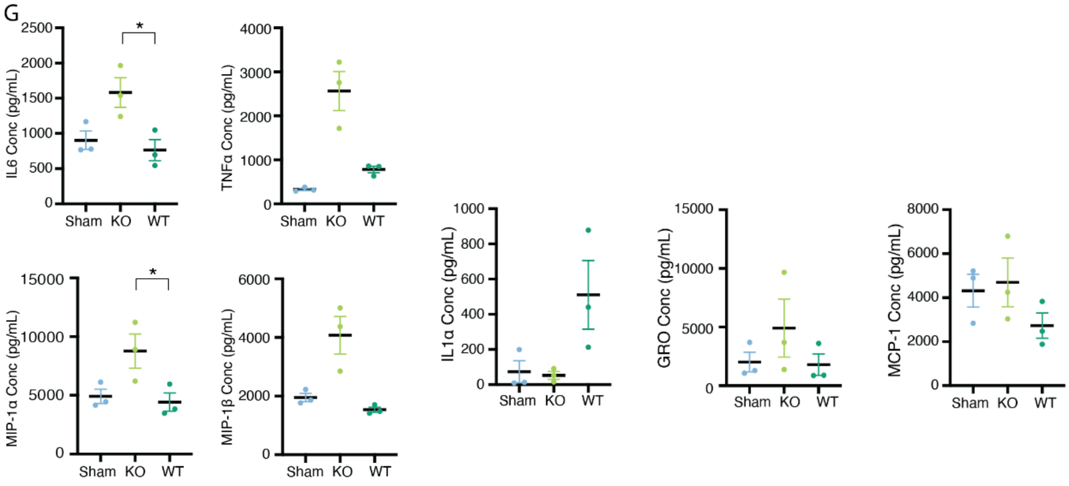

Concern 13: "Page14 line 336: To my knowledge, mouse Hofbauer (HB) macrophages have never been profiled in detail and have never been compared to those from human placentas. It is too generalized to disregard the role of human HBs from Figure 5 which may a role in GBS infection despite not detecting any differences in mouse fetal macrophages. Unless authors perform correlation analysis between mouse fetal macrophages' and human HBs expression profile."

Response and action: We thank the reviewer for raising this important issue. We focus in the paper on validating the effect of maternal immune cells because they orchestrate most of the response in our *in vivo* model, and our observation about HB cell activation is very tissue-context dependent, so our *ex vivo* set up would not provide appropriate comparison for validation. However, we recognize that the differential roles of these two macrophage subsets in response to infection is novel and potentially important, and is actually the subject of future work that we have planned. As such, and because this is not integral to this manuscript, we chose not to discuss it.

Concern 14: "It is very difficult to see the different populations if the authors use many tones of the same color within a plot (e.g., Figures 2b, 2d, S2b). I would suggest the authors to use a more distinguishable color palette for these plots."

Response and action: We thank the reviewer for this comment, and were very happy to update the figures for improved clarity. We kept the color scheme for the main cell lineages similar, but used more distinguishable tones, as you can see below. For the trophoblast lineage subtypes, we changed the colormap, so that they would be more easily distinguishable.

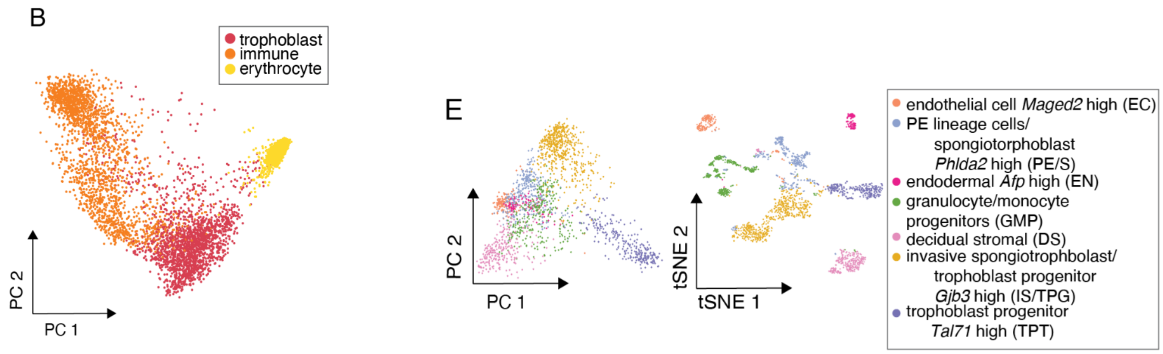

Concern 15: "Scale bars in Figure 1c-d are too small to be understandable. Authors should make them visible to the reader."

Response: We thank the reviewer for pointing this out for the reader's clarity. We increased the size of the text but not the bar itself because that would rescale the image.

Action: We increased the size of the scale bar text in Figure 1 and Appendix figure S1 to improve visibility:

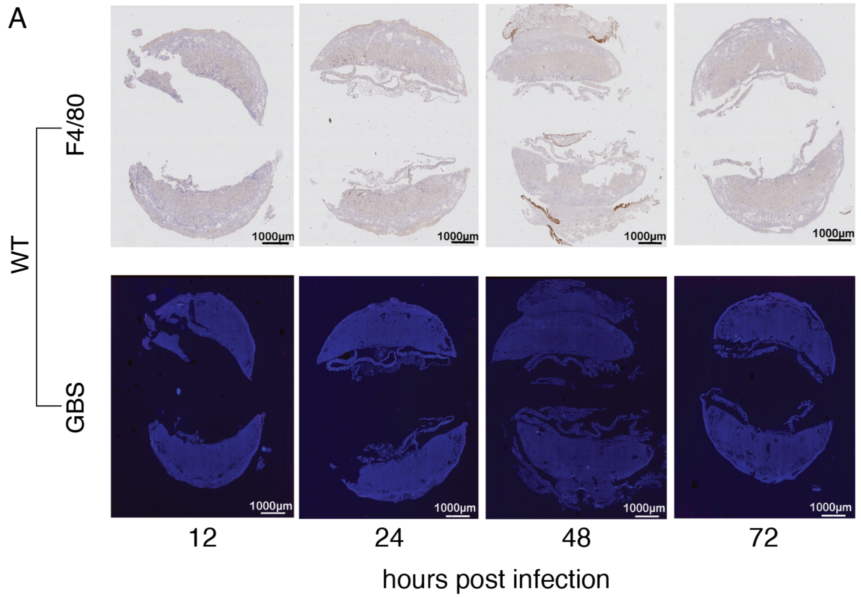

Concern 16: "There are not details about what kit was used for cell hashing, nor which HTO oligos. This is a relevant detail for data analysis."

Response: We thank the reviewer for pointing out these critical missing details. We added information about which specific oligos were used, but we wish to clarify that we did not use a special kit. We used Biolegend hashing antibodies (now specified in the text) with the accompanying protocol provided by Biolegend. We also wish to point

out that as described in the method section entitled “Single cell processing and filtering,” we did not deconvolve the two hashed samples from each mouse for analysis, and therefore, we did not provide further details on hashing.

Action: We have updated the relevant methods section to provide this detail:

Single cell and library preparation for scRNA-Seq:

> “Two placentas from each mouse were washed in PBS and bisected. A small piece was homogenized, serially diluted and plated to confirm GBS colonization of that tissue. The remaining tissue was minced and digested at 37 C for 20’ in DMEM + CaCl2 (0.005%) + Collagenase type IV (20 mg/ml). Red blood cell lysis was performed in ACK Lysing Buffer for 5’. Remaining debris and red blood cells were removed using a lymphocyte separation gradient according to manufacturer instructions. Cells were counted and viability was assessed by trypan blue on a hemocytometer. Two placentas from each mouse were hashed with Biolegend TotalSeqTM– A antibodies using standard protocol. Cells were run on the 10X Genomics Chromium Controller with the Single Cell 3’ v3 system. cDNA amplification and library preparation of the mRNA was processed according to 10X Genomics Single Cell 3′ v3 manufacturer's instructions. For the hashtag oligos, 1ul of HTO PCR additive primer was added to the 10X cDNA amplification step, and the supernatant from the 0.6X cDNA cleanup was kept and processed according to the Cell Hashing protocol, with 14 PCR cycles and a 1.2X cleanup after the PCR.”

Concern 17: “The plots for "Hofbauer cells" and "Y chromosome" in Figure 5b do not match their position written in the figure caption.”

Response: We thank the reviewer for catching this mistake.

Action: We’ve updated the legend for Figure 5b to reflect the correct positions:

> “tSNE of macrophages from placenta A colored by: treatment condition (top left), Y chromosome gene expression (top right), sum expression of marker genes for macrophages of maternal origin (bottom left), Hofbauer cells (bottom right).”

Concern 18: “The authors must specify which genes were used to plot the sum expression for Hofbauer cells and maternal macrophages in Figure 5b, S5a, S5c and S5d. The methodology to do this sum should be explained in the methods section, as it's not clear if the author are just summing the expression of all the genes used as markers.”

Response and action: We thank the reviewer for this comment, which highlights the need for further technical clarification. We have updated the methods section to clarify our identification method and included the list of genes used to identify maternal and Hofbauer subsets.

> "**Identification of ex vivo macrophage subtypes:** Cell type profiles were established based on previously characterized cell types in human placenta (Tetz, Aronoff, and Loch-Caruso 2015; Thomas et al. 2021). Specifically, multiple previously identified maternal macrophage subtypes were combined as were multiple stromal cell types. For one replicate, filtered cells were visualized with tSNE and clustered with hierarchical clustering. Maternal, Hofbauer and stromal clusters were identified by sum expression of combined genes from each of these profiles. To establish robust gene profiles for identification of the three major subtypes across samples, significantly upregulated genes unique to each of the three clusters were identified. Filtered cells from subsequent replicates were visualized with tSNE and clustered with hierarchical clustering. For each, normalized sum expression of uniquely expressed Hofbauer, maternal and stromal marker genes were visualized and cluster identity was assigned. If any cluster showed expression of stromal marker genes, it was eliminated from analysis and another round of tSNE and clustering was performed. This was repeated for each replicate until the only clusters remaining showed expression of HB or maternal macrophage genes."

Appendix Table S4:

Hofbauer genes: *SPP1, TNFRSF4, SGK1, EMP3, EEF1A1, TNFRSF18, ACTG1, PLD3, LPL, PLIN2, JUN, IQGAP2, GPNMB, GADD45B, P2RX7, HMGA1, ETS2, CCDC50, DAB2, CBLB, SQSTM1, MATK, RGCC, OXR1, SLC16A10, NEURL3, TNFRSF12A, FNIP2, IL1RN, SNX8, CD84, HS3ST2, RASGEF1B, EEF1B2, CD9, KLF6, EGR2, DDIT3, CAST, SH3BGRL3, FMNL2, HSP90AB1, ACTB, PDGFA, MYOF, EPS8, RTN4, ACADVL, CD36, MT-ND3, IRF1, IFRD1, ARHGAP10, PLEKHO1, CCND1, GBP2, ANKRD28, HAVCR2, CD109, RCAN1, ANXA5, CD40, NANS, ST3GAL6, ANXA4, CEBPB, PARP14, SRFBP1, SCIN, ABCE1, ITGB5, OGFRL1, DDX21, YBX1, RBMS1, TMEM273,NMB, PPARG, GNG12, RPL10A, LACTB, C6orf48, CXCL16, SLCO4A1, PLA2G16, MT-CYB, ANKRD12, PIM3, WSB1, SEMA6B, EMP1, JAK1, CTSD, TNS3, CD276, PRR5, HPGDS, RPL22L1, CD86, MGAT1*
Maternal genes: *FCER1G, IL1B, TNFAIP6, EREG, SLC7A11, BASP1,SLC25A37, TXN, MGST1, C15orf48, CYBA, SERPINB2, ITGB8, CD48, PILRA, CXCL1, CSTB, AQP9, C1orf122, ADAMDEC1, FTH1, THBS1, MARCKS, CXCL2, CXCL3, IL7R, ANPEP, SERF2, HLA-DRB1, SERPINA1, SRGN, CLEC4E, HLA-DQA1, TSC22D1, MET, CYP1B1, B2M, MYO1G, IL36G, MT-CO1, SLC39A8, PLAC8, MT1E, CXCL5, GM2A, CCL3, RPL36AL, MSC, MT1X, IFI6, PTGS2, MMP14, CSTA, TNIP3, MAP3K20, SEC61G, NCF2, HLA-A, CREG1, ADA, LYZ, S100A9, CAPG, DNAJC3, ATP13A3, HLA-DRA, SERPINB9, CTSH, CARD16, TALDO1, MTRNR2L12, SLC7A7, EHD1, OAZ1, PLD1, MT1F, OST4, BRI3, CXCL8, DYNLL1, PLAUR, S100A8, CYB561A3, IRAK3, MT1M, IL19, TIMP1, IL2RA, LYN, ARPC5, SLAMF7, PTGIR, ATP5F1E, IL10, IDO1, NAMPT, CYTIP, CES1, S100A11, S100A10*

Stromal genes: *CYR61, IL1RL1, SERPINE2, LMCD1, APOD, C7, CXCL14, SPARC, CTGF, IGFBP3, CALD1, RARRES2, EGFL6, GPC3, CRYAB, KRT18, LUM, DCN, DLK1, CDH11, COL1A1, TIMP3, FBLN1, COL6A1, COL6A2, SERPINE1, SERPINF1, COL3A1, SRPX, PTGDS, CNN3, FSTL1, WNT2, SOD3, AKAP12, AEBP1, HGF, DKK1, RSPO3, COL4A2, ADAM12, IGF2, TAGLN, COL4A1, FST, PLOD2, SELENOM, TFPI2, PCOLCE2, SLC22A3, CLMP, COL14A1, MGP, MFAP4, ERRFI1, LARP6, ID3, C1R, PCOLCE, CCDC80, TGFBI, COL1A2, EXT1, FBLN2, FRMD6, ABI3BP, CAVIN1, FHL2, IGFBP5, MSX1, OSMR, PLAC9, TBX3, FGF7, DKK3, MAP1B, VGLL3, UCK2, MYL9, EPAS1, CLU, COL5A1, EFEMP1, FHL1, RRAS2, SPTBN1, IGFBP2, COL15A1, MYC, MEST, MMP23B, MMP2, TEAD1, ENPP1, PRRX1, EGFR, SEMA3B, BDKRB1, DPT, IL13RA2*

Concern 19: "The higher expression of those genes contributing to PC1 in clusters with high PC1 score is expected, Figure 5e. What is the message from this plot?"

Response: We thank the author for pointing this out. Our intention was to characterize the response captured by the first PC and then to measure the difference in expression of the genes that characterize this response by condition. Based on this comment we have revised the figure and accompanying text so that it's better communicated.

Action: We changed the order of panels in the figure so that the genes that characterize the response (5C) precede the GO enrichment of those genes (5D), which, in turn, precede the measurement of those scores across conditions (5E). We have also updated the accompanying text in order to communicate this more clearly.

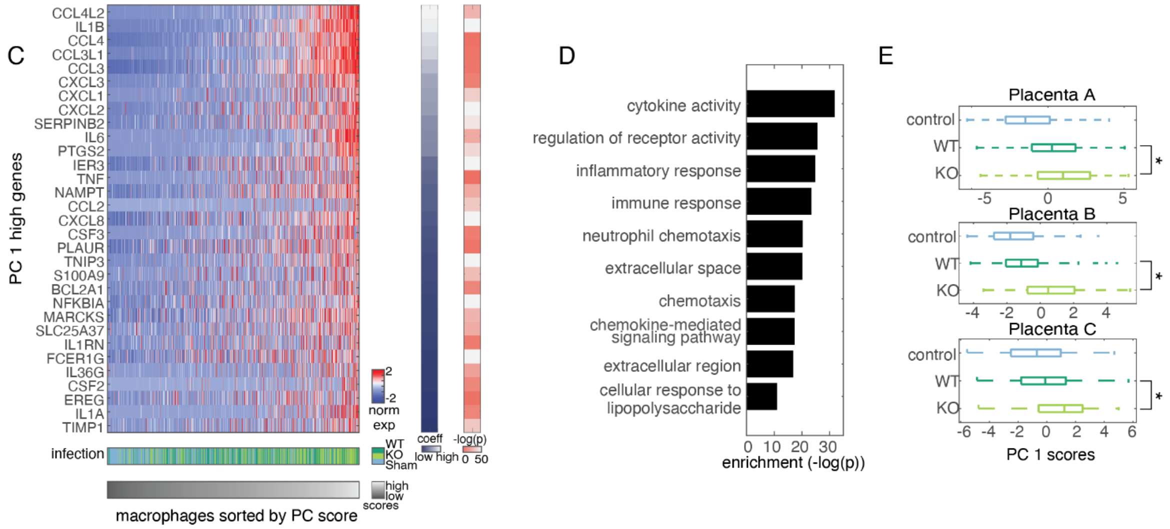

"To test for a difference in response between cells from KO- and WT-infected mice as we observed *in vivo,* we performed PC score analysis on maternal macrophages. The genes that contribute to high PC 1 scores and the GO analysis of those genes revealed an inflammatory program in responding macrophages (Fig 5C-D, Appendix Fig S5F-H, Appendix Table S2).Comparing between conditions, we found that PC 1 scores (a proxy

for inflammatory expression) from β-h/c KO-infected macrophages were significantly higher than those from WT-infected macrophages (paired t-test, $P<10^{-13}$, Fig 5E). Analysis in placentas B and C also revealed significantly higher PC 1 scores in KO- than WT-infected maternal macrophages (paired t-test, Rep B: $P<10^{-50}$, Rep C: $P<10^{-19}$, Fig 5E)."

Concern 20: "The authors should mention/show which are the genes that would support their hypothesis that β-h/c actively suppress inflammation through the induction of suppressive and regulatory genes. Figure 5f."

Response and action: We thank the reviewer for raising this point, which has prompted us to add an additional analysis to figure 5 and expand our discussion of inflammatory regulation in the discussion:

Figure 5H:

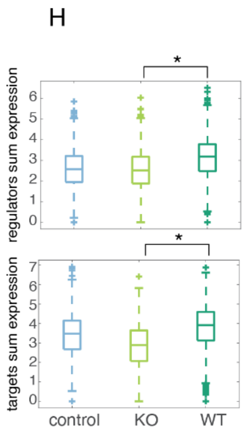

"Our work has highlighted a potential route of GBS disease modulation based on WT GBS-induced gene expression (Figure 5). Some of the upregulated genes in response to WT GBS infection in maternal macrophages have functions in inflammatory regulation, including *IER3, TNFAIP3,* and *BHLHE40 (Arlt and Schäfer, 2011; Cook et al., 2020; Coornaert et al., 2009; Parvatiyar and Harhaj, 2011)*. *BHLHE40* is a transcription factor involved in different cellular processes in a variety of tissues including circadian rhythm regulation and differentiation (Boudjelal et al., 1997; Honma et al., 2002; Shen et al., 2002; Yun et al., 2002). Recent work has implicated this gene in inflammatory regulation in different physiologic contexts (Cook et al., 2020; Jarjour et al., 2019), and we hypothesize that because of its regulatory role in inflammation, targeting *BHLHE40* could limit host inflammatory suppression leading ultimately to better phenotypic outcomes. Consistent with our *in vivo* findings, both the KO-infected and WT-infected conditions show significant inflammatory activation, which is reasonable given that the conserved innate response has evolved to respond to diverse stimuli and the presence or absence of a single toxin should not impact the overall response. The *ex vivo* analysis, however, is designed in a way that can provide some insight into the impact of the presence of β-h/c. It could be the case that these regulatory genes are induced by a stress response

caused by increased greater cell lysis due to β-h/c, in which case regulation would be an indirect consequence of β-h/c, but which allows increased colonization and survival for WT GBS. Future work will define immune regulation better in this setting, and will address precisely how this regulation impacts phenotype in an *in vivo* context. Ultimately, this has important implications for clinical intervention through modulation of this and other inflammatory regulators for improved bacterial control, while limiting the impact on normal host cell function during pregnancy."

Concern 21: "General points on single-cell analysis: Please describe in methods section how on data processing, filtering and integration pipeline given that the general interest for MSB readers are in the field of system biology."

Response and action: We thank the reviewer for raising this point, and we have added information to the methods section in order to improve clarity on data processing. We have added a section on lineage and cell type identification:

"Lineage and cell type identification:

Lineage clusters were generated through hierarchical clustering and identified primarily through expression of marker genes independently from the literature. Data from each time point was clustered independently, and while the three main lineages separated for most time points, at 48h post infection, granulocytes were so inflammatory that they separated out separately for macrophages. In order to maintain consistent cell clustering across time points for comparison, four clusters were isolated from cells at 48h post infection, and the neutrophil and macrophage clusters were combined to form the immune cell lineage. Cluster identity was confirmed based on expression of manually curated genes (Appendix Fig S2B) and differentially expressed genes identified by Ranksum test ($P< 0.0001$) between clusters (Fig 2B). Within the immune and trophoblast lineages, cells were sub clustered and identities were assigned based on expression of manually curated genes from the literature unique to each subcluster (Appendix Figs S2D,F)."

In general, data from each time point was analyzed separately because there were baseline transcriptional changes with each day of gestation. No integration was necessary for samples from different mice within the same time point. We only directly compared across time points for cell state comparisons across time (Figures 3e, 4e, s4e), and we have added details on the way data were integrated for that analysis in the cell state analysis section:

"**Cell state analysis:**

Data from each time point was analyzed separately and major cell lineages, cell types and subtypes were identified separately in the data from each time point. Immune cell subpopulations were isolated for analysis by hierarchical clustering. For each immune

cell population, subpopulations were isolated, as relevant, by additional clustering. PCA was then performed on log transformed data using informative genes which were defined as highly expressed and variable genes (fano-factor and mean expression). For each analysis, genes that contribute to the relevant PC (1 or 2 depending on the analysis) were identified as genes with the highest coefficients for PC 1. Gene Ontology (GO) enrichment confirmed functional characterization of these sets of genes. PC 1 scores were compared between cells of different conditions by paired t-test to determine whether condition explained the major source of variance in each subpopulation of cells. In order to compare PC scores across time points, equivalent cell populations were isolated from the data in each time point, and then scores from the data in each time point were calculated based on mu and coefficients defined in the reference PCA."

Finally, although the filtering thresholds used were the same for *ex vivo* and *in vivo* data, we added an additional section on single cell processing for *ex vivo* data for clarity. Data from different human placentas were not integrated, and we have now specified that.

"Single cell processing and filtering for human placental macrophages:

Cell Ranger pipeline (V.3.0.0) was used for demultiplexing, mapping to the human genome (GRCh38) and counting umis for individual cells. Cells were filtered out if they had less than 500 unique molecular identifiers (UMIs), and if more than 20% or 15% of their transcripts were ribosomal or mitochondrial genes, respectively. We normalized the expression to transcripts per median (TPM) and log transformed the expression. Each placenta was analyzed separately."

Concern 22: "How are the hashtag reads being incorporated into the data?"

Response and Action: Samples were not deconvolved based on hashtags. In order to increase the number of recovered cells from each mouse and in order to improve the representation from each mouse, two adjacent placentas were combined for analysis. We also found that many placental cell types had poor hashing coverage, so deconvolving the samples by hashtags would have significantly limited analysis. We include this information in the methods because it was part of the way samples were treated, but we specify in the relevant analysis section that samples were not deconvolved:

"Single cell processing and filtering:

Cell Ranger pipeline (V.3.0.0) was used for demultiplexing, mapping to the mouse genome (mm10) and counting umis for individual cells. Cells were filtered out if they had less than 500 unique molecular identifiers (UMIs), and if more than 20% or 15% of their transcripts were ribosomal or mitochondrial genes, respectively. We normalized the expression to transcripts per median (TPM) and log transformed the expression. Hashed placentas were not deconvolved for analysis but the two adjacent placentas used for hashing were combined for analysis."

Concern 23: "Is cell cycle a major source of variation in the PCA?"

Response and action: We thank the reviewer for raising this issue, which prompted us to add an analysis to provide this important control. For the two major cell types analyzed with respect to inflammatory response in vivo (neutrophils and macrophages) we have added cell cycle analysis and shown the results in the corresponding supplemental figures. We have also addressed this in the text corresponding to the figures pasted below.For cells infected *ex vivo* for four hours, we do not expect cell division, so we did not perform cell cycle analysis on data included in Figure 5.

Appendix figs S3C and S4B:

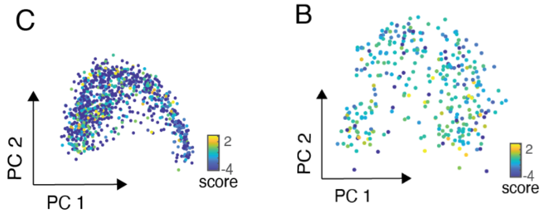

Concern 24: "How are the samples/cell states integrated for analysis?"

Response and action: This is an important point to clarify. Samples were batch processed as much as possible, but libraries from each time point were generated on different days and then sequenced together. It is difficult to distinguish between batch effect and the increasing baseline inflammatory state and other cell state changes in the placenta apparent with each day of pregnancy. It is also difficult in infected samples to distinguish those changes from the response to infection. Therefore, we developed an analytic framework and approach that allows us to compare controlled and subtle changes between conditions and then the differences between conditions (which in some ways are internal controls) and across time points in order to infer meaningful conclusions from these data. One of the advantages of the PC score analysis for these data, is that we were able to compare immune cell state changes in a meaningful way without any batch correction, thus preserving some of the signal, which otherwise would be challenging to distinguish across these different parameters. We have added details addressing the way samples were integrated to the "Cell state analysis" section below. Human placental replicates were all analyzed separately.

> **"Cell state analysis:**

Data from each time point was analyzed separately and major cell lineages, cell types and subtypes were identified separately in the data from each time point. Immune cell subpopulations were isolated for analysis by hierarchical clustering. For each immune cell population, subpopulations were isolated, as relevant, by additional clustering. PCA was then performed on log transformed data using informative genes which were defined as highly expressed and variable genes (fano-factor and mean expression). For each analysis, genes that contribute to the relevant PC (1 or 2 depending on the analysis) were identified as genes with the highest coefficients for PC 1. Gene Ontology (GO) enrichment confirmed functional characterization of these sets of genes. PC 1 scores were compared between cells of different conditions by paired t-test to determine whether condition explained the major source of variance in each subpopulation of cells. In order to compare PC scores across time points, equivalent cell populations were isolated from the data in each time point, and then scores from the data in each time point were calculated based on mu and coefficients defined in the reference PCA."

Concern 25: "Perform downstream differential gene expression analysis (with statistical test) to confirm PCA variation at gene level between KO and WT GBS induced infection"

Response and action: We thank the reviewer for pointing this out and were happy to add this analysis to our manuscript, as discussed in an earlier comment. Differential gene expression analysis was performed each time PC score was used as the primary analysis and differentially upregulated genes were selected by rank sum test between conditions ($P < 0.0001$). We have also added Appendix Table S2 to report the p value of each gene between cells from KO- and WT-infected mice for each relevant analysis. We have also added two bars to each PC score analysis to indicate gene coefficient (and reordered genes to be displayed by coefficient order) as well as the corresponding p value of the paired t-test between cells from KO- and WT-infected mice for each row. When the PC score analysis is in the main figure, we have added overall expression differences by boxplot and p value of the sum of the set of genes in each PC score analysis. Please see our response to concern 6 for the figure panels that address this.

Concern 26: "Figure S5 is not clear what previously identified markers were used for filtering scheme. Please plot all identified markers on the umap."

Response: We thank the reviewer for this comment. We developed a subpopulation identification approach that allowed consistent identification across multiple human donors. Rather than a few individual genes, we developed gene profiles (100 genes each) for identification of these subpopulations, so it would not be possible to show expression of each of them on a tSNE, which we use to visualize these cells. Instead

we have updated the methods to explain our approach better and included lists of these genes.

Action: We have added Appendix Table S2 and explained our approach in more detail in the relevant methods section:

Supplementary table 3:

Hofbauer genes: *SPP1, TNFRSF4, SGK1, EMP3, EEF1A1, TNFRSF18, ACTG1, PLD3, LPL, PLIN2, JUN, IQGAP2, GPNMB, GADD45B, P2RX7, HMGA1, ETS2, CCDC50, DAB2, CBLB, SQSTM1, MATK, RGCC, OXR1, SLC16A10, NEURL3, TNFRSF12A, FNIP2, IL1RN, SNX8, CD84, HS3ST2, RASGEF1B, EEF1B2, CD9, KLF6, EGR2, DDIT3, CAST, SH3BGRL3, FMNL2, HSP90AB1, ACTB, PDGFA, MYOF, EPS8, RTN4, ACADVL, CD36, MT-ND3, IRF1, IFRD1, ARHGAP10, PLEKHO1, CCND1, GBP2, ANKRD28, HAVCR2, CD109, RCAN1, ANXA5, CD40, NANS, ST3GAL6, ANXA4, CEBPB, PARP14, SRFBP1, SCIN, ABCE1, ITGB5, OGFRL1, DDX21, YBX1, RBMS1, TMEM273,NMB, PPARG, GNG12, RPL10A, LACTB, C6orf48, CXCL16, SLCO4A1, PLA2G16, MT-CYB, ANKRD12, PIM3, WSB1, SEMA6B, EMP1, JAK1, CTSD, TNS3, CD276, PRR5, HPGDS, RPL22L1, CD86, MGAT1*
Maternal genes: *FCER1G, IL1B, TNFAIP6, EREG, SLC7A11, BASP1,SLC25A37, TXN, MGST1, C15orf48, CYBA, SERPINB2, ITGB8, CD48, PILRA, CXCL1, CSTB, AQP9, C1orf122, ADAMDEC1, FTH1, THBS1, MARCKS, CXCL2, CXCL3, IL7R, ANPEP, SERF2, HLA-DRB1, SERPINA1, SRGN, CLEC4E, HLA-DQA1, TSC22D1, MET, CYP1B1, B2M, MYO1G, IL36G, MT-CO1, SLC39A8, PLAC8, MT1E, CXCL5, GM2A, CCL3, RPL36AL, MSC, MT1X, IFI6, PTGS2, MMP14, CSTA, TNIP3, MAP3K20, SEC61G, NCF2, HLA-A, CREG1, ADA, LYZ, S100A9, CAPG, DNAJC3, ATP13A3, HLA-DRA, SERPINB9, CTSH, CARD16, TALDO1, MTRNR2L12, SLC7A7, EHD1, OAZ1, PLD1, MT1F, OST4, BRI3, CXCL8, DYNLL1, PLAUR, S100A8, CYB561A3, IRAK3, MT1M, IL19, TIMP1, IL2RA, LYN, ARPC5, SLAMF7, PTGIR, ATP5F1E, IL10, IDO1, NAMPT, CYTIP, CES1, S100A11, S100A10*
Stromal genes: *CYR61, IL1RL1, SERPINE2, LMCD1, APOD, C7, CXCL14, SPARC, CTGF, IGFBP3, CALD1, RARRES2, EGFL6, GPC3, CRYAB, KRT18, LUM, DCN, DLK1, CDH11, COL1A1, TIMP3, FBLN1, COL6A1, COL6A2, SERPINE1, SERPINF1, COL3A1, SRPX, PTGDS, CNN3, FSTL1, WNT2, SOD3, AKAP12, AEBP1, HGF, DKK1, RSPO3, COL4A2, ADAM12, IGF2, TAGLN, COL4A1, FST, PLOD2, SELENOM, TFPI2, PCOLCE2, SLC22A3, CLMP, COL14A1, MGP, MFAP4, ERRFI1, LARP6, ID3, C1R, PCOLCE, CCDC80, TGFBI, COL1A2, EXT1, FBLN2, FRMD6, ABI3BP, CAVIN1, FHL2, IGFBP5, MSX1, OSMR, PLAC9, TBX3, FGF7, DKK3, MAP1B, VGLL3, UCK2, MYL9, EPAS1, CLU, COL5A1, EFEMP1, FHL1, RRAS2, SPTBN1, IGFBP2, COL15A1, MYC, MEST, MMP23B, MMP2, TEAD1, ENPP1, PRRX1, EGFR, SEMA3B, BDKRB1, DPT, IL13RA2*

> **"Identification of *ex vivo* macrophage subtypes:** Cell type profiles were established based on previously characterized cell types in human placenta (Tetz, Aronoff, and Loch-Caruso 2015; Thomas et al. 2021). Specifically, multiple previously identified maternal macrophage subtypes were combined as were multiple stromal cell types. For one replicate, filtered cells were visualized with tSNE and clustered with hierarchical clustering. Maternal, Hofbauer and stromal clusters were identified by sum expression of combined genes from each of these profiles. To establish robust gene profiles for

identification of the three major subtypes across samples, significantly upregulated genes unique to each of the three clusters were identified. Filtered cells from subsequent replicates were visualized with tSNE and clustered with hierarchical clustering. For each, normalized sum expression of uniquely expressed Hofbauer, maternal and stromal marker genes were visualized and cluster identity was assigned. If any cluster showed expression of stromal marker genes, it was eliminated from analysis and another round of tSNE and clustering was performed. This was repeated for each replicate until the only clusters remaining showed expression of HB or maternal macrophage genes."

Concern 27: "Figure 5b What statistical test were used to PC1 scores?"

Response and action: We thank the reviewer for pointing out this missing information. Assuming the reviewer is referring to the PC1 score comparison in Figure 5e, we have updated the accompanying text to specify which statistical test was used:

> "To test for a difference in response between cells from KO- and WT-infected mice as we observed *in vivo,* we performed PC score analysis on maternal macrophages. The genes that contribute to high PC 1 scores and the GO analysis of those genes revealed an inflammatory program in responding macrophages (Fig 5C-D, Appendix Fig S5F-H, Appendix Table S2).Comparing between conditions, we found that PC 1 scores (a proxy for inflammatory expression) from β-h/c KO-infected macrophages were significantly higher than those from WT-infected macrophages (paired t-test, $P<10^{-13}$, Fig 5E). Analysis in placentas B and C also revealed significantly higher PC 1 scores in KO- than WT-infected maternal macrophages (paired t-test, Rep B: $P<10^{-50}$, Rep C: $P<10^{-19}$, Fig 5E)."

Concern 28: "It is not clear if all three human placenta macrophages were integrated for figure5C, figure 5e and figure 5f, whereas figure 5b only show placenta A?"

Response and Action: We thank the reviewer for pointing this out, and we realize this was unclear given the separate analysis of all three replicates in the updated figure 5e. In order to clarify, we have updated the legend to specify exactly which replicate analysis in each panel represents:

> **"Figure 5. Human placental macrophage responses to GBS recapitulate *in vivo* toxin-dependent differences.**
> **A** Schematic of experimental approach. Cells were isolated from human placentas (n=3) and macrophages were selected by CD14 expression. Macrophages were cultured overnight and stimulated with GBS. After 1 hour extracellular GBS was killed and cells were cultured for an additional 3 hours. Following stimulation cells were collected and analyzed by scRNA-Seq.

B tSNE of macrophages from placenta A colored by: treatment condition (top left), Y chromosome gene expression (top right), sum expression of marker genes for macrophages of maternal origin (bottom left), Hofbauer cells (bottom right).

C GO enrichment for the top PC 1 genes in maternal macrophages from placenta A.

D Z-scored expression of PC 1 high genes with corresponding infection condition in placenta A. PC 1 score and condition are indicated in the bars below, and gene coefficient as well as -log(p value) of the expression between KO- and WT-infected cells are indicated in the bars on the right.

E Boxplot of PC 1 scores between conditions on maternal macrophages from each replicate (below, Rep A: $P<10^{-13}$, Rep B: $P<10^{-50}$, Rep C: $P<10^{-19}$).

F Z-scored expression of top differentially expressed and upregulated genes between maternal macrophages from placenta A in each infection condition by rank sum test ($P< 0.0001$).

G Luminex immunoassay of IL6, TNFα, MIP1α and MIP1β levels on supernatants from human placental macrophage cultures. Each point is the average of two technical replicates, each the average of two plate replicates, with exceptions noted in the methods. Bars indicate mean + SEM between biological replicates in each condition. Paired t-test was performed across KO- and WT-infected conditions to determine significance (TNFα, *, $P<0.07$, MIP-1α, *, $P<0.048$).

H Sum expression of upstream regulators (top) and downstream targets (bottom) of *BHLHE40* in maternal macrophages across conditions. Significance was determined by paired t-test between β-h/c KO and WT GBS-infected conditions (regulators: $P<10^{-74}$, targets: $P<10^{-133}$)"

Concern 29: "UMAP from FigureS5 should be properly integrated by donors."

Response: We appreciate this point, but we would like to clarify that analysis on each human placental replicate was performed separately in order to precisely define subpopulations in each dataset. We specify both in the figure and in the legend that panels A, C and D all correspond to different human donors.

Concern 30: "Discussion section: There is no discussion on the limitation on using ascending intravaginal GBS infection model in mouse. First, the vagina microenvironment (pH, microbiome, antimicrobial peptides) and mucosal immunity in mouse is very different from the human. Second, the mouse and human placenta is different in terms of developmental stages, and this will likely have an impact of immune cell properties and responses to infection. It is important to inform the general readers who may not have reproductive immunology background."

Response: We thank the reviewer for highlighting this oversight. While the model we use has been established, validated and used in previous studies, including by team members (Randis et al, 2014), we are very careful about over extrapolating from these data for exactly the reasons that the reviewer highlights. Because we talk about potential

implications of these data in the Discussion, it is essential to discuss these differences for readers that are not as familiar with the field.

Action: We have added the following paragraph to the Discussion:

> "In considering the implications of our findings, it is important to note the limitations of this ascending model of GBS infection in pregnant mice. Mouse and human pregnancy differ in duration, placental structure and development, and nutrient exchange and immune mechanisms at the maternal-fetal interface (Ander et al. 2019; Megli and Coyne 2022; Hemberger et al. 2020). While an advantage of this model is that it has been shown to recapitulate many of the features of clinical disease, it is important to consider these differences in interpreting results, particularly as they relate to inflammatory activation in different immunologic compartments in the placenta. As discussed earlier, the baseline inflammatory activation in the placenta changes with each stage of human pregnancy (Goldstein et al. 2020; Vento-Tormo et al. 2018; Mor and Cardenas 2010). Our in vivo data capture the response to infection in the middle of mouse pregnancy, a time point that corresponds roughly to the second trimester in humans. While we are able to validate our crucial findings in macrophages from human term placentas, suggesting that the changes in response induced by the presence of β-h/c are robust to species, time point in pregnancy and infection duration, it is important to consider that the effect of this interaction on the other cells present and on normal placental function may be different in an infected human, and depending on the point in pregnancy, the inflammatory milieu and the functional demands of the fetus at that stage."

Revision - Editorial Decision **16th Dec 2022**

Thank you for sending us your revised manuscript. We have now heard back from the two reviewers who were asked to evaluate your revised study. As you will see below, reviewer #3 is satisfied with the performed revisions and supports publication pending some minor modifications. Reviewer #2 also list some remaining issues, that seem rather straightforward to address by performing text modifications and providing clarifications. We would ask you to address all these remaining concerns in a final round of minor revision. We would also ask you to address a few editorial issues listed below.

Reviewer #2:

In this manuscript, the authors used single-cell RNA-seq (scRNA-seq) technology to study the transcriptomic profiles of placental cells obtained from a murine model of ascending GBS infection. The authors collected and isolated single cells from placentas infected with a toxin β-hemolysin/cytolysin (β-h/c)-deficient and isogenic wild type GBS strains at different time points post-infection. The authors suggested that β-h/c-producing GBS (wild type) could suppress host inflammatory signaling in placental macrophages and neutrophils to cause a more severe phenotype. The authors also performed ex vivo experimentation using macrophages isolated from human placentas to recapitulate these results.

Specific comments:

1. A possible limitation of this study is the small sample size: n = 4 per sham-injected and per β-h/c-deficient GBS strain-injected mice for 4 time points. Did I understand well? If so, each data set only has one sample.
2. Another possible limitation is how fetal origin was determined. It is understood that fetal origin was determined based on the presence of the Y chromosome from the scRNA-seq results. Since there is only one mouse per time point, it seems very unlikely that there was a male fetus for every time point (as the authors only took two placentas from the same position in each dam). The authors should include the fetal gender in Table S1.
3. Introduction. This section will benefit by adding references showing that there are placental scRNA-seq studies including placentas from term and preterm pregnancies, including those with infection. The cited references are mainly focused in early pregnancy.
4. Introduction. The following claim focused on placental macrophages should be revised: "mediating these pathological outcomes and maintaining a healthy pregnancy is not known" given that there is already evidence showing that maternal and fetal macrophages in the placental tissues can have homeostatic functions in early and late gestation.
5. The authors indicated that the placental single cells were isolated using a lymphocyte separation gradient, which is generally use for mononuclear cells. Neutrophils and trophoblast cells may require a different density gradient. Can you please revise and/or clarify?
6. In Figures 2, 3 and 4, it seems that the main cell types captured were trophoblast, immune cells (neutrophils and macrophages), and erythrocytes. In the mouse placenta, most of the trophoblast cells are syncytiotrophoblasts, which are too large to be captured in the 10x Genomic droplets; yet, there can be CTBs. In addition, it is expected to find lymphocyte populations since the single cells were isolated by lymphocyte preparation, right? Please revise and/or clarify.
7. For the three human placentas in this study, were they all from women with male fetuses? Please clarify.
8. For the methods corresponding to Figure 5, the authors indicated that the decidua basalis was removed from the placental cotyledon. Based on this description, the isolated macrophages should be mainly of fetal origin. Why do the authors think that maternal macrophages were found in the isolated CD14+ cells? Please revise and/or clarify.

Reviewer #3:

The authors have addressed all of the comments in a satisfactory way. The authors have refined the single-cell analysis, added new information and importantly improved the presentation of their results. They have also added more analysis to support their conclusions. In sum, the manuscript advances our understanding on the role of macrophages in the placenta under pathological conditions such as GBS infection.

Minor comments
1) I recommend including the gene name in the figures (or text) to the corresponding proteins measured in the Luminex assay. This will allow an easier interpretation between gene and protein measurements. Figure 5D and S5I.

2) Can the authors explain on figure 5b, referencing the methods "Placental cotyledon tissue was separated from maternal decidua basalis" presumably after digestion, the majority immune cells (CD14+ enriched) should be enriched for Hofbauer cells. Yet, the authors found a large representation of "maternal macrophages" in their human placenta datasets, could these be maternal decidua macrophages?

Response to Reviewes

Reviewer #2:

“In this manuscript, the authors used single-cell RNA-seq (scRNA-seq) technology to study the transcriptomic profiles of placental cells obtained from a murine model of ascending GBS infection. The authors collected and isolated single cells from placentas infected with a toxin β-hemolysin/cytolysin (β-h/c)-deficient and isogenic wild type GBS strains at different time points post-infection. The authors suggested that β-h/c-producing GBS (wild type) could suppress host inflammatory signaling in placental macrophages and neutrophils to cause a more severe phenotype. The authors also performed ex vivo experimentation using macrophages isolated from human placentas to recapitulate these results.”

Concern 1: “A possible limitation of this study is the small sample size: n = 4 per sham-injected and per β-h/c-deficient GBS strain-injected mice for 4 time points. Did I understand well? If so, each data set only has one sample.”

Response and Action: The reviewer is correct that in our initial exploratory dataset there is one mouse per time point per condition for Sham- and KO-infected mice and either one or two from the WT-infected condition depending on the culture results. From each of these mice, two placentas were combined for scRNA-Seq analysis. In designing this cohort for exploratory analysis, we sacrificed additional mice per time point in favor of capturing more conditions and time points. It is for this reason that we validated our gene expression findings on a protein level and we also validated in human placental macrophages. In both of these confirmatory experiments our fundamental findings were robust, so we are confident in these results. Future projects will address different aspects of this dataset which we were not able to validate in the scope of the current paper. In order to eliminate any reader confusion, we have added clarification to Appendix Table S1 and Materials and Methods (changes highlighted in bold):

> **Appendix Table S1: “Treatment and allocation of mouse samples**: 19 timed pregnant mice were treated on Day E13 of pregnancy, 4 in the sham group, 4 in the β-h/c KO group and the remaining mice in the WT GBS group (column 1). Mice were sacrificed at time post infection as indicated in column 2. **From each mouse, two placentas were processed for scRNA-Seq (see Materials and Methods).** CFU/g was determined from the left placenta most proximal to the cervix and CFU/g is indicated in column 3 (Fig 1B). The second most proximal (or third, if second most was used for scRNA-Seq) placenta from each mouse on the left side was fixed for histology and samples that were processed and included in analysis (Fig 1A) are indicated with a “y” in column 4. Vaginal swabs were performed for each mouse as an additional measure of systemic infection. CFU are normalized by volume of PBS and induced in column 5. From each mouse the

two proximal placentas on the right side (or one right and one left if there were not two on the right side) were pooled and processed for scRNA-Seq. From each of those two placentas, a small piece of tissue was cultured for presence of GBS and results are indicated in column 5. If any tissue from a given mouse was culture positive, those samples were included in scRNA-Seq analysis (ie KO2) on the basis that the individual piece of tissue cultured from those placentas may not have shown a culture positive result, but that the mouse hadn't cleared the infection. Because CFU was not normalized by weight, culture results are recorded as positive or negative in column 6. One of the WT-infected mice sacrificed 12h post infection had one culture positive and one culture negative placenta for scRNA-Seq. Because placentas were pooled by hashing, but not deconvolved, the whole sample was included. Culture results may be negative for many technical reasons, but other measures indicate systemic infection. Column 7 indicates whether these samples were included in ("y") or excluded from ("n") scRNA-Seq analysis."

Materials and Methods: "**Single cell processing and filtering for in vivo data.** Cell Ranger pipeline (V.3.0.0) was used for demultiplexing, mapping to the mouse genome (mm10) and counting unique molecular identifiers (UMIs) for individual cells. Cells were filtered out if they had less than 500 UMIs, and if more than 20% or 15% of their transcripts were ribosomal or mitochondrial genes, respectively. We normalized the expression to transcripts per median (TPM) and log transformed the expression. Hashed placentas were not deconvolved for analysis but the two adjacent placentas used for hashing were combined for analysis. **For all cells from sham- and KO-infected mice included in analysis, cells come from two placentas from a single mouse from each time point, and for cells from WT-infected mice, they come from two or four placentas from either one or two mice depending on the colonization results summarized in Appendix Table S1."**

Concern 2: "Another possible limitation is how fetal origin was determined. It is understood that fetal origin was determined based on the presence of the Y chromosome from the scRNA-seq results. Since there is only one mouse per time point, it seems very unlikely that there was a male fetus for every time point (as the authors only took two placentas from the same position in each dam). The authors should include the fetal gender in Table S1."

Response and action: We thank the reviewer for raising this important point. The reviewer is correct that two fetuses are included in the data for each mouse, so it is not a given that each sample will have male fetal cells. Because these two samples are combined, we do not have definitive fetal sex information for each individual placenta, but rather per mouse. However, the way fetal cells were identified involved analysis collectively on all macrophages at each time point, so we only require male fetal cells

present at each time point. The cells were first clustered and then the fetal cluster was identified in part by the presence of more Y chromosome gene-expressing cells from male fetuses. In other words, the Y chromosome cells served as a validation of cluster identity, but was not the sole determining factor of cluster identity. Because this was performed on all macrophages at each time point, it included cells from 6 or 8 placentas, and therefore it makes sense that there were in fact Y chromosome gene-expressing cells present in the analysis at each time point. In order to clarify this point for all future readers, we have modified the text in the results section and Materials and Methods and added a column to Appendix Table S1 indicating whether or not any Y chromosome gene transcripts were detected in the data from each mouse:

> Results: “To compare the dynamics between conditions across time post infection, we measured PC 1 scores **across maternal macrophages defined independently at each time point (see Materials and Methods)**. Consistent with the trend observed in neutrophils, we found that WT-infected macrophages exhibited greater inflammatory expression than β-h/c KO-infected until 48h post infection. By 72h post infection, however, the KO-infected macrophages had higher inflammatory expression than WT-infected (Appendix Fig S4G).”
>
> Materials and Methods: “**Fetal macrophage identification analysis.** Macrophages from male fetuses were identified on the basis of sum log Y chromosome expression above a threshold (0 in mouse and 0.4 in human). Differentially expressed genes were defined between male fetal macrophages and all other cells, and any significant genes were used for hierarchical clustering of all cells into two groups. The group with an overrepresentation of male fetal macrophages was defined as fetal. To measure fetal correlation as a confirmation of identity of non-Y chromosome-expressing macrophages assigned to the fetal cluster, expression of each cell was compared with the average expression of the same set of differentially expressed genes across all confirmed male fetal macrophages. **This analysis was repeated independently on all macrophages included in analysis (from Sham- KO- and WT-infected conditions) at each time point to identify a fetal expression profile unique to that time point. Because analysis at each time point included cells processed from either 6 or 8 placentas, each fetal analysis included some cells from a male fetus to provide a ground truth at that time point.”**
>
> Appendix Table S1: Treatment and allocation of mouse samples: 19 timed pregnant mice were treated on Day E13 of pregnancy, 4 in the sham group, 4 in the β-h/c KO group and the remaining mice in the WT GBS group (column 1). Mice were sacrificed at time post infection as indicated in column 2. From each mouse, two placentas were processed for scRNA-Seq (see Materials and Methods). CFU/g was determined from the left placenta most proximal to the cervix and CFU/g is indicated in column 3 (Fig 1B). The second most proximal (or third, if second most was used for scRNA-Seq) placenta from each mouse on the left side was fixed for histology and samples that were

processed and included in analysis (Fig 1A) are indicated with a “y” in column 4. Vaginal swabs were performed for each mouse as an additional measure of systemic infection. CFU are normalized by volume of PBS and induced in column 5. From each mouse the two proximal placentas on the right side (or one right and one left if there were not two on the right side) were pooled and processed for scRNA-Seq. From each of those two placentas, a small piece of tissue was cultured for presence of GBS and results are indicated in column 5. If any tissue from a given mouse was culture positive, those samples were included in scRNA-Seq analysis (ie KO2) on the basis that the individual piece of tissue cultured from those placentas may not have shown a culture positive result, but that the mouse hadn’t cleared the infection. Because CFU was not normalized by weight, culture results are recorded as positive or negative in column 6. One of the WT-infected mice sacrificed 12h post infection had one culture positive and one culture negative placenta for scRNA-Seq. Because placentas were pooled by hashing, but not deconvolved, the whole sample was included. Culture results may be negative for many technical reasons, but other measures indicate systemic infection. Column 7 indicates whether these samples were included in (“y”) or excluded from (“n”) scRNA-Seq analysis. **Column 8 indicates whether any Y chromosome transcripts were detected in the scRNA-Seq data from that mouse indicating presence of at least one male fetus (“y” = detected and “n” = not detected).**

Concern 3: “Introduction. This section will benefit by adding references showing that there are placental scRNA-seq studies including placentas from term and preterm pregnancies, including those with infection. The cited references are mainly focused in early pregnancy.”

Response and action: We thank the reviewer for highlighting this oversight, and we have added important references to the relevant section in the Introduction (new references highlighted in bold:)

“Macrophages are plastic and heterogeneous cells, in which polarization state and function can differ depending on their environment and ontogeny (Locati, Curtale, and Mantovani 2020). Macrophages in the placenta, which can be maternal or fetal in origin (Yang, Zheng, and Jin 2019; Erlebacher 2013), mediate different functions at different stages of pregnancy including phagocytosis of apoptotic cells, facilitation of invasion and remodeling and cytokine secretion, all of which are important to support a successful pregnancy (Erlebacher 2013; Mezouar et al. 2021; Ander, Diamond, and Coyne 2019; S. Liu et al. 2017; Yao, Xu, and Jin 2019; Reyes and Golos 2018). Throughout pregnancy, placental macrophages maintain a balance of pro- and anti-inflammatory polarization states, and disruption of this balance has been associated with pathological outcomes of pregnancy including miscarriage, preeclampsia and preterm birth (**Yao et al, 2019; Mezouar et al, 2021; Gonzalez et al, 2011; Schonkeren et al, 2011; Guenther et al, 2012; Erlebacher, 2013; Yang et al, 2019; Tsang et al, 2017; Vento-Tormo et al, 2018; Pique-Regi et al, 2019; Miller et al, 2022**).

> Macrophages have also been implicated in the response to GBS infection in the placenta (Randis et al. 2014; Sutton et al. 2019), but their precise role in mediating these pathological outcomes is not fully defined.”

Concern 4: “Introduction. The following claim focused on placental macrophages should be revised: "mediating these pathological outcomes and maintaining a healthy pregnancy is not known" given that there is already evidence showing that maternal and fetal macrophages in the placental tissues can have homeostatic functions in early and late gestation.”

Response and action: We thank the reviewer for this comment and we appreciate the opportunity to clarify our sentence as follows:

> “Macrophages are plastic and heterogeneous cells, in which polarization state and function can differ depending on their environment and ontogeny (Locati, Curtale, and Mantovani 2020). Macrophages in the placenta, which can be maternal or fetal in origin (Yang, Zheng, and Jin 2019; Erlebacher 2013), mediate different functions at different stages of pregnancy including phagocytosis of apoptotic cells, facilitation of invasion and remodeling and cytokine secretion, all of which are important to support a successful pregnancy (Erlebacher 2013; Mezouar et al. 2021; Ander, Diamond, and Coyne 2019; S. Liu et al. 2017; Yao, Xu, and Jin 2019; Reyes and Golos 2018). Throughout pregnancy, placental macrophages maintain a balance of pro- and anti-inflammatory polarization states, and disruption of this balance has been associated with pathological outcomes of pregnancy including miscarriage, preeclampsia and preterm birth (Yao et al, 2019; Mezouar et al, 2021; Gonzalez et al, 2011; Schonkeren et al, 2011; Guenther et al, 2012; Erlebacher, 2013; Yang et al, 2019; Tsang et al, 2017; Vento-Tormo et al, 2018; Pique-Regi et al, 2019; Miller et al, 2022). **Macrophages have also been implicated in the response to GBS infection in the placenta (Randis et al. 2014; Sutton et al. 2019), but their precise role in mediating these pathological outcomes is not fully defined.”**

Concern 5: “The authors indicated that the placental single cells were isolated using a lymphocyte separation gradient, which is generally used for mononuclear cells. Neutrophils and trophoblast cells may require a different density gradient. Can you please revise and/or clarify?”

Response and Action: We thank the reviewer for raising this point, which we would like to clarify. Prior to collecting the data included in this paper we performed numerous pilot studies, where we arrived at this method for single cell isolation based on technical considerations balancing cell yield quality and processing speed. This density gradient is named for its use in lymphocyte separation, but it does not strictly isolate lymphocytes and has been used in our lab for many preparations from whole organs for single cell RNA-Seq. Our intention in this work was not to provide a comprehensive atlas, but rather

to model the changes of cell types we observed that could be recovered by our approach. In our analysis, we therefore inferred cell cluster identity based on comparison of previously annotated cell types with genes expressed in each cluster, and we included genes used to assign all of these cell types in order to be completely transparent in Appendix Figure S2. To ensure clarity, we revised the appropriate text in the Materials and Methods section and addressed this point in the Discussion section where we discuss limitations of the study:

“Discussion: In considering the implications of our findings, it is important to note the limitations of this ascending model of GBS infection in pregnant mice. Mouse and human pregnancy differ in duration, placental structure and development, and nutrient exchange and immune mechanisms at the maternal-fetal interface (Ander, Diamond, and Coyne 2019; Megli and Coyne 2022; Hemberger, Hanna, and Dean 2020). While an advantage of this model is that it has been shown to recapitulate many of the features of clinical disease, it is important to consider these differences in interpreting results, particularly as they relate to inflammatory activation in different immunologic compartments in the placenta. As discussed earlier, the baseline inflammatory activation in the placenta changes with each stage of human pregnancy (Goldstein et al. 2020; Vento-Tormo et al. 2018; Mor and Cardenas 2010). Our in vivo data capture the response to infection in the middle of mouse pregnancy, a time point that corresponds roughly to the second trimester in humans. While we are able to validate our crucial findings in macrophages from human term placentas, suggesting that the changes in response induced by the presence of β-h/c are robust to species, time point in pregnancy and infection duration, it is important to consider that the effect of this interaction on the other cells present and on normal placental function may be different in an infected human, and depending on the point in pregnancy, the inflammatory milieu and the functional demands of the fetus at that stage. **It is also important to consider the way our technical approach may skew the composition of recovered cells and even exclude certain populations, like syncytiotrophoblasts, which are too large to be captured by our single cell method, limiting our ability to interpret these results in the context of the whole tissue environment.** Future work is required to map other cell types and their cell state changes in response to infection in order to compare results meaningfully between different contexts. Finally, further work will also be required to refine our classification of macrophage subtypes in human term placentas. Our cell subtype identification method suggested the presence of macrophages of maternal origin in the placenta even though it had been separated from the maternal decidua (see methods). This suggests the need to refine our current cell type definitions, initially defined by histological features, which may inform our understanding of the behavior of these different cell subtypes in different pathological conditions.”

Materials and methods: “**Single cell and library preparation for scRNA-Seq.** Two placentas from each mouse were washed in PBS and bisected. A small piece was homogenized, serially diluted and plated to confirm GBS colonization of that tissue. The remaining tissue was minced and digested at 37 C for 20’ in DMEM + $CaCl_2$ (0.005%) +

Collagenase type IV (20 mg/ml). Red blood cell lysis was performed in ACK Lysing Buffer for 5’. Remaining debris and red blood cells were removed using a lymphocyte separation gradient according to manufacturer instructions. **We note that the cell isolation method and other technical factors may impact the array of cell types recovered and defined the observed cell types based on genes expressed in those cells.** Cells were counted and viability was assessed by trypan blue on a hemocytometer. Two placentas from each mouse were hashed with Biolegend TotalSeq™– A antibodies using standard protocol. Cells were run on the 10X Genomics Chromium Controller with the Single Cell 3’ v3 system. cDNA amplification and library preparation of the mRNA was processed according to 10X Genomics Single Cell 3′ v3 manufacturer's instructions. For the hashtag oligos, 1ul of HTO PCR additive primer was added to the 10X cDNA amplification step, and the supernatant from the 0.6X cDNA cleanup was kept and processed according to the Cell Hashing protocol, with 14 PCR cycles and a 1.2X cleanup after the PCR.”

Concern 6: “In Figures 2, 3 and 4, it seems that the main cell types captured were trophoblast, immune cells (neutrophils and macrophages), and erythrocytes. In the mouse placenta, most of the trophoblast cells are syncytiotrophoblasts, which are too large to be captured in the 10x Genomic droplets; yet, there can be CTBs. In addition, it is expected to find lymphocyte populations since the single cells were isolated by lymphocyte preparation, right? Please revise and/or clarify.”

Response and Action: We thank the reviewer for raising this important technical concern. We believe that we have addressed this in some part in response to Concern 5, and we would like to reiterate that the reviewer is absolutely correct. There are technical limitations to our cell recovery and after extensive pilot experiments we adapted this protocol as a tradeoff between cell and data quality, yield and consistency across experiments. Analysis of the full range of these populations is out of the scope of this manuscript and future work will be required to address these technical limitations in a way that will also allow analysis of innate immune cells in the context of cell state changes in other placental populations.

To improve clarity, we address this limitation in the Discussion:

> “Discussion: In considering the implications of our findings, it is important to note the limitations of this ascending model of GBS infection in pregnant mice. Mouse and human pregnancy differ in duration, placental structure and development, and nutrient exchange and immune mechanisms at the maternal-fetal interface (Ander, Diamond, and Coyne 2019; Megli and Coyne 2022; Hemberger, Hanna, and Dean 2020). While an advantage of this model is that it has been shown to recapitulate many of the features of clinical disease, it is important to consider these differences in interpreting results, particularly as they relate to inflammatory activation in different immunologic compartments in the placenta. As discussed earlier, the baseline inflammatory activation in the placenta changes with each stage of human pregnancy (Goldstein et al. 2020; Vento-Tormo et al. 2018; Mor

and Cardenas 2010). Our in vivo data capture the response to infection in the middle of mouse pregnancy, a time point that corresponds roughly to the second trimester in humans. While we are able to validate our crucial findings in macrophages from human term placentas, suggesting that the changes in response induced by the presence of β-h/c are robust to species, time point in pregnancy and infection duration, it is important to consider that the effect of this interaction on the other cells present and on normal placental function may be different in an infected human, and depending on the point in pregnancy, the inflammatory milieu and the functional demands of the fetus at that stage. **It is also important to consider the way our technical approach may skew the representation of certain cell populations, such as the syncytiotrophoblasts, which are too large to be captured by our single cell method, thus limiting our ability to interpret these results in the context of the whole tissue environment. Future work is required to map other cell types and their cell state changes in response to infection in order to compare results meaningfully between different contexts. Finally, further work will also be required to refine our classification of macrophage subtypes in human term placentas.** Our cell subtype identification method suggested the presence of macrophages of maternal origin in the placenta even though it had been separated from the maternal decidua (see methods). This suggests the need to refine our current cell type definitions, initially defined by histological features, which may inform our understanding of the behavior of these different cell subtypes in different pathological conditions."

Concern 7: "For the three human placentas in this study, were they all from women with male fetuses? Please clarify."

Response and action: We thank the reviewer for raising this point and took the opportunity to ensure clarity for future readers. Sex of the human fetuses was already reported in the text:

"Cells were isolated from 3 individual full-term human placentas from GBS-negative women: 1 with a male fetus (Replicate A) and 2 with female fetuses (Replicates B and C, see methods)."

We highlighted this in subsequent Results text as well to enforce the point:

"In each sample, visualization of macrophages with tSNE revealed two clusters, corresponding to placental macrophages of maternal and fetal origin (Hofbauer cells), based on expression of previously annotated marker genes (Thomas et al. 2021)(Fig 5B, Appendix Fig S5D). As with mouse placental macrophages, we measured expression of Y chromosome genes in human placental macrophages from Replicate A, which had a male fetus. We found expression of Y chromosome genes in the cells that express Hofbauer marker genes, validating the fetal origin

of these cells (Fig 5B). **This gene set was used to identify macrophages of fetal origin in replicates B and C, which had female fetuses.** We removed from analysis a number of cells (209 out of 5520) that clustered with maternal macrophages and were positive for Y chromosome gene expression, but their presence did not affect the results of the analysis (Appendix Fig S5E). Because most of the responding macrophages captured in our in vivo data were maternal macrophages, we focused our analysis on the response of the equivalent human placental population."

Concern 8: "For the methods corresponding to Figure 5, the authors indicated that the decidua basalis was removed from the placental cotyledon. Based on this description, the isolated macrophages should be mainly of fetal origin. Why do the authors think that maternal macrophages were found in the isolated CD14+ cells? Please revise and/or clarify."

Response and action: We thank the reviewer for raising this point. In our preliminary analysis and pilot studies we considered this question a great deal. First of all these could be placental associated maternal macrophages which are typically found within what's considered to be the "fetal" part of the tissue. For the purpose of this study we define all non-Hofbauer cells (whose identity was validated by Y chromosome gene expression in one of our samples) as "maternal macrophages." It is also important to note that the original classifications of macrophage subpopulations were based, in part, on physical location, but single cell RNA-Seq now allows us to revisit these definitions. As we described with regard to the cell populations recovered in our *in vivo* data, we relied on gene expression between clusters to define cell identity and we included the set of genes we used to define clusters in Appendix Table S3. Because Placenta A had a male fetus, we were able to validate the fetal origin of inferred Hofbauer cells, although the proportion of Hofbauer relative to material isolated from the placenta was smaller than expected. We have addressed this in the Discussion section and we acknowledge that this is a very important question for future work outside of the scope of this manuscript. We also modified the materials and methods to clarify that placental cotyledon was separated prior to digestion in order to avoid contamination from decidual cells.

"In considering the implications of our findings, it is important to note the limitations of this ascending model of GBS infection in pregnant mice. Mouse and human pregnancy differ in duration, placental structure and development, and nutrient exchange and immune mechanisms at the maternal-fetal interface (Ander, Diamond, and Coyne 2019; Megli and Coyne 2022; Hemberger, Hanna, and Dean 2020). While an advantage of this model is that it has been shown to recapitulate many of the features of clinical disease, it is important to consider these differences in interpreting results, particularly as they relate to inflammatory activation in different immunologic compartments in the placenta. As discussed earlier, the baseline inflammatory activation in the placenta changes with each stage of human pregnancy (Goldstein et al. 2020; Vento-Tormo et al. 2018; Mor

and Cardenas 2010). Our in vivo data capture the response to infection in the middle of mouse pregnancy, a time point that corresponds roughly to the second trimester in humans. While we are able to validate our crucial findings in macrophages from human term placentas, suggesting that the changes in response induced by the presence of β-h/c are robust to species, time point in pregnancy and infection duration, it is important to consider that the effect of this interaction on the other cells present and on normal placental function may be different in an infected human, and depending on the point in pregnancy, the inflammatory milieu and the functional demands of the fetus at that stage. It is also important to consider the way our technical approach may skew the composition of recovered cells and even exclude certain populations, like syncytiotrophoblasts, which are too large to be captured by our single cell method, limiting our ability to interpret these results in the context of the whole tissue environment. Future work is required to map other cell types and their cell state changes in response to infection in order to compare results meaningfully between different contexts. **Finally, further work will also be required to refine our classification of macrophage subtypes in human term placentas. Our cell subtype identification method suggested the presence of macrophages of maternal origin in the placenta even though it had been separated from the maternal decidua (see methods). This suggests the need to refine our current cell type definitions, initially defined by histological features, which may inform our understanding of the behavior of these different cell subtypes in different pathological conditions.”**

“Placental macrophage isolation. The villous core of human placentas were isolated as previously described (Tetz, Aronoff, and Loch-Caruso 2015). Placental cotyledon tissue was separated from maternal decidua basalis **prior** to digestion and washed in PBS to remove blood and any clots. The tissue is minced well and placed into 250 mL sterile bottles with sterile digestion solution containing 150 mg/mL deoxyribonuclease, 1mg/mL collagenase, and 1mg/mL hyaluronidase at 10 mL per gram of tissue. Placenta was enzymatically digested at 37oC shaking at 180 RPM for 1 hour. Cells were filtered from undigested tissue using a 280 mm metal sieve followed by 180 and 80 mm nylon screens. After centrifugation at 1500 RPM, cells were resuspended in a cold 25% Percoll solution (Percoll: RPMI) and overlaid onto 50% Percoll with 2mL of 1X PBS on the top of the density gradient. This was followed by a 40 minute spin without a break. After subsequent red blood cell lysis, CD14+ macrophages were positively selected using the magnetic MAC® large cell separation column system according to the manufacturer’s instructions. These cells were plated in a 12 well dish at 2x106 cells/well and rested overnight in RPMI with 10% FBS and 1% antibiotic-antimycotic media at 37oC with 5% CO2 before experimentation.”

<u>Reviewer #3:</u>

"The authors have addressed all of the comments in a satisfactory way. The authors have refined the single-cell analysis, added new information and importantly improved the presentation of their results. They have also added more analysis to support their conclusions. In sum, the manuscript advances our understanding on the role of macrophages in the placenta under pathological conditions such as GBS infection."

Concern 1: "I recommend including the gene name in the figures (or text) to the corresponding proteins measured in the Luminex assay. This will allow an easier interpretation between gene and protein measurements. Figure 5D and S5I."

Response and action: We thank the reviewer for this helpful suggestion. We have modified the figures and corresponding legends. We have changed the names in the text and included both names in the methods (see below):

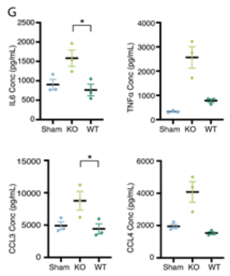

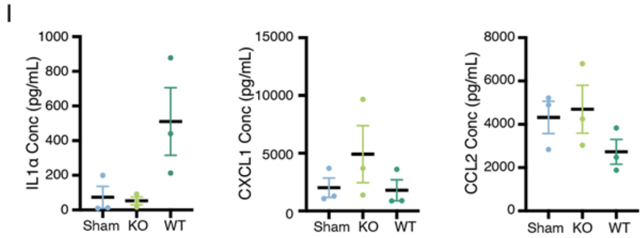

Text: "The relative reduction in inflammatory response in the WT relative to the KO condition led to our hypothesis that the presence of β-h/c suppresses this response in the maternal intrauterine compartment. To investigate this possibility, we queried for genes that are differentially expressed and upregulated in response to each infection condition. Consistent with the results of PC score analysis, KO-infected macrophages were significantly enriched in IL-1-inflammatory response genes (Fig 5F). We validated higher expression of a subset of these genes with protein-level comparison between conditions (see methods). We found that IL6 and CCL3 were significantly higher in KO- than WT-infected cells ($P<0.02$ and $P<0.048$ respectively), and TNFα, CCL4, CXCL1 and CCL2 trended higher in KO- than WT-infected cells. Il1α levels were elevated in WT- relative to KO-infected (NS), which may reflect post translational processing of IL1α (Rubartelli et al. 1993) (Fig 5G, Appendix Fig S5I)."

Methods: "**Cytokines and chemokines measurement.** Cell culture supernatants were screened to measure the levels of multiple analytes (TNF-α, IL-6, IL-1α, GRO (CXCL1), MCP-1 (CCL2), MIP-1α (CCL3) and MIP-1β (CCL4)) using a customized version of the MILLIPLEX MAP Human Cytokine/Chemokine Magnetic

Bead Panel (#HCYTOMAG-60k, MilliporeSigma). Samples were thawed on ice, centrifuged at 10,000 x g for 10 minutes to remove particulates, and then added to premixed beads, according to the manufacturer's protocol. A Luminex® 200TM instrument was used to collect and measure the Median Fluorescent Intensity (MFI) of 50 beads per analyte per well, following kit's instructions. The assay contained recombinant protein standards (Percentage of recovery within 75%-120% range) and quality control (QC) samples (high and low concentration) for each analyte. A 4- or 5-parameter logistic function with 1/y2 weighting was fit using Belysa® v.1 analysis software (MilliporeSigma) to calculate each cytokine's concentration. The lower limit of quantitation was defined by the software for each analyte. Samples with % CV that >25% were excluded from analysis."

Concern 2: "Can the authors explain on figure 5b, referencing the methods "Placental cotyledon tissue was separated from maternal decidua basalis" presumably after digestion, the majority immune cells (CD14+ enriched) should be enriched for Hofbauer cells. Yet, the authors found a large representation of "maternal macrophages" in their human placenta datasets, could these be maternal decidua macrophages?"

Response and action: We thank the reviewer for raising this point, which was also made by Reviewer 2. As we wrote also to them above: In our preliminary analysis and pilot studies we considered this question a great deal. First of all these could be placental associated maternal macrophages which are typically found within what's considered to be the "fetal" part of the tissue. For the purpose of this study we define all non-Hofbauer cells (whose identity was validated by Y chromosome gene expression in one of our samples) as "maternal macrophages." It is also important to note that the original classifications of macrophage subpopulations were based, in part, on physical location, but single cell RNA-Seq now allows us to revisit these definitions. As we described with the cell populations recovered in our *in vivo* data, we relied on gene expression between clusters to define cell identity and we included the set of genes we used to define clusters in Appendix Table S3. Because Placenta A had a male fetus, we were able to validate the fetal origin of inferred Hofbauer cells, although the proportion of Hofbauer relative to material isolated from the placenta was smaller than expected. We have addressed this in the Discussion section and we acknowledge that this is a very important question for future work outside of the scope of this manuscript. We also modified the materials and methods to clarify that placental cotyledon was separated prior to digestion in order to avoid contamination from decidual cells.

"In considering the implications of our findings, it is important to note the limitations of this ascending model of GBS infection in pregnant mice. Mouse and human pregnancy differ in duration, placental structure and development, and nutrient exchange and immune mechanisms at the maternal-fetal interface (Ander, Diamond, and Coyne 2019; Megli and Coyne 2022; Hemberger, Hanna, and Dean 2020). While an advantage of this model is that it has been shown to recapitulate many of the features of clinical disease, it is important to consider these

differences in interpreting results, particularly as they relate to inflammatory activation in different immunologic compartments in the placenta. As discussed earlier, the baseline inflammatory activation in the placenta changes with each stage of human pregnancy (Goldstein et al. 2020; Vento-Tormo et al. 2018; Mor and Cardenas 2010). Our in vivo data capture the response to infection in the middle of mouse pregnancy, a time point that corresponds roughly to the second trimester in humans. While we are able to validate our crucial findings in macrophages from human term placentas, suggesting that the changes in response induced by the presence of β-h/c are robust to species, time point in pregnancy and infection duration, it is important to consider that the effect of this interaction on the other cells present and on normal placental function may be different in an infected human, and depending on the point in pregnancy, the inflammatory milieu and the functional demands of the fetus at that stage. It is also important to consider the way our technical approach may skew the composition of recovered cells and even exclude certain populations, like syncytiotrophoblasts, which are too large to be captured by our single cell method, limiting our ability to interpret these results in the context of the whole tissue environment. Future work is required to map other cell types and their cell state changes in response to infection in order to compare results meaningfully between different contexts. **Finally, further work will also be required to refine our classification of macrophage subtypes in human term placentas. Our cell subtype identification method suggested the presence of macrophages of maternal origin in the placenta even though it had been separated from the maternal decidua (see methods). This suggests the need to refine our current cell type definitions, initially defined by histological features, which may inform our understanding of the behavior of these different cell subtypes in different pathological conditions.”**

“Placental macrophage isolation. The villous core of human placentas were isolated as previously described (Tetz, Aronoff, and Loch-Caruso 2015). Placental cotyledon tissue was separated from maternal decidua basalis **prior** to digestion and washed in PBS to remove blood and any clots. The tissue is minced well and placed into 250 mL sterile bottles with sterile digestion solution containing 150 mg/mL deoxyribonuclease, 1mg/mL collagenase, and 1mg/mL hyaluronidase at 10 mL per gram of tissue. Placenta was enzymatically digested at 37oC shaking at 180 RPM for 1 hour. Cells were filtered from undigested tissue using a 280 mm metal sieve followed by 180 and 80 mm nylon screens. After centrifugation at 1500 RPM, cells were resuspended in a cold 25% Percoll solution (Percoll: RPMI) and overlaid onto 50% Percoll with 2mL of 1X PBS on the top of the density gradient. This was followed by a 40 minute spin without a break. After subsequent red blood cell lysis, CD14+ macrophages were positively selected using the magnetic MAC® large cell separation column system according to the manufacturer’s instructions. These cells were plated in a 12 well dish at 2x106 cells/well and rested overnight in RPMI with 10% FBS and 1% antibiotic-antimycotic media at 37oC with 5% CO2 before experimentation.”

2nd Revision - Editorial Decision

10th Jan 2023

Thank you again for sending us your revised manuscript and for making the last requested edits. We are now satisfied with the modifications made and I am pleased to inform you that your paper has been accepted for publication.

EMBO Press Author Checklist

Corresponding Author Name: Itai Yanai
Journal Submitted to: Molecular Systems Biology
Manuscript Number: MSB-2022-11021

USEFUL LINKS FOR COMPLETING THIS FORM
The EMBO Journal - Author Guidelines
EMBO Reports - Author Guidelines
Molecular Systems Biology - Author Guidelines
EMBO Molecular Medicine - Author Guidelines

Reporting Checklist for Life Science Articles (updated January
This checklist is adapted from Materials Design Analysis Reporting (MDAR) Checklist for Authors. MDAR establishes a minimum set of requirements in transparent reporting in the life sciences (see Statement of Task: 10.31222/osf.io/9sm4x). Please follow the journal's guidelines in preparing your
Please note that a copy of this checklist will be published alongside your article.

Abridged guidelines for figures
1. Data
The data shown in figures should satisfy the following conditions:
- the data were obtained and processed according to the field's best practice and are presented to reflect the results of the experiments in an accurate and unbiased manner.
- ideally, figure panels should include only measurements that are directly comparable to each other and obtained with the same assay.
- plots include clearly labeled error bars for independent experiments and sample sizes. Unless justified, error bars should not be shown for technical
- if n<5, the individual data points from each experiment should be plotted. Any statistical test employed should be justified.
- Source Data should be included to report the data underlying figures according to the guidelines set out in the authorship guidelines on Data

2. Captions
Each figure caption should contain the following information, for each panel where they are relevant:
- a specification of the experimental system investigated (eg cell line, species name).
- the assay(s) and method(s) used to carry out the reported observations and measurements.
- an explicit mention of the biological and chemical entity(ies) that are being measured.
- an explicit mention of the biological and chemical entity(ies) that are altered/varied/perturbed in a controlled manner.
- the exact sample size (n) for each experimental group/condition, given as a number, not a range;
- a description of the sample collection allowing the reader to understand whether the samples represent technical or biological replicates (including how many animals, litters, cultures, etc.).
- a statement of how many times the experiment shown was independently replicated in the laboratory.
- definitions of statistical methods and measures:
  - common tests, such as t-test (please specify whether paired vs. unpaired), simple χ2 tests, Wilcoxon and Mann-Whitney tests, can be unambiguously identified by name only, but more complex techniques should be described in the methods section;
  - are tests one-sided or two-sided?
  - are there adjustments for multiple comparisons?
  - exact statistical test results, e.g., P values = x but not P values < x;
  - definition of 'center values' as median or average;
  - definition of error bars as s.d. or s.e.m.

Please complete ALL of the questions below.
Select "Not Applicable" only when the requested information is not relevant for your study.

Materials

Newly Created Materials	Information included in the manuscript?	In which section is the information available? (Reagents and Tools Table, Materials and Methods, Figures, Data Availability Section)
New materials and reagents need to be available; do any restrictions apply?	Not Applicable	

Antibodies	Information included in the manuscript?	In which section is the information available? (Reagents and Tools Table, Materials and Methods, Figures, Data Availability Section)
For **antibodies** provide the following information:<br>- Commercial antibodies: RRID (if possible) or supplier name, catalogue number and or/clone number<br>- Non-commercial: RRID or citation	Not Applicable	

DNA and RNA sequences	Information included in the manuscript?	In which section is the information available? (Reagents and Tools Table, Materials and Methods, Figures, Data Availability Section)
**Short novel DNA or RNA including primers, probes**: provide the sequences.	Not Applicable	

Cell materials	Information included in the manuscript?	In which section is the information available? (Reagents and Tools Table, Materials and Methods, Figures, Data Availability Section)
**Cell lines:** Provide species information, strain. Provide accession number in repository **OR** supplier name, catalog number, clone number, and/**OR** RRID.	Not Applicable	
**Primary cultures:** Provide species, strain, sex of origin, genetic modification status.	Yes	Materials and Methods
Report if the cell lines were recently **authenticated** (e.g., by STR profiling) and tested for mycoplasma contamination.	Not Applicable	

Experimental animals	Information included in the manuscript?	In which section is the information available? (Reagents and Tools Table, Materials and Methods, Figures, Data Availability Section)
**Laboratory animals or Model organisms:** Provide species, strain, sex, age, genetic modification status. Provide accession number in repository **OR** supplier name, catalog number, clone number, **OR** RRID.	Yes	Materials and Methods
**Animal observed in or captured from the field:** Provide species, sex, and age where possible.	Not Applicable	
Please detail **housing and husbandry conditions**.	Yes	Materials and Methods

Plants and microbes	Information included in the manuscript?	In which section is the information available? (Reagents and Tools Table, Materials and Methods, Figures, Data Availability Section)
**Plants:** provide species and strain, ecotype and cultivar where relevant, unique accession number if available, and source (including location for collected wild specimens).	Not Applicable	
**Microbes:** provide species and strain, unique accession number if available, and source.	Yes	Materials and Methods

Human research participants	Information included in the manuscript?	In which section is the information available? (Reagents and Tools Table, Materials and Methods, Figures, Data Availability Section)
If collected and within the bounds of privacy constraints report on age, sex and gender or ethnicity for all study participants.	Not Applicable	

Core facilities	Information included in the manuscript?	In which section is the information available? (Reagents and Tools Table, Materials and Methods, Figures, Data Availability Section)
If your work benefited from core facilities, was their service mentioned in the acknowledgments section?	Yes	Acknowledgements

Design

Study protocol	Information included in the manuscript?	In which section is the information available? (Reagents and Tools Table, Materials and Methods, Figures, Data Availability Section)
If study protocol has been **pre-registered, provide DOI in the manuscript**. For clinical trials, provide the trial registration number **OR** cite DOI.	Not Applicable	
Report the **clinical trial registration number** (at ClinicalTrials.gov or equivalent), where applicable.	Not Applicable	

Laboratory protocol	Information included in the manuscript?	In which section is the information available? (Reagents and Tools Table, Materials and Methods, Figures, Data Availability Section)
Provide DOI OR other citation details if **external detailed step-by-step protocols** are available.	Not Applicable	

Experimental study design and statistics	Information included in the manuscript?	In which section is the information available? (Reagents and Tools Table, Materials and Methods, Figures, Data Availability Section)
Include a statement about **sample size** estimate even if no statistical methods were used.	Not Applicable	
Were any steps taken to minimize the effects of subjective bias when allocating animals/samples to treatment (e.g. **randomization procedure**)? If yes, have they been described?	Not Applicable	
Include a statement about **blinding** even if no blinding was done.	Not Applicable	
Describe **inclusion/exclusion criteria** if samples or animals were excluded from the analysis. Were the criteria pre-established?<br><br>If sample or data points were omitted from analysis, report if this was due to attrition or intentional exclusion and provide justification.	Yes	Materials and Methods
For every figure, are **statistical tests** justified as appropriate? Do the data meet the assumptions of the tests (e.g., normal distribution)? Describe any methods used to assess it. Is there an estimate of variation within each group of data? Is the variance similar between the groups that are being statistically compared?	Yes	Figures, Materials and Methods

Sample definition and in-laboratory replication	Information included in the manuscript?	In which section is the information available? (Reagents and Tools Table, Materials and Methods, Figures, Data Availability Section)
In the figure legends: state number of times the experiment was **replicated** in laboratory.	Yes	Figures, Materials and Methods
In the figure legends: define whether data describe **technical or biological replicates**.	Not Applicable	

Ethics

Ethics	Information included in the manuscript?	In which section is the information available? (Reagents and Tools Table, Materials and Methods, Figures, Data Availability Section)
Studies involving **human participants**: State details of **authority granting ethics approval** (IRB or equivalent committee(s), provide reference number for approval.	Not Applicable	
Studies involving **human participants**: Include a statement confirming that **informed consent** was obtained from all subjects and that the experiments conformed to the principles set out in the WMA Declaration of Helsinki and the Department of Health and Human Services Belmont Report.	Not Applicable	
Studies involving **human participants:** For publication of **patient photos**, include a statement confirming that consent to publish was obtained.	Not Applicable	
Studies involving experimental **animals**: State details of **authority granting ethics approva**l (IRB or equivalent committee(s), provide reference number for approval. Include a statement of compliance with ethical regulations.	Yes	Materials and Methods
Studies involving **specimen and field samples:** State if relevant **permits** obtained, provide details of authority approving study; if none were required, explain why.	Not Applicable	

Dual Use Research of Concern (DURC)	Information included in the manuscript?	In which section is the information available? (Reagents and Tools Table, Materials and Methods, Figures, Data Availability Section)
Could your study fall under dual use research restrictions? Please check biosecurity documents and list of **select agents and toxins** (CDC): https://www.selectagents.gov/sat/list.htm	Not Applicable	
If you used a select agent, is the security level of the lab appropriate and reported in the manuscript?	Not Applicable	
If a study is subject to dual use research of concern regulations, is the name of the **authority granting approval and reference number** for the regulatory approval provided in the manuscript?	Not Applicable	

Reporting

The MDAR framework recommends adoption of discipline-specific guidelines, established and endorsed through community initiatives. Journals have their own policy about requiring specific guidelines and recommendations to complement MDAR.

Adherence to community standards	Information included in the manuscript?	In which section is the information available? (Reagents and Tools Table, Materials and Methods, Figures, Data Availability Section)
State if relevant guidelines or checklists (e.g., **ICMJE, MIBBI, ARRIVE, PRISMA**) have been followed or provided.	Not Applicable	
For **tumor marker prognostic studies,** we recommend that you follow the **REMARK** reporting guidelines (see link list at top right). See author guidelines, under 'Reporting Guidelines'. Please confirm you have followed these guidelines.	Not Applicable	
For **phase II and III randomized controlled trials**, please refer to the **CONSORT** flow diagram (see link list at top right) and submit the CONSORT checklist (see link list at top right) with your submission. See author guidelines, under 'Reporting Guidelines'. Please confirm you have submitted this list.	Not Applicable	

Data Availability

Data availability	Information included in the manuscript?	In which section is the information available? (Reagents and Tools Table, Materials and Methods, Figures, Data Availability Section)
Have **primary datasets** been deposited according to the journal's guidelines (see 'Data Deposition' section) and the respective accession numbers provided in the Data Availability Section?	Yes	Data Availability
Were **human clinical and genomic datasets** deposited in a public access-controlled repository in accordance to ethical obligations to the patients and to the applicable consent agreement?	Yes	Data Availability
Are **computational models** that are central and integral to a study available without restrictions in a machine-readable form? Were the relevant accession numbers or links provided?	Not Applicable	
If publicly available data were reused, provide the respective **data citations in the reference list**.	Not Applicable	
